# Evaluation of different QuEChERS-based methods for the extraction of 48 wastewater-derived organic contaminants from soil and lettuce root using high-resolution LC-QTOF with MRM^HR^ and SWATH acquisition modes

**DOI:** 10.1007/s11356-024-32423-w

**Published:** 2024-02-19

**Authors:** Nicola Montemurro, Rayana Manasfi, Serge Chiron, Sandra Perez

**Affiliations:** 1 Environmental and Water Chemistry for Human Health (ONHEALTH), Institute of Environmental Assessment and Water Research (IDAEA-CSIC), c/Jordi Girona 18-26, 08034 Barcelona, Spain; 2 HydroSciences Montpellier (HSM), University of Montpellier, Building 39 - CC57 300, Avenue du Professeur Emile Jeanbrau, 34090 Montpellier, France

**Keywords:** Pharmaceuticals, Wastewater reuse, Soil contamination, Root uptake, Modified QuEChERS, LC-HRMS/MS

## Abstract

The reuse of treated wastewater in agriculture is an important route of introducing a large number of organic contaminants into the agroecosystem. In this study, a modified QuEChERS-based approach was developed for rapid, simple, and simultaneous extraction of 48 organic wastewater-derived contaminants from soil and lettuce root. Twenty-two different (modification) scenarios of the known (or original) QuEChERS method have been tested, in order to obtain best and well-compromised recoveries for all target compounds for soil and roots. Finally, a common method was chosen for both matrices consisting of a single extraction step using EDTA-Mcllvaine buffer and the unbuffered Original QuEChERS salts. Method performance was accomplished by liquid chromatography coupled with high-resolution mass spectrometry on a QToF-MS system using two different acquisition modes, the ultra-fast high-resolution multiple reaction monitoring (MRM^HR^) mode and the innovative Sequential Window Acquisition of All Theoretical Fragment-Ion (SWATH) mode. Performance characterization was evaluated in terms of recovery, linearity, intra-day precision, method detection limits (*MDLs*), method quantification limits (*MQLs*), and matrix effect (ME). Recoveries in MRM^HR^ mode ranged from 63 to 111% and 54 to 104% for lettuce root and soil, respectively, for most of compounds in MRM^HR^ mode and from 56 to 121% and 54 to 104% for lettuce root and soil, respectively, for most of compounds in SWATH. Whereas, *MQLs* ranged from 0.03 to 0.92 ng g^−1^ in MRM^HR^ and from 0.03 to 82 ng g^−1^ in SWATH for lettuce root, and from 0.02 to 0.44 ng g^−1^ in MRM^HR^ and 0.02 to 0.14 ng g^−1^ in SWATH for soil. The method was then applied to follow the target compounds in soil and lettuce root, where the system lettuce-soil was irrigated with treated wastewater under real greenhouse conditions. Five and 17 compounds were detected in lettuce root and soil, respectively.

**Graphical Abstract:**

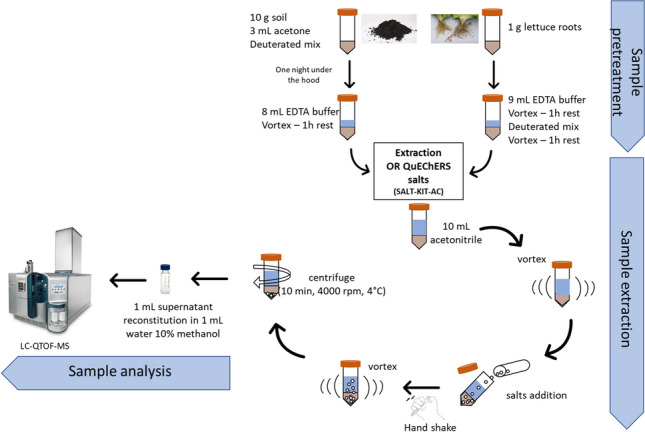

**Supplementary Information:**

The online version contains supplementary material available at 10.1007/s11356-024-32423-w.

## Introduction

Meeting the lack of water for agricultural purposes is a global problem today. Current increases in temperatures and changes in precipitation make the Mediterranean region one of the most vulnerable areas to climate change, and it is predicted that future water supply is likely to be even more compromised by population growth and climate change and which, together to the incessant water pollution, may represent an impediment to the correct functioning of the communities (Lavrnić et al. 2017). To alleviate this problem, countries in arid and semi-arid areas use treated wastewater (TWW) for crop irrigation as an alternative to natural water resources. Although water reuse can have positive effects on the environment, such as reducing the withdrawal of fresh water from sensitive ecosystems or the discharge of treated wastewater into water bodies (Lonigro et al. 2016), this practice constitutes an important entry route for organic micropollutants such as pharmaceutical active compounds into the soil and crops (Martínez-Piernas et al. 2018b; Mordechay et al. 2018; Petrie et al. 2015), as traditional wastewater treatment plants (WWTPs) are not designed for such purposes, ensuring in some cases only partial removal of organic micropollutants. Once these pollutants are spread on agricultural soil through the reuse of wastewater, they can be accumulated in the soil and/or taken up by plants through the root system (Carter et al. 2018). Based on their physicochemical properties, some of them remain confined to the rhizosphere. Others, on the other hand, have the potential to be taken up by plant roots (Wu et al. 2015), and be translocated to the aerial parts with consequent introduction of undesirable compounds into the food chain and potential risks for human health, as well as for the health of the ecosystem (Ahmed et al. 2015; Christou et al. 2017; Fu et al. 2019; García et al. 2019; Li et al. 2019; Picó et al. 2019; Tian et al. 2019). In fact, the roots constitute the main point of entry of contaminants through the soil, regulating the translocation of pollutants to the leaves and fruits (Ahmed et al. 2015; Wu et al. 2010). Therefore, pollutants that are unable to reach the leaves/edible parts either accumulate in the roots or remain confined to the soil (Wu et al. 2015). For example, Beltrán and colleagues found the highest concentration of triclosan in the root part of corn (Beltrán et al. 2020).

Understanding the accumulation of pollutants in soil and plant tissues such as roots, leaves, and fruits is crucial for a better assessment of the risk to human health following irrigation of vegetable crops with TWW. Also because the new European legislation on the minimum requirements applicable to recovered water intended for agricultural irrigation once again does not consider organic pollutants of anthropic origin (Commission 2020). Furthermore, in order to respond to the scientific need for risk assessment, simple, fast, cheap but reliable extraction methods are required depending on the matrix under study. Environmental samples are known to exhibit numerous analytical complexities. Each of these samples has very different constituents of the matrix that are involuntarily co-extracted during sample preparation, and their presence in the extracts can mask the detection of the analytes of interest during chromatographic analysis (Stoob et al. 2006). For example, the soil is rich in organic matter, humic, and fulvic acids, while the roots of vegetables, especially lettuce, are the main source of carbohydrates and exudates including amino acids, amides, sugars and sugar alcohols, and many organic acids as well as ornithine, urea, benzoic, and lauric acids (Neumann et al. 2014). These matrix components establish different interactions with target analytes and influence their extraction yields. Therefore, specific and rigorous extraction methods and cleanup procedures are required for each of these matrices.

In addition to sample preparation and extraction, LC-MS/MS analysis as well as data analysis are also time-consuming (Manasfi et al. 2021b). So, having a common extraction method for multiple matrices is very beneficial. It provides time-saving and less complications for the analyst who performs the extraction which use fewer objects because the same equipment and laboratory materials will be used for both matrices. In addition, the detection of these pollutants in environmental matrices, such as water, soil, plants, or biota, is challenging due to their presence at low concentrations, and consequently to the low detection limits required for their analysis.

Several extraction methods have been reported for organic pollutants derived from wastewater from water (Miossec et al. 2019), soil (Kumirska et al. 2019), sediments (Nannou et al. 2019), and crops (Martínez-Piernas et al. 2018b). In particular, in recent years, various techniques have been introduced for the analysis of wastewater-derived organic pollutants in soil samples, such as pressurized liquid extraction (PLE) (Biel-Maeso et al. 2017; Durán-Alvarez et al. 2009; Vazquez-Roig et al. 2010), microwave-assisted extraction (MAE) (Azzouz and Ballesteros 2012; Rice and Mitra 2007), and ultrasound-assisted extraction (UAE) (Albero et al. 2015; Montemurro et al. 2019; Xu et al. 2008), which allowed to obtain high extraction recoveries. However, these techniques are time, materials, and solvents consuming, and require advanced instrumentation and analysts with high expertise to manipulate them (Manasfi et al. 2021b).

Lettuce crop is the most cultivated plant worldwide, it grows quickly in greenhouse and open fields, and it has a complex roots’ structure that may facilitate the uptake of organic contaminants from soil (Bigott et al. 2021; Chuang et al. 2019; Manasfi et al. 2021b; Martínez-Piernas et al. 2018b; Montemurro et al. 2020; Montemurro et al. 2017). Moreover, the roots of herbaceous plants and vegetables are intimately in contact with the soil, placing themselves in the first 50 cm of depth. In particular, the lettuce has a root system with a short tap-root from which depart numerous thin roots that remain superficial. This feature means that separating the roots from the soil without damaging them is quite complicated. Furthermore, lettuce roots have no commercial or nutritional value, so even the possibility of obtaining real field samples of roots exposed to organic contaminants is quite limited and remains confined only to few hydroponic, greenhouse, or small-scale studies (Chen et al. 2021; Matamoros et al. 2022; Rhodes et al. 2021; Shen et al. 2021). For this reason, according to our literature survey, although lettuce is a model plant for studying the absorption of organic contaminants at the leaf level, there is a lack of studies relating to the development of specific extraction methods for lettuce roots which simultaneously allow to evaluate the two compartments, leaves and roots (over and underground). Furthermore, although the QuEChERS (quick, easy, cheap, effective, rugged, and safe) method is known for its simplicity and speed of execution, and despite the soil being a fairly studied matrix previously, however, to our knowledge, only few studies have been conducted for the extraction of wastewater-derived organic compounds from the soil using this method (Bragança et al. 2012; De Carlo et al. 2015; Hang et al. 2021; Lee et al. 2017; Meng et al. 2017; Salvia et al. 2012). However, none of the previously mentioned studies are able to extract a large number of organic micropollutants or different chemical classes simultaneously.

Considering the limitations mentioned above, there is a clear need to develop a common extraction method applicable at the same time for both matrices, soil and the lettuce roots. In this context, the aim of our study was to develop and validate a fast and easy multi-residue extraction method for lettuce roots and soil, using a modified QuEChERS approach in order to facilitate the detection and quantification of 48 organic pollutants usually present in wastewater and belonging to very different families such as pharmaceutical products, fungicides, food, and industrial additives between the soil-root system for a better risk assessment for human health or the environment. A different QuEChERS method for lettuce leaves was developed based on the different needs of extraction yields and matrix cleanup. Further details about lettuce leaves extraction method are available in our previous study (Montemurro et al. 2020).

Finally, method performance characterization were performed on a SCIEX X500R LC-QTOF-MS hybrid system, comparing two different high-resolution mass spectrometry modes: high-resolution multiple reaction monitoring (MRM^HR^) and the Sequential Window Acquisition of All Theoretical Fragment-Ion (SWATH) acquisition modes. The performance of 22 different modified QuEChERS approaches was performed by varying the pH of the extraction solvent, QuEChERS salts, water content, and cleanup sorbents. After characterization, the optimized analytical methods were applied to the analysis of the selected compounds in real soil and lettuce root samples irrigated with TWW under greenhouse conditions.

## Experimental section

### Material and reagents

Commercially available Original unbuffered QuEChERS salts (OR-a, containing 4 g MgSO_4_ + 1 g NaCl, and OR-b, containing 6 g MgSO_4_ + 1.5 g NaCl), the citrate buffered European EN 15662 salts (CEN, containing 4 g MgSO_4_ + 1 g NaCl + 0.5 g disodium citrate sesquihydrate) used for salting out extraction process, and dispersive solid phase extraction (dSPE) sorbents for the cleanup step (900 mg MgSO_4_ + 150 mg PSA + 150 mg C18) were supplied from BEKOlut GmbH & Co. KG (Hauptstuhl, Germany). Ammonium acetate (NH_4_CH_3_CO_2_), ammonium formate (NH_4_HCO_2_), sodium sulfate (Na_2_SO_4_), and sodium chloride (NaCl) were supplied from Sigma-Aldrich (St. Louis, MO, USA).

LC-MS grade acetonitrile (ACN) (≥99.9%), methanol (MeOH) (≥99.9%), ethyl acetate (EtAc) (≥99.9%), dimethyl sulfoxide (DMSO) (≥99.9%), and HPLC water were purchased from Merck (Darmstadt, Germany). Mobile phase additives formic acid (≥96%, ACS reagent) and ammonium acetate were supplied by Sigma-Aldrich while ammonium fluoride was bought from Fisher Chemical (Fisher Scientific SL, Madrid, Spain). For high-purity mobile phase solutions, ACN and water (Optima™ LCMS Grade) were purchased from Fisher Chemical (Fisher Scientific SL, Madrid, Spain).

Di-sodium hydrogen phosphate dihydrate (Na_2_HPO_4_·2H_2_O), citric acid monohydrate (C_6_H_8_O_7_·H_2_O), and anhydrous ethylenediamine tetraacetic acid (EDTA) (≥99%) for the EDTA-McIIvaine buffer (pH 4) preparation (Supplementary Information, SI) were obtained from Sigma-Aldrich (St. Louis, MO, USA).

High-purity (˃97%) reference standard of all target compounds (acesulfame, acetaminophen, acridone, benzotriazole, 5-methyl-2H-benzotriazole, bezafibrate, bisphenol A, caffeine, carbamazepine, carbamazepine-10,11-epoxide, chloramphenicol, ciprofloxacin, citalopram, clarithromycin, climbazole, clofibric acid, diclofenac, 4-hydroxydiclofenac, diltiazem, fenofibrate, fipronil, fipronil desulfinyl, fipronil sulfone, fluconazole, furosemide, gemfibrozil, hydrochlorothiazide, ibuprofen, indomethacin, irbesartan, lamotrigine, lamotrigine N2-oxide, 5-desamino-5-oxo-2,5-dihydro lamotrigine, methadone, metoprolol, metronidazole, N2-methyl-lamotrigine, N4-acetyl-sulfamethoxazole, oxcarbazepine, propranolol, sucralose, sulfamethazine, sulfamethoxazole, 4-nitro-sulfamethoxazole, sulfanilamide, sulfanilic acid, valsartan, valsartan acid, verapamil) were purchased from Sigma-Aldrich (St. Louis, MO, USA). Analytical standards were individually weighted and dissolved in 100% ACN, 100% HPLC water, 100% MeOH, or 100% DMSO, at a concentration of 5 or 10 mg mL^−1^, according to compound solubility, and stored in the dark at −20 °C. The CAS numbers, molecular formulas, molecular weight, and other relevant properties of all target compounds are reported in Table S1.

The corresponding isotope-labelled internal standards (IS) used as surrogates were obtained from Cerilliant (Round Rock, TX, USA), Alsachim (Illkirch-Graffenstaden, France), Santa Cruz Biotechnology (Dallas, TX, USA), or Toronto Research Chemicals (Toronto, ON, Canada) as solutions at a concentration of 1 mg mL^−1^ or prepared in MeOH or DMSO at a final concentration of 1 mg mL^−1^. The complete list of IS compounds were reported in Table S3.

Working solution mixtures used for analysis and calibration purposes were prepared separately for all compounds and labelled standards by serial dilution in MeOH at concentration of 2 μg mL^−1^ and stored in the dark at −20 °C.

### Sample pretreatment and extraction

#### Lettuce root samples

Given the difficulty in finding enough contaminants-free roots for recovery tests, several lettuce seedlings (*Lactuca sativa* L., “Maravilla de verano-Canasta” sp.) at the approximately four-leaf stage were purchased from a local garden center in Barcelona (Spain) and were put to grown in controlled condition according to a previous study (Montemurro et al. 2020). At maturity stage, lettuce crops were collected and the roots were separated from the rest of the leaves, accurately washed and blotted dry, and subsequently freeze dried using a LyoAlfa 6 system (Telstar Technologies, Terrassa, Spain). Thereafter, dried roots were homogenized pooled together using a knife mill with a stainless-steel grinding chamber (Grindomix GM 200, Retsch GmbH, Haan, Germany) and stored in the dark at −40 °C until method optimization and characterization.

#### Lettuce root pretreatment and spiking

**Fig. 1:**
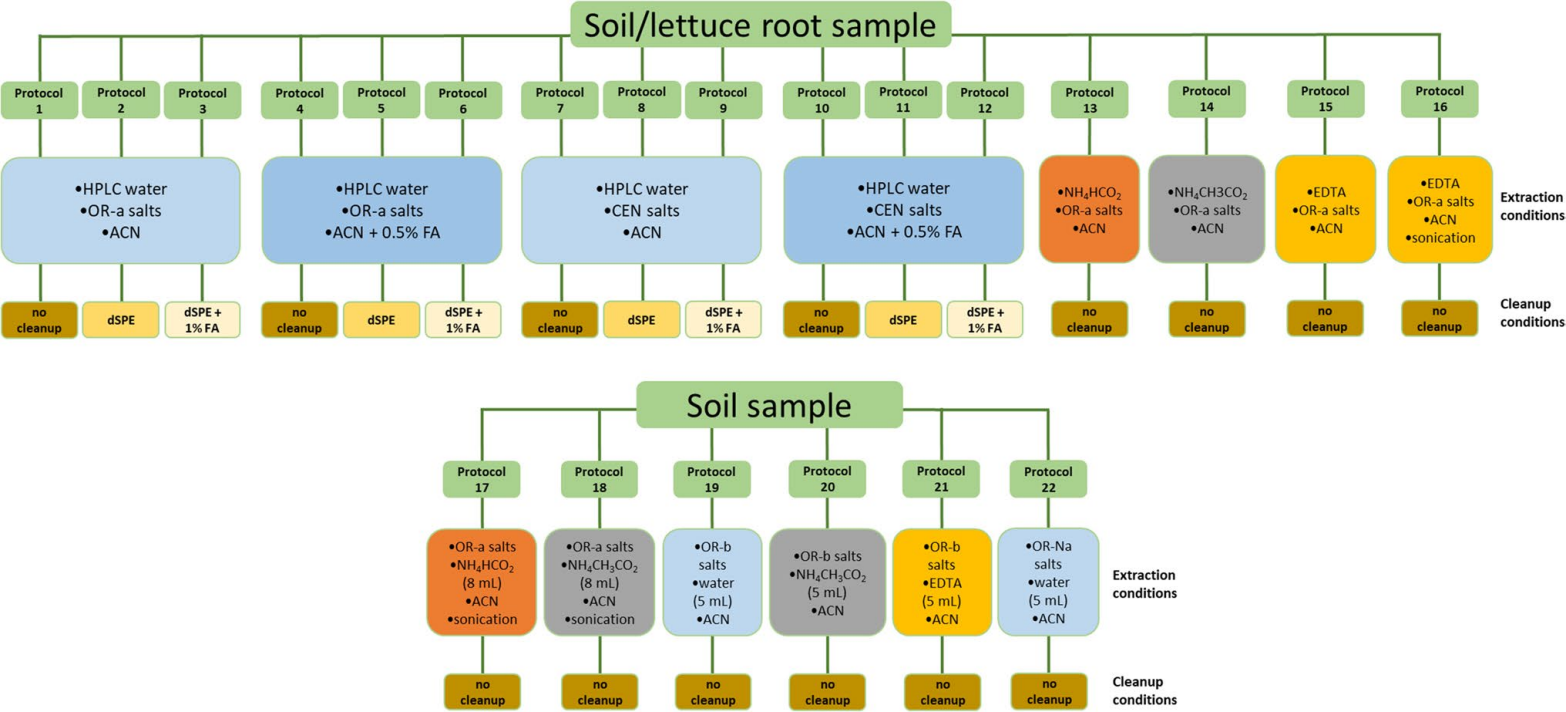
Extraction and cleanup condition of protocols 1 to 22 for lettuce root and soil matrices (protocols 17 to 22 are for soil matrix only)

For recovery study, 1 g of freeze-dried lettuce roots was weighted in 50-mL centrifuge tube; hydrated with 9 mL either HPLC-water, NH_4_CH_3_CO_2_, NH_4_HCO_2_, or EDTA-Mcllvaine buffer; vortexed; and left to rest for 1 h until completely hydrated. Thereafter, root samples were spiked with 50 μL of working mixture solution containing all target compounds (2 μg mL^−1^) to achieve a final concentration of 100 ng g^−1^ dry weight (d.w.), corresponding to 10 ng g^−1^ of fresh weight (f.w.). Then samples were vortexed again and rested for another hour. A summary scheme of all the protocols used for root extraction is shown in Fig. 1 and Table S2.

#### Soil sampling

The contaminant-free soil used in this study was collected from a pristine area of the Parc Agrari of El Prat de Llobregat (Barcelona, Spain). Soil was left in a tray under the fume hood for 2–3 days to ensure its total dryness. Thereafter, it was homogenized by a mortar and sieved at 2-mm pore size to remove coarse particles and increase its homogeneity. Finally, it was stored in the dark at −40 °C until method optimization and characterization.

#### Soil pretreatment and spiking

To test different procedures, 10 g of soil was weighted in a 50-mL centrifuge tube. Then, 3 mL of acetone was added followed by 50 μL of working mixture solution (2 μg mL^−1^) to achieve the desired concentration of 10 ng g^−1^ (d.w.). The tubes were then vortexed and left under the hood at room temperature for one night, to allow solvent evaporation and compound interaction with soil. Consequently, soil samples were hydrated by adding 8 mL of HPLC water, NH_4_CH_3_CO_2_, NH_4_HCO_2_, or EDTA-Mcllvaine buffer to achieve at least 80% hydration for the salting step. A detailed list of all protocols used for soil extraction is shown in Fig. 1 and Table S2. Additional protocols (17–22) were tested for soil only. According to Table S2, only 5 mL of hydration solution was used for protocols 19, 20, 21, and 22. Finally, the tubes were vortexed, and rested for 1 h before extraction step.

#### Sample extraction

Recoveries of all target compounds were optimized following the different protocols reported in Fig. 1 (see Table S2 for further extraction and cleanup details). A common modified QuEChERS method was finally selected for both matrices (protocol 15), which consisted of one single extraction step according to the following protocol. Briefly, 10 mL of ACN was added to the hydrated sample (EDTA-Mcllvaine buffer) and vortexed. Then the original QuEChERS salt tube (OR-a, containing 4 g MgSO_4_ + 1 g NaCl) was emptied in the mixture of ACN and the obtained mixture was immediately hand shaken for 1 min in order to prevent salt agglomeration then vortexed for another minute. Finally, the tube was centrifuged for 10 min at 4000 rpm and 4 °C and 1 mL of the obtained supernatant was evaporated under gentle nitrogen flow at room temperature until total dryness. Residues were resuspended with 1 mL of water/MeOH (90/10, v/v) for injection. Additionally, three more tubes for both matrices were treated the same way excluding the spiking of standard mix, in order to obtain a ‘blank extract’ for characterization and calibration purpose.

### LC-MS/MS analysis

Analysis of pharmaceutical residues in soil and root samples was based on the comparison of high-resolution multiple reaction monitoring (MRM^HR^) and SWATH acquisitions, both performed by a SCIEX X500R QTOF system (Sciex, Redwood City, CA, USA). Chromatographic separation was performed with a Merck Hibar HR Purospher STAR RP-C18 column. All information about chromatographic separation, mobile phases used for the positive electrospray ionization mode (ESI+) and negative electrospray ionization mode (ESI−), the elution gradient, the source conditions, the mass correction (calibration), and any detailed information regarding LC-MS/MS methodology are described in Supplementary Information or elsewhere (Montemurro et al. 2021; Montemurro et al. 2020).

### Method performance characterization

The performance of the final selected methods (protocol 15 for both matrices) was validated according to the following factors: accuracy, intra-day precision, matrix effect (ME), linearity, method detection limits (*MDLs*), and method quantification limits (*MQLs*). The performance of the method was also compared using both MRM^HR^ and SWATH acquisition modes.

#### Accuracy

The accuracy of the methods was expressed as relative recoveries (*RR%*) by spiking both matrices at five concentration levels (2, 5, 10, 50, 200 ng g^−1^ f.w. for lettuce root and 2, 5, 10, 50, 200 ng g^−1^ d.w. for soil, respectively) and were determined by the mean value of a triplicate (*n* = 3) for each concentration level. The obtained mean areas were compared to the mean areas of a triplicate set of blank extracts spiked with the same concentration levels. Finally, *RR%* was calculated using the following equation (Eq. 1):

1 $$RR\ \left(\%\right)=100\times \left( area\ of\ spiked\ sample/ area\ of\ spiked\ blank\ extract\right)$$

#### Intra-day precision

The repeatability of the method (intra-day precision) was evaluated by calculating the relative standard deviation (RSD%) obtained from the relative recoveries for the abovementioned five concentration levels. Each concentration was evaluated in triplicate.

#### Matrix effect (ME %)

For matrix effect evaluation (signal enhancement or suppression), a set of blank extracts (*n* = 3) for each concentration level and for both matrices were prepared following the final selected method (protocol 15). Aliquots of 1 mL of extracts were spiked just before LC-MS/MS analysis at 2-, 5-, 10-, 50-, and 200-ng g^−1^ concentration level. Similarly, aliquot of 1 mL of injection solvent (water/MeOH 90:10) was spiked under the same conditions. Finally, *ME (%)* was calculated using the following equation (Eq. 2):

2 $$ME\ \left(\%\right)=100\times \left[\left( area\ of\ spiked\ blank\ extract/ area\ of\ spiked\ solvent\right)-1\right]$$

It should be noted that *ME (%)* higher than ǀ40%ǀ were considered as high impact on the performance of the method (Labad et al. 2021; Montemurro et al. 2020).

#### Linearity

Linearity of the instrumental response was evaluated using a matrix-matched calibration curve with concentration range from 0.05 to 300 ng mL^−1^, corresponding to 0.5 to 3000 ng g^−1^ f.w. for lettuce root and 0.05 to 300 ng g^−1^ d.w. for soil, respectively. Calibration curves were constructed using by linear weighted least-squares regression (1/*x* as weighting factor) by plotting the ratio of the analyte signal to that of its corresponding deuterated compound (Table S3). At least 8 calibration points per curve were considered. Finally, linearity was evaluated by calculating the coefficient of determination (*r*^2^) for each analyte in each matrix where *r*^2^ ≥ 0.99 is the acceptance criterion.

#### Method detection and quantification limits

*MDLs* and *MQLs* were determined as the concentration level that gave a peak signal 3 times and 10 times the background noise from the chromatogram, respectively. *MDL* and *MQL* were estimated from the matrix-matched calibration curves using the following equations (Eqs. 3 and 4) according to our previous works (Montemurro et al. 2021; Montemurro et al. 2020):

3 $$MDL=3\times \left( Sb/ slope\right)$$

4 $$MQL=10\times \left( Sb/ slope\right)$$

where *S*_b_ is the standard deviation of the intercept.

### Application to real samples

The developed method was applied to real soil and lettuce root samples, irrigated with TWW under greenhouse conditions. The greenhouse was located at IDAEA-CSIC facilities (Barcelona, Spain), where lettuces were grown in 12 plastic pots (22-cm diameter). Lettuce seedlings (*Lactuca sativa* L., var. Maravilla de Verano-Canasta) were obtained from a local plant nursery. Lettuce plants were placed in each pot and cultivated for 60 days to get maturity stage. Approximately 100 mL of treated wastewater effluent provided by the municipal wastewater treatment plant of El Prat de Llobregat (Barcelona, Spain) was spread on soil surface each 2 days, making root uptake the only route of exposure to organic contaminants. Concentrations of organic contaminants were evaluated according to Sabater-Liesa et al. (2019) and Sabater-Liesa et al. (2021) and were reported in Table S12. Four pots were irrigated only with tap water and used as control. More details regarding the experimental design are reported elsewhere (Montemurro et al. 2020). After 60 days, the whole lettuces were harvested, and its surrounding soil was sampled. All pots were transported directly to the laboratory, where roots and soil were prepared according to the “Sample pretreatment and extraction” section. Whereas, lettuce plants were carefully hand washed with tap water, rinsed with purified water, and the roots were separated from the leaves and blotted dry with a paper tissue (Figure S1). Then, lettuce roots were freeze-dried and ground to a fine powder with a knife mill with a stainless-steel grinding chamber (Grindomix GM 200, Retsch GmbH, Haan, Germany) and stored at −20 °C until extraction. The selected protocols were applied to extract organic contaminants from real samples. Before performing the salting out step, 50 μL of internal standards works as mixture solution (2 μg mL^−1^) to achieve a final concentration of 10 ng g^−1^ in both matrices. Finally, both MRM^HR^ and SWATH modes were used for the quantification of organic contaminants in the obtained real samples.

## Results and discussion

### Modified QuEChERS method development

The selection of the investigated wastewater-derived organic compounds was based on their occurrence in treated wastewater, and their previous detection in edible crops (Christou et al. 2019). Additionally, they cover a wide range of polarity (log *P* = −2.16 to log *P* = 5.31) (Tab S1), which made method development more difficult (Manasfi et al. 2021b). They include a wide range of therapeutical classes such as analgesics and antiinflammatories, antihypertensives, antifungal agents, lipid regulators, psychiatric drugs and stimulants, β-blockers, antibiotics, and sweeteners with different characteristics and physical/chemical properties (Tab S1). Furthermore, their adsorption varies according to the compound and absorption is often controlled by interactions with specific or complicated functional group pH-dependent speciation (Golovko et al. 2016).

**Fig. 2:**
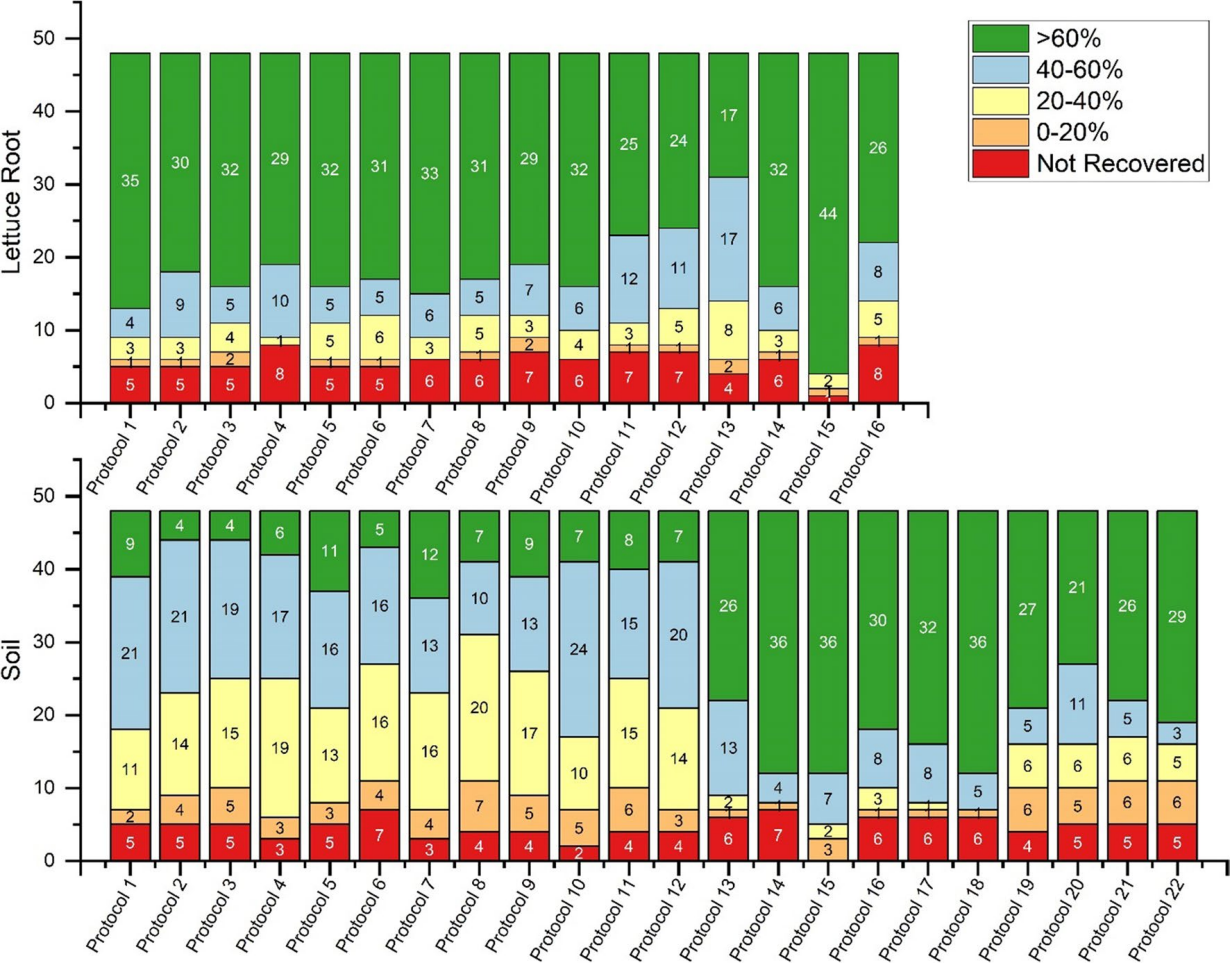
Number of compounds with recoveries >60%, 40–60%, 20–40%, 0–20%, and not recovered for lettuce root and soil matrices

To save time, money, and reagents, the comparison of all the tested protocols to choose the best ones was done by injecting the samples only in MRM^HR^ acquisition mode, whereas for the performance characterization both acquisition modes were performed and studied. To obtain well-compromised recoveries for all of them, different extraction salts, pH conditions, and an additional dispersive SPE cleanup step were tested. Each protocol for each matrix was tested in triplicate. Details of all implemented protocols are shown in Table S2 for both matrices, lettuce root and soil. The obtained results and hereafter discussed are shown in details in Fig. 2 and Table S4 to Table S11 for lettuce root and soil, respectively. A statistical comparison between the different protocols and matrices, using a loss function to optimize the protocol selection, was also reported in Figure S4.

Although the sample preparation for lettuce root and soil is different, for simplicity and affinity the same extraction criteria were applied for both as reported in the “Experimental section.” Hydration is considered as one of the most important steps in QuEChERS method, with which water molecules can interact and bend with the adsorption sites of the matrix promoting the desorption of analytes. Hence, 9 mL of hydration solution was used for lettuce root based on the original QuEChERS method developed by Anastassiades and co-workers (Anastassiades et al. 2003), where they referred to the natural hydration percentage (90%) of fruits and vegetables. On the other hand, only 8 mL of hydration solution was used for soil referring to the minimum hydration percentage applied in QuEChERS methodology. Recent studies showed the importance of the hydration step. For instance, De Carlo and co-workers (De Carlo et al. 2015) tried several extraction solvents for the extraction of bentazone, atrazine, carbamazepine, phenytoin, and its metabolite 5-(p-hydroxyphenyl-),5-phenylhydantoin from soil using QuEChERS. Their results showed that the addition of water to the extraction solvent significantly enhanced the recovery of the analytes except for atrazine. Consequently, in the present study different hydration volumes or solutions were evaluated in combination with different QuEChERS extraction salts in soil.

#### HPLC water as hydrating solution and OR-a QuEChERS salts

In protocols 1 to 6 (Fig. 1; Table S2), HPLC water as hydrating solution and original QuEChERS unbuffered salts (OR-a, containing 4 g MgSO_4_ + 1 g NaCl) were used for both matrices. On the other hand, 10 mL either pure acetonitrile (protocols 1, 2, and 3) or acidified acetonitrile 0.5% formic acid (protocols 4, 5, and 6) was used as extraction solvent according to original QuEChERS method (Anastassiades et al. 2003). Moreover, an additional dispersive SPE cleanup step (containing 900 mg MgSO_4_ + 150 mg PSA + 150 mg C18e) was tested only for protocols 2, 3, 5, and 6. The cleanup step allows for matrix interference removal, but it can also cause the loss of analytes not only during cleanup performance but also during storage due to change of pH conditions, as reported in previous works (Anastassiades et al. 2003; Bergé and Vulliet 2015; Montemurro et al. 2020). As illustrated in Fig. 1, pure acetonitrile was used for the extraction in protocol 1, whereas in protocol 4, the extraction solvent was replaced with acidified acetonitrile (0.5% formic acid) to test the effect of acidic medium on the extraction of our compounds of interest. No cleanup step was performed in these two abovementioned protocols (1 and 4). In the case of lettuce root, results of protocols 1 and 4 showed similar overall recoveries ranging from 47.5 (lamotrigine N2-oxide) to 121.3% (4-OH-diclofenac) and from 50.8 (N2-methyl-lamotrigine) to 132.3% (valsartan) for protocols 1 and 4, respectively. However, 5 compounds (valsartan acid, ibuprofen, ciprofloxacin, fenofibrate, and indomethacin) were not extracted with protocol 1, whereas sulfanilamide and gemfibrozil showed recovery of 17 and 215%, respectively, and they were not included. Comparatively, 8 compounds (ciprofloxacin, fenofibrate, furosemide, indomethacin, sulfanilamide, propranolol, sulfamethazine, ibuprofen) were not extracted with protocol 4 (Table S4). This could be in part explained by their pKa values, with which they are positively charged under acidic condition, thus decreasing their extraction efficiency. In the case of soil (Fig. 2 and Table S5), a remarkable decrease of recoveries was observed with protocol 1 or by adding formic acid (protocol 4). In fact, the number of compounds with acceptable recoveries higher than 60% were only 9 for protocol 1 (including caffeine 64% and climbazole 107.9%) and 6 with protocol 4, including verapamil 74.1% and fipronil desulfinyl 112%. However, most compounds were recovered in the range 40–60%. Consequently, for soil protocol 1 recorded better results than protocol 4 (Figure S4).

For protocols 2 and 5, the same steps as for protocols 1 and 4 were repeated, respectively, followed by a dispersive SPE cleanup step (containing 900 mg MgSO_4_ + 150 mg PSA + 150 mg C18e), in order to test for the influenza of cleanup salts on analyte recoveries and matrix interferences. PSA (or primary secondary amines) is a polar adsorbent, and originally used to remove fatty acids and other impurities such as strong polar organic acids (Hang et al. 2021); C18 is a nonpolar adsorbent and used to remove fats, lipids, and some minerals; and finally MgSO_4_ is a conventional desiccant and used to remove water residues in organic solvents. As expected, recoveries of most compounds decreased in protocols 2 and 5 after the addition of dSPE cleanup for both matrices. The drop of recoveries after the application of a cleanup step was also observed in previous studies (Salvia et al. 2012). Specifically, the number of compounds recording recoveries higher than 60% also decreased from 35 (protocol 1) to 30 (protocol 2) in the case of lettuce root where the most affected compounds are clofibric acid that drops from 91.5 to 31.6%, 4-OH-diclofenac from 121.3 to 34%, bezafibrate from 82.6 to 39.6%, sulfanilic acid from 87.1 to 45%, irbesartan from 101.6 to 46.3%, acridone from 81.3 to 56.2%, and diclofenac from 96.5 to 58.3%. In contrast, sulfamethoxazole increased its recovery from 31.3 to 48.9%. Slightly better results were obtained in the case of protocol 5 as the addition of formic acid in the extraction solvent seems to counteract the pH increase due to PSA as previously reported (Montemurro et al. 2020). Valsartan acid and chloramphenicol resulted the most affected compounds with recoveries from 132.3 to 19.9% and from 105.4 to 38.9%, respectively. Even though, propranolol and sulfamethazine that were previously not recovered now presented values of 129.6 and 23.3%, respectively. As for the root, also for soil there is a general reduction of recoveries with only 4 compounds with recoveries higher than 60% (protocol 2) including caffeine (82%), fipronil (74.4%), fipronil desulfinyl (75.1%), and fipronil sulfone (88.7%) (Fig. 2, Fig. S4, and Table S5). In this case, the most affected compounds are valsartan and furosemide with 68.5 and 50.4% that are now not recovered together with valsartan acid, fenofibrate, and indomethacin. The same fate is reserved for these five compounds in the case of protocol 5. Despite everything, 11 compounds have recoveries greater than 60%, ranging from 93.8% of sucralose (before 31%) to 60% of bisphenol A. However, 4-OH-diclofenac and bezafibrate drastically reduced their recovery values (from 36.1 to 7.8% and 43.2 to 11.4%, respectively). As already mentioned, PSA can remove acidity from extracts and consequently raise the pH, thus increasing the risk of degradation of basic compounds. To test the effects of pH on the recoveries of the target compounds in the final extract, protocols 3 and 6 were implemented as a follow-up of protocols 2 and 5, respectively, in which 1 mL of the extract after SPE dispersive cleanup was acidified at 1% with 10 μL of formic acid to preserve the acidity of the extract. However, no relevant difference was observed between protocols 2 and 3 and protocols 5 and 6 for both matrices. So, this step was skipped. To summarize, the comparison of the results of the 6 protocols mentioned above (protocols 1 to 6) showed that protocol 1 showed the best results for both matrices, where most of the compounds were recovered over 60% in lettuce and between 40 and 60% for soil, respectively, with the least number of unextracted compounds (Fig. 2. Fig. S4, and Tables S4 and S5). Although, in protocols 1 to 6 in the case of soil, most of the compounds were extracted with recoveries of less than 60%, it should be noted that for lettuce roots the recoveries obtained in all protocols were much higher than 60%, with 29 to 35 compounds compared to 4 to 11 compounds over 60% recovered from the soil in protocols 1 to 6. This can be explained by the high complexity of the soil matrix, where soil properties influence the sorption of compounds resulting in greater retention (Kodešová et al. 2015; Kodešová et al. 2016). In general, the use of acidified acetonitrile lowered the recoveries of most compounds contrary to previous studies (Bragança et al. 2012; De Carlo et al. 2015; Montemurro et al. 2019) where recoveries were significantly improved after acid addition. Furthermore, for both matrices, further dSPE cleanup step generally reduced compound recoveries, and addition of formic acid to the final extract after purification showed no relevant differences. Therefore, protocol 1 still had the best recoveries (Fig. S4).

#### HPLC water as hydrating solution and EN QuEChERS salts

The target compounds have different pKa ranging from −4.9 to 15.96 (Tab S1). Furthermore, especially soil samples can have different pH depending on their origin. For this reason, we consider to test the addition of a buffer during the extraction under the same conditions mentioned above. The same 6 protocols were repeated using the European standard EN QuEChERS involving the use of citrate buffer (CEN, 4 g MgSO_4_ + 1 g NaCl + 0.5 g disodium citrate sesquihydrate, pH = 5–5.5) (protocols 7 to 12 in Fig. 1). In the case of roots, in general the citrate buffer showed both a number of compounds recovered and slightly lower recovery values than the use of OR-a (Nannou et al. 2019). Additionally, RSD% values were found to be above 30% for most compounds. This is particularly true in the case of soil where, with the exception of protocol 10, most of the compounds show recoveries between 20 and 40% (Fig. 2). In the case of protocol 10 in which no cleanup was performed, the use of acidified ACN (0.5%) improves the extraction of all compounds with 50% of them having recoveries between 40 and 60% ranging from 39.3% of carbamazepine epoxide to 113.7% of sulfanilic acid. Only two compounds remain excluded with this protocol (fenofibrate and indomethacin). However, protocol 1 was still showing similar or even better results than protocols 7 and 10.

#### Buffer solutions as hydrating solution and OR-a QuEChERS salts

To improve the recoveries of the analytes obtained in protocol 1, we replaced the HPLC water with three different buffer solutions used by previous studies (Bian et al. 2015; González-Curbelo et al. 2014; Guo et al. 2016; Hang et al. 2021; Kachhawaha et al. 2017) and we compared for the extraction efficacy. We used ammonium formate in protocol 13, ammonium acetate in protocol 14, and EDTA-Mcllvaine buffer in protocol 15 (Fig. 1) with the OR-a salt kit. In the case of roots, a clear reduction in recovery is noted, especially when ammonium acetate is used. Indeed, compared to protocol 1, the use of ammonium acetate reduced well-recovered compounds (>60%) from 72.9 to 35.4%. Conversely, a notable increase of compounds recovered was recorded for soil where from 18.8% of protocol 1 we passed to 54.2% in the case of ammonium and 75% in the case of formate with an overall increase of 56% in this last case. Recoveries were greatly improved with the use of EDTA-Mcllvaine Hydration Solution for both matrices. In fact, as illustrated in Fig. 2, protocol 15 using EDTA buffer solution gave the best results for both matrices, where recoveries and number of compounds above 60% recovery were even higher than protocol 1. Specifically, for the roots as many as 44 out of 48 compounds tested (about 92%) showed recoveries between 63% (verapamil) and 121% (indomethacin) and RSD% lower than 20% for most of the compounds. Among the remaining compounds, sulfamethoxazole, ciprofloxacin, and sulfamethazine had recoveries of 30.6%, 22.3%, and 17.4%, respectively. Only sulfanilamide could not be recovered. Concerning the soil, 100% of the compounds were positively recovered with 75% of the compounds having values between 60% (benzotriazole) and 104% (bisphenol A). Seven compounds (14.6%) exhibit recoveries between 45.8% (sulfamethoxazole) and 56.2% (propranolol); two compounds, 4-OH-diclofenac and sulfamethazine, with recoveries of 24.9 and 36.1%, respectively; while the remaining sulfanilamide, carbamazepine epoxide, and ciprofloxacin with values of 16.7%, 6.6%, and 4.8%, respectively. Although, the latter two cannot really be considered well recovered. Hence, the use of EDTA-McIlvaine buffer (pH = 4) in protocol 15 significantly improved the extraction efficiency of the studied compounds especially from soil, where a complexing agent (EDTA), which can break down the chelating effect, was added to facilitate the extraction of bound compounds by avoiding the complexation of these analytes with bivalent cations such as Mg^2+^ or Ca^2+^ normally present in the soil (Bian et al. 2015; Salvia et al. 2012). Similar results were obtained for extracting antibiotics (tetracyclines and quinolones) from soil using QuEChERS where the use of EDTA competed with the tetracyclines and quinolones to form complexes with metals (Bian et al. 2015; Hang et al. 2021). In fact, above all antibiotics have a strong adsorption capacity on the soil due to the presence of polarity/ionic functional groups in their chemical structures. Satisfactory results were also obtained in our previous work with earthworms (Montemurro et al. 2021). However, EDTA-McIlvaine buffer was discarded for extraction because the presence of EDTA reduced the effectiveness of the purification step, which was essential for very complex matrices such as earthworms. Conversely, rehydrating with EDTA-McIlvaine buffer was selected as the optimal extraction process in this study. However, since one of our compounds of interest (ciprofloxacin) was not adequately recovered from the soil, and poorly extracted from roots, to improve the ciprofloxacin recoveries obtained from protocol 15, a 5-min sonication step was tested immediately after the addition of acetonitrile and before the addition of the extraction salts (protocol 16 in Fig. 1). No significant improvement was observed. In fact, as reported in Bourdat-Deschamps et al. (2014), the addition of MgSO_4_ counterbalances the effect of EDTA and prevents any possible improvement in the recovery of compounds such as fluoroquinolones. On the contrary, after the ultrasonic bath, as many as eight and six compounds, respectively, for roots and soil are no longer recoverable, probably due to the non-selective ultrasonic extraction process which also favors the extraction of matrix coeluting interferents (Montemurro et al. 2017). So, this step was discarded.

#### Improving soil extraction: buffered hydrating solution and 3 different OR QuEChERS salts (OR-a, OR-b, and OR-Na)

Only for soil, six more methods were tested in order to improve the recoveries and to extract ciprofloxacin (protocols 17 to 22; Fig. 1, Fig. 2, Fig. S4, and Table S5). In protocols 17 and 18, we re-tested protocols 13 and 14, respectively, with ammonium formate and ammonium acetate, by adding a 5-min sonication step after acetonitrile addition. In protocols 19, 20, and 21, we replaced OR-a salts (4 g MgSO_4_ + 1 g NaCl) with OR-b (6 g MgSO_4_ + 1.5 g NaCl), and we also reduced hydration volume from 8 to 5 mL of HPLC water (protocol 19), ammonium acetate (protocol 20), and EDTA-Mcllvaine buffer (protocol 21). The increase of salts mass and the decrease of hydration solution volume was suggested to increase the partitioning of the analytes in the organic solvent (acetonitrile). Finally, in order to avoid the complexation of analytes (including ciprofloxacin) with bivalent cations such as Mg^2+^ also present in QuEChERS salts, we prepared an in-house salt mix containing sodium instead of magnesium (6 g Na_2_SO_4_ and 1.5 g NaCl; protocol 22). Results are shown in Fig. 2 and Fig. S4. Protocols 17 to 22 failed to extract ciprofloxacin; moreover, obtained recoveries were in general lower than the recoveries obtained with protocol 15.

Therefore, based on the above discussion, protocol 15 with EDTA-Mcllvaine buffer, acetonitrile as extraction solvent and original QuEChERS salts (OR-a, containing 4 g MgSO_4_ + 1 g NaCl) provided the best compromise to extract in one step all selected compounds. Hence, it was selected as the optimal extraction process for lettuce root and soil. Method performance characterization was subsequently conducted with protocol 15 for both matrices.

### MRM^HR^ vs. SWATH

For method development and comparison, only the MRM^HR^ mode was used by acquiring data in fragment scanning mode, whereas for performance characterization and to estimate the sensitivity, HRMS data were acquired using both MRM^HR^ and SWATH modes. The SCIEX-guided MRM^HR^ tool was used for all optimized detection parameters for each target analyte in positive and negative ionization modes (Table S3). For any further details regarding the acquisition methods, refer to SI or other publications (Montemurro et al. 2021; Montemurro et al. 2020). Qualitative and quantitative analyses were performed using SCIEX OS™ Software version 1.6 (Sciex, Redwood City, CA, USA). Two high-resolution ions were used for each compound, the most abundant product ion for the quantification and the precursor ion for the confirmation (Table S3) according to SANTE 11312/2021 (Pihlström et al. 2022). Each compound was confirmed by comparing the signal of two accurate mass ions (≤5 ppm). For isotopically label compounds only the accurate mass of molecular ion was used. For SWATH acquisition, high-confidence identification was based on unique fragment ions and their ion ratios as well as HR-MS/MS library searching using high-resolution spectral libraries supplied by SCIEX. Five main confidence criteria were used for positive identification determination, meeting the criteria of identification points, which were Mass Error, Fragment Mass Error, RT Error, Isotope Ratio, and Library Score (Gago-Ferrero et al. 2015; Sabater-Liesa et al. 2021). MRM^HR^ acquisition has excellent capability for quantitative analysis in complex matrix providing high accuracy, sensitivity, and selectivity using conventional triple-quadrupole mass spectrometry allowing more specific analysis of target compounds especially when the analytes of interest are poorly abundant/ionizable (Montemurro et al. 2020; Rajski et al. 2020). Nevertheless, SWATH acquisition is a more attractive data-independent acquisition (DIA) strategy that allows to identify and quantify every detectable compound in a sample in one single run, eliminating the risk of missing a relevant analyte. Every detectable analyte in the sample is fragmented giving a complete MS and MS/MS picture of every detectable in the sample which can be used either for quantification or a posteriori for retrospective analysis to identify new unidentified or unexpected compounds (Manjarrés-López et al. 2023). To improve the selectivity of the SWATH, the entire m/z mass detection range (99.5 to 950 m/z) in which ions are distributed along run time was divided into ten sequential *Q*_1_ variable windows. The variable windows were generated by injecting a respectively fortified root or soil sample with all target compounds (100 ng g^−1^) and calculating the number of precursor ions and considering their intensities as a weighting factor (Montemurro et al. 2020). The procedure of the variable windows according to the distribution of precursor ions within the retention time of the LC gradient for both positive and negative SWATH acquisition for root and soil, respectively, are reported in SI and Figures S2 and S3.

### Matrix effect evaluation

**Fig. 3:**
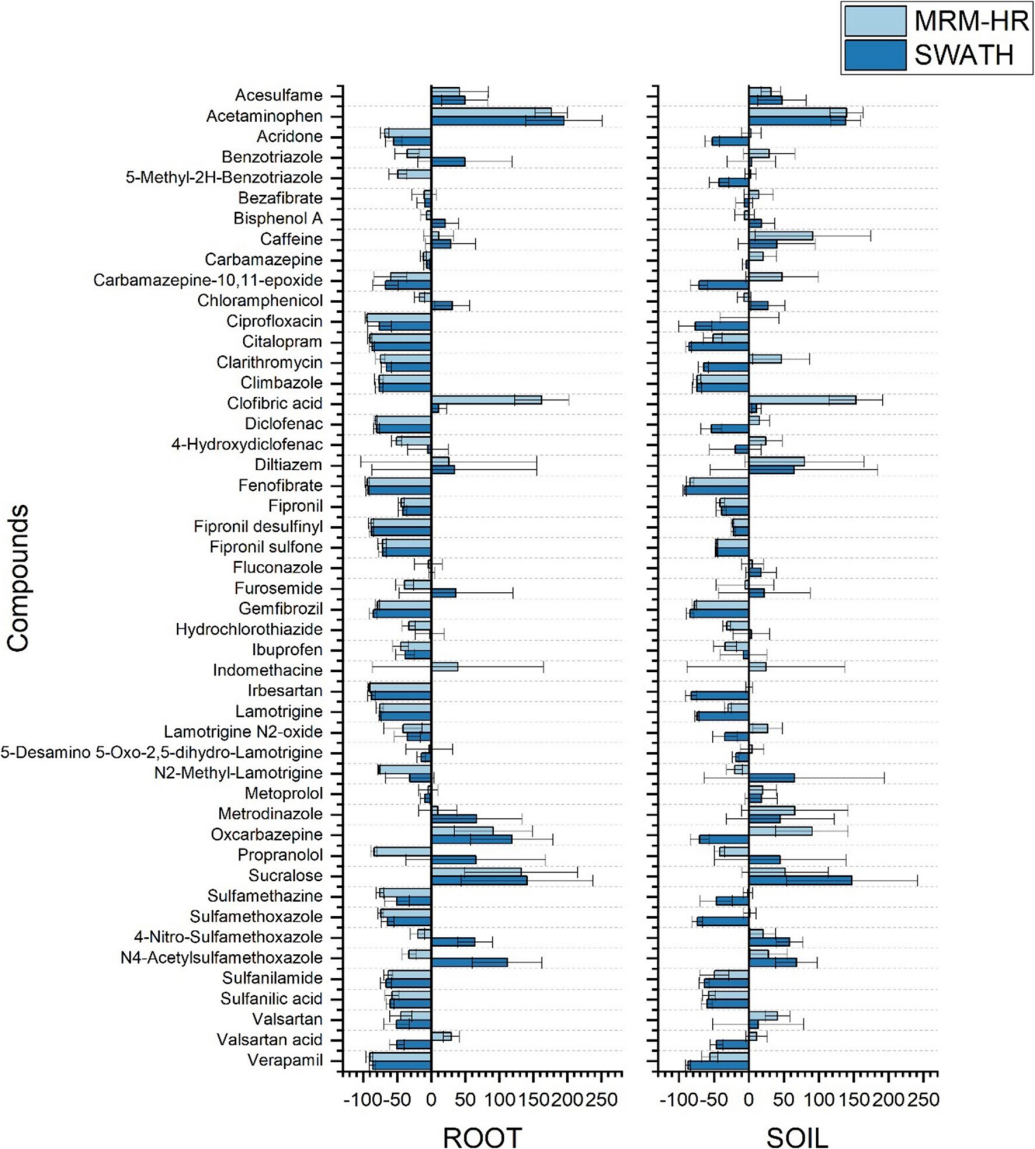
Mean of matrix effect (*ME%*) of five concentration levels for lettuce root and soil in MRMHR and SWATH acquisition modes

Lettuce root and soil are complex matrices, and co-extracted matrix components are often present in the final extract to be injected, thus decreasing or increasing the instrumental response of the target analytes. In this work, the *ME* was evaluated in MRM^HR^ and SWATH acquisition modes for the 5 concentration levels; detailed results are present in Table S6 and Table S7 for lettuce root and soil, respectively. The average *ME%* values for MRM^HR^ and SWATH acquisition are reported in Fig. 3, for lettuce root and soil, respectively. Moreover, 50% of the compounds showed acceptable *ME%* (from −40 to +40%) in both matrices. However, some compounds have shown an opposite performance between both acquisition modes such as propranolol (−83.8% in MRM^HR^ and 65.4% in SWATH), furosemide (−39.1% in MRM^HR^ and 36.4% in SWATH), benzotriazole (−35.6% in MRM^HR^ and 49.2% in SWATH), N4-acetyl-sulfamethoxazole (−32.7% in MRM^HR^ and 111.5% in SWATH), bisphenol A (−7.0% in MRM^HR^ and 20.5% in SWATH), and valsartan acid (29.3% in MRM^HR^ and −50.4% in SWATH) in the case of lettuce root. This opposite trend was more evident in the case of soil with a greater number of affected compounds such as acridone (3.4% in MRM^HR^ and −52.2% in SWATH), carbamazepine epoxide and oxcarbazepine (47.8% and 90.5% in MRM^HR^ and −71.5% and −70.5% in SWATH, respectively), diclofenac (15.3% in MRM^HR^ and −53.8% in SWATH), N2-methyl-lamotrigine (−20% in MRM^HR^ and −65.1% in SWATH), propranolol (−41.8% in MRM^HR^ and 44.7% in SWATH), and valsartan acid (11.4% in MRM^HR^ and −46.5% in SWATH). Acetaminophen showed the highest *ME%* in both acquisition modes 176.5% in MRM^HR^ and 195.2% in SWATH in the case of lettuce root, followed by clofibric acid (162.3%) in MRM^HR^ only, and sucralose (132.3% in MRM^HR^ and 140.7% in SWATH). Whereas, in soil the highest *ME%* was represented by clofibric acid (153.1%, in MRM^HR^ only), sucralose (147.4%, in SWATH only), and acetaminophen 139.7% in MRM^HR^ and 138.5% in SWATH. In general, the values obtained are very similar to those reported in our previous work under the same conditions and instrumentation, especially in the case of the roots (Labad et al. 2021; Montemurro et al. 2020). However, it should be noted that the cleanup step was excluded in the final selected protocol; hence, higher matrix effect was expected. In general, one way to reduce the matrix effect is to try to remove coelution components by performing appropriate sample preparation and cleanup procedures so that the quantity of matrix components introduced into the analytical system is lower, resulting in reduced matrix suppression (Cortese et al. 2020). However, this approach to minimize/compensate the matrix effect may present advantages or limitations such as the use of specific instruments or methods that are expensive, laborious, or time-consuming (Nasiri et al. 2021; Zhou et al. 2017). For example, it is well documented that in the case of QuEChERS, the use of dispersive PSA-based cleanup, although significantly reducing the quantity of co-extracts, would also tend to partially reduce recoveries especially of basic compounds (Anastassiades et al. 2003; Labad et al. 2021; Montemurro et al. 2020). Often, to reduce the ME, and therefore the quantity of matrix components introduced into the analytical system, it is sufficient to simply dilute the sample, provided that the sensitivity of the method is preserved (Cortese et al. 2020). This approach was effective in analyzing a range of pesticides in different vegetable matrices (Ferrer et al. 2011; Stahnke et al. 2012). The use of a more sensitive ion source also offers the possibility of reducing EM. Although ESI is the most used source, it is also the one most affected by EM. However, current commercially available ESI sources are designed to have different geometries, specific to reducing EM. Specifically, orthogonal geometry where the relative angle is 90° such as Sciex Turbo V™ usually improves sensitivity by preventing clogging of the orifice with non-volatile materials. In our case we decided to avoid the use of a cleanup or dilution step of the extract. In this second case, the dilution would have consequently also diluted the internal standards used as surrogates that we normally use in routine analyses. So, to overcome analytes response enhancement or reduction, quantification of real samples was performed using a matrix-matched calibration curve spiked with labelled standards.

### Method performance characterization

For method characterization, MRM^HR^ and SWATH acquisition modes were used. The optimized method was validated for both matrices in terms of accuracy, linearity, intra-day precision, method detection limit (*MDL*), and method quantification limit (*MQL*). Performance characterization parameters are summarized in Table S8 to Table S11 for lettuce root and soil.

#### Accuracy

**Fig. 4:**
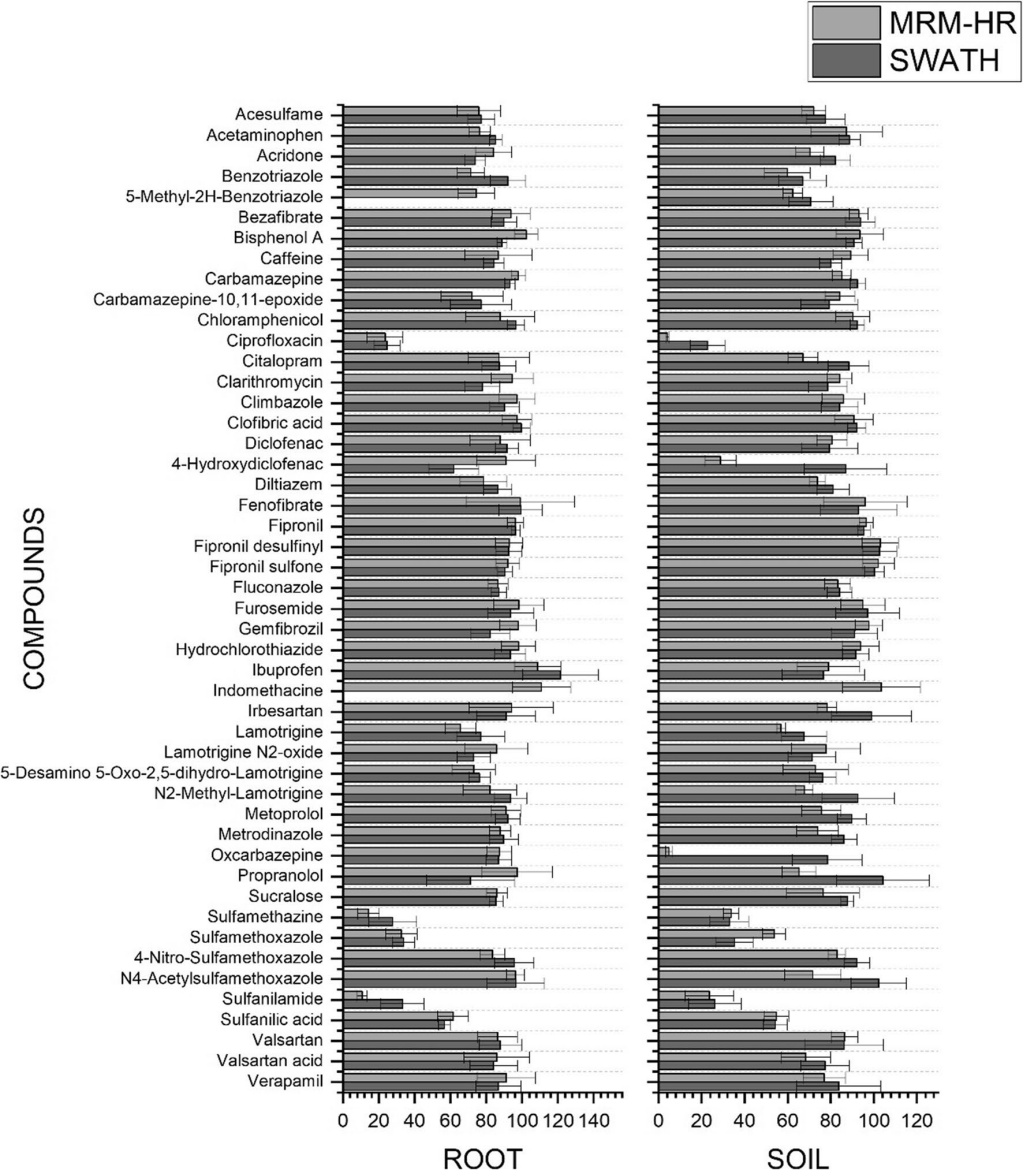
Mean of relative recoveries (RR%) of five concentration levels for lettuce root and soil in MRMHR and SWATH acquisition modes. Error bars show the range of relative standard deviation (RSD%)

Relative recoveries were obtained by the mean value of three replicates for each concentration level, in both acquisition modes (MRM^HR^ and SWATH). Figure 4 shows the recoveries expressed as mean calculated from the five studied levels for lettuce root and soil in MRM^HR^ (light gray) and SWATH (dark gray) acquisition modes. Detailed relative recoveries obtained at each concentration level are summarized in Table S8 and Table S9 for lettuce root and soil, respectively. The method exhibited good accuracy with relative recoveries between 80 and 120% for most of the studied compounds in both acquisition modes and for both matrices. However, some compounds were not extracted or presented relatively low recoveries from both matrices such as sulfanilamide (10.8% in MRM^HR^, it was recovered only at 50 and 200 μg L^−1^), sulfamethazine (14.3% in MRM^HR^ and 27.8% in SWATH), ciprofloxacin (23.6% in MRM^HR^ and 24.7% in SWATH), and 5-methyl-2H-benzotriazole and indomethacin (not recovered in SWATH) in root. Whereas, for soil the compounds not recovered or with low values were ciprofloxacin (4.1% in MRM^HR^ and 22.8% is SWATH), oxcarbazepine (4.9% in MRM^HR^), sulfanilamide (23.7% in MRM^HR^ and 26.2% in SWATH), and indomethacin (not recovered in SWATH). As for matrix effect, recoveries resulted very similar at our previous work (Montemurro et al. 2020). However, according to our knowledge, this study is the first to report the development of a lettuce root extraction method; the comparison of the results was limited to the soil matrix. Besides, few data are available in the literature for the compounds included in this study, dealing with soil matrix. Thereupon, comparable recoveries were obtained in previous studies for the compounds in common (Martínez-Piernas et al. 2018a; Salvia et al. 2012), though Salvia and coworkers recorded higher recoveries for sulfonamide (50%) (Salvia et al. 2012). However, this could be explained by the application of a cleanup step in their study, whereas we opted to avoid this step to keep the method simpler and promote optimal recovery of nearly all compounds of interest. Similar results in soil were also obtained by Kodešová et al. (2016) or by Golovko et al. (2016) analyzing more than 60 pharmaceutical residues including atenolol, carbamazepine, clarithromycin, metoprolol, and sulfamethoxazole in 13 different types of soil. Compared with Biel-Maeso et al. (2017) where the authors extract about 45 drugs from the soil of which 20 in common using pressurized hot water extraction (PHWE), at the lowest validation level (2 ng g^−1^), our method is 30–60% more effective.

#### Intra-day precision

Intra-day precision expressed by repeatability was calculated as relative standard deviation (RSD%) obtained from the relative recoveries described above, at each concentration level. Detailed results for MRM^HR^ and SWATH acquisition modes are summarized in Table S8 and Table S9 for lettuce root and soil, respectively. The bars in Fig. 4 show the mean RSD% for the 5 concentration levels studied for lettuce root and soil. Most of the compounds recorded high precision with RSD% below 20% for both acquisition modes and both matrices. However, some exceptional compounds with RSD% outside the acceptance criterion are always recorded, such as irbesartan (23.5% in MRM^HR^), fenofibrate (30.2% in MRM^HR^), and propranolol (24.6% in SWATH) for lettuce roots and fenofibrate (31.4% in MRM^HR^) and N_2_-methyl-lamotrigine (22.8% in SWATH).

#### Linearity

Linearity of the instrumental response was assessed using the matrix-matched calibration curve approach with a calibration curve constructed between 0.05 and 300 ng mL^−1^, corresponding to 0.5 to 3000 ng g^−1^ f.w. for lettuce root and 0.05 to 300 ng g^−1^ d.w. for soil, respectively, and considering at least 8 points. Results are summarized in Table S10 and Table S11 for lettuce root and soil, respectively. Analytes linearity was up to 1000, 2000, or 3000 ng g^−1^ in the case of lettuce and 100, 200, or 300 ng g^−1^ in the case of soil for most of the compounds covering several orders of magnitude for both acquisition modes although in SWATH the calibration curves tend to saturate very soon. This is the case of diltiazem (2.5–2000 and 0.5–100 ng g^−1^, respectively, for MRM^HR^ and SWATH) and metoprolol (5–3000 and 2.5–300 ng g^−1^, respectively, for MRM^HR^ and SWATH) in lettuce roots and diltiazem (0.05–100 and 0.05–10 ng g^−1^, respectively, for MRM^HR^ and SWATH) and verapamil (0.1–300 and 0.05–10 ng g^−1^, respectively, for MRM^HR^ and SWATH) in soil. Moreover, the linearity for 5-methyl-2H-benzotriazole, ibuprofen, and indomethacin for root and ibuprofen and indomethacin for soil could not be calculated in SWATH (Montemurro et al. 2020). However, fipronil and its two studied transformation products, namely, fipronil sulfone and fipronil desulfinyl, showed shorter linearity, up to 100 ng g^−1^ in lettuce root and 10 ng g^−1^ in soil in MRM^HR^ and SWATH for both acquisition modes, respectively (Table S10 and Table S11). On the other hand, all of the compounds exhibited *r*^2^ > 0.99, except for irbesartan (*r*^2^ = 0.96436) and furosemide (*r*^2^ = 0.98989) in MRM^HR^, and fipronil sulfone (*r*^2^ = 0.98777) and N2-methyl-lamotrigine (*r*^2^ = 0.97921) in SWATH for lettuce root. Whereas in the case of soil, the lowest values were recorded for caffeine (*r*^2^ = 0.98766 in MRM^HR^ and *r*^2^ = 0.98996 in SWATH) and sulfanilic acid (*r*^2^ = 0.98147 in MRM^HR^ and *r*^2^ = 0.97912 in SWATH).

#### MDLs and MQLs

Sensitivity of the methods was assessed through the estimation of *MDLs* and *MQLs* from the matrix-matched calibration curve using linear regression analysis (Montemurro et al. 2021; Montemurro et al. 2020). Results are summarized in Table S10 and Table S11 for lettuce root and soil, respectively. In general, *MDLs* and *MQLs* of lettuce root were higher than soil in both acquisition modes due to the complexity of the matrix. More in details, *MDLs* ranged from 0.01 to 0.30 ng g^−1^ in MRM^HR^ and from 0.01 to 0.77 ng g^−1^ in SWATH in the case of lettuce root, while *MDLs* ranged from 0.01 to 017 ng g^−1^ in MRM^HR^ and from 0.01 to 0.05 ng g^−1^ is SWATH for soil. Whereas, *MQLs* ranged from 0.03 to 0.92 ng g^−1^ in MRM^HR^ and from 0.03 to 0.82 ng g^−1^ in SWATH for lettuce root, and from 0.02 to 0.44 ng g^−1^ in MRM^HR^ and 0.02 to 0.14 ng g^−1^ in SWATH for soil. Though similar results were obtained with both acquisition modes, MRM^HR^ provided more consistent results for most of the compounds. In the case of roots, the sensitivity resulted comparable to the values obtained by Labad et al. (2021) where the same analytes were extracted from radish root with the QuEChERS method where the cleanup phase was deliberately skipped. *MDLs* ranged from 0.02 to 0.32 ng g^−1^ while *MQLs* ranged from 0.05 to 0.96 ng g^−1^, both acquired in MRM^HR^. In the case of soil, our results are comparable with Biel-Maeso et al. (2017) where the *MDLs* ranged from 0.01 to 0.83 ng g^−1^ and *MQLs* between 0.02 and 2.8 ng g^−1^, or slightly better when compared to Golovko et al. (2016) with *MQLs* 0.6 to 9.4 ng g^−1^.

### Application to real samples

**Fig. 5:**
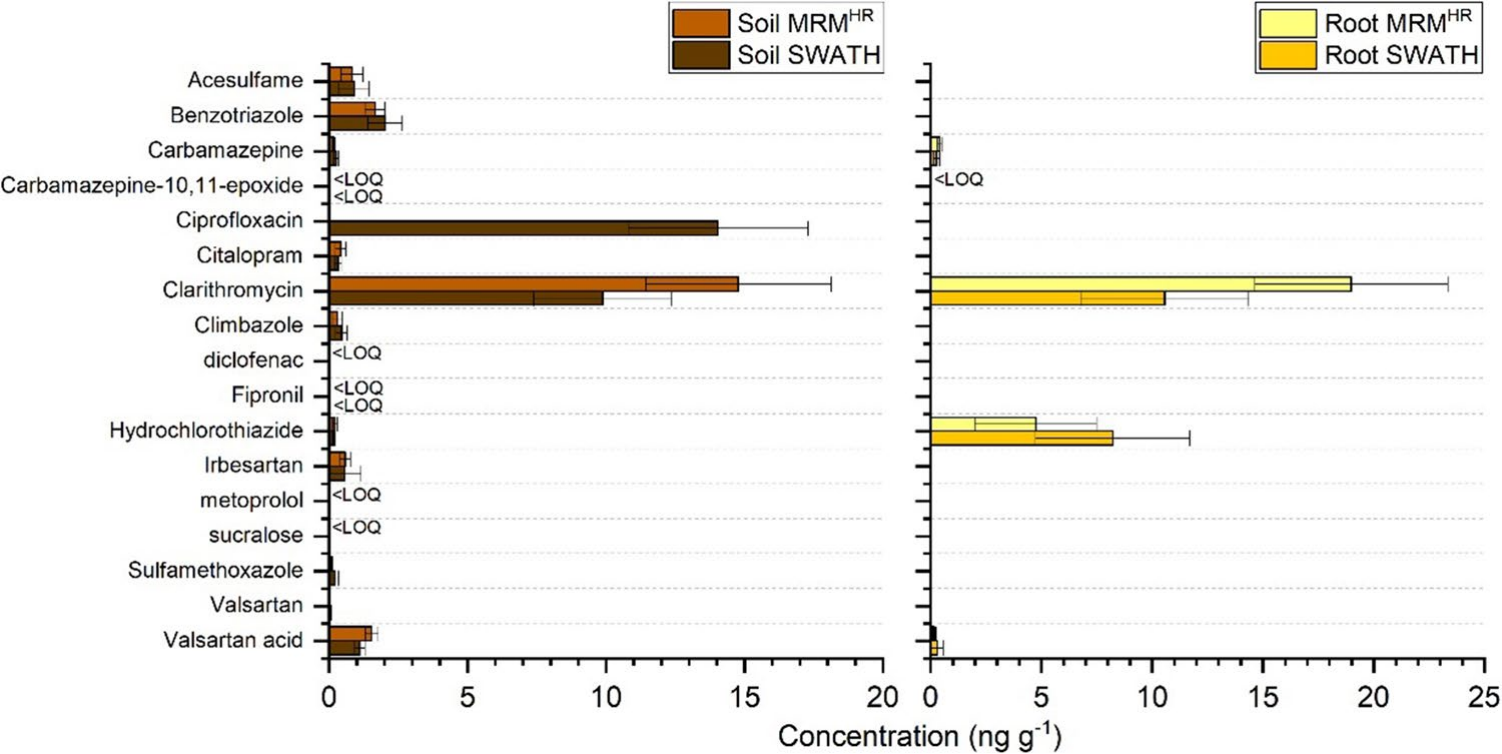
Quantification of wastewater-borne organic contaminants detected in soil and lettuce root samples (*n* = 8) irrigated with TWW under real greenhouse cultivation conditions

To evaluate the applicability of the methods, soil and root samples watered with TWW under greenhouse conditions were extracted according to the optimized procedures and quantify in MRM^HR^ and SWATH acquisition modes using the internal standard method where each analyte was quantified by using its corresponding labelled standard. The internal standards were added before the extraction phase (salting out) as surrogates. When added before the extraction step, internal standards are able to correct all random errors that occur both during the preparatory phase and during the instrumental analysis, thus improving the robustness, precision, and accuracy of the method. If a labeled compound was not available, an analog with a similar retention time or of the same class was used. According to the results (Fig. 5), 17 out of 48 tested compounds were detected in soil samples with concentrations ranging from <LOQ to 14.78 ng g^−1^. Only five compounds were detected in lettuce root with concentrations ranging from <LOQ to 18.99 ng g^−1^. Only clarithromycin and hydrochlorothiazide were detected at high concentrations (4.75 and 18.99 ng g^−1^, respectively). In a previous study, both compounds were detected at high concentrations in the deeper layer of the rhizosphere (Manasfi et al. 2021a). Most of them were already detected in lettuce leaves (Montemurro et al. 2020). In general, the low presence of contaminants in the roots could be justified by the intrinsic function of the root itself, i.e., absorbing and transporting water and solutes into the upper compartments. No significant differences were recorded between the two acquisition modes except for ciprofloxacin which was detected only through the use of SWATH in soil. The highest concentrations were always recorded for clarithromycin in root (18.99 ng g^−1^ in MRM^HR^) and soil (14.78 ng g^−1^ in MRM^HR^). Compared with the previous results in lettuce leaves, the low values reported for carbamazepine, carbamazepine epoxide, valsartan, and valsartan acid (Fig. 5) suggest that they are subject to leaf translocation. In instance for this, carbamazepine recorded 6.0, 0.41, and 0.18 ng g^−1^, whereas valsartan recorded 9.1, 0.18, and 1.54 ng g^−1^ in leaves, roots, and soil, respectively, in MRM^HR^. Comparable results were obtained with Martinez-Piernas and coworkers (Martínez-Piernas et al. 2018a), after analysis of agricultural soil irrigated with treated wastewater for a long period.

Bioconcentration factor (*BCF*) was estimated by dividing the concentration of detected contaminants in plant compartments (leaves + roots) to those in soil from respective location (Eq. 5) (McKone and Maddalena 2007):

5 $$\textrm{Bioconcentration}\ \textrm{factor}\ {\left(\textrm{BCF}\right)}_{d, ss}={C}_{\textrm{plant}}\left( mg\ g^{-1}\right)/{C}_{\textrm{dry}-\textrm{soil}}\left( mg\ g^{-1}\right)$$

where (*BCF*)_d,ss_ is the *BCF* for plant dry mass relative to dry soil (soil solids) concentration, *C*_plant_ is the concentration in dry plant tissue (*C*_leaves_ + *C*_roots_), and *C*_dry-soil_ is the total concentration of contaminants in dry soil. Results of the *BCF* for eight different analytes are tabulated in Table 1.

**Table 1:** Bioconcentration factor (*BCF*) of leaves and roots, relatively to soils for lettuce crops cultivated under controlled conditions

Compound	Ratio	*BCF*_leaves_^**^	Ratio	*BCF*_roots_	Ratio	*BCF*_TOTAL_
Carbamazepine	Leaves/soil	33.4 ± 13.7	Roots/soil	2.3 ± 0.2	Leaves + roots/soil	35.7 ± 13.6
Carbamazepine-10,11-epoxide^*^	Leaves/soil	905.6 ± 196.3	Roots/soil	10.0	Leaves + roots/soil	915.6 ± 196.3
Citalopram	Leaves/soil	47.4 ± 6.7	Roots/soil	n.a.	Leaves + roots/soil	47.4 ± 6.7
Climbazole	Leaves/Soil	9.2 ± 7.0	Roots/soil	n.a.	Leaves + roots/soil	9.2 ± 7.0
Hydrochlorothiazide	Leaves/soil	19.5 ± 11.8	Roots/soil	22.3 ± 1.8	Leaves + roots/soil	41.8 ± 11.3
Irbesartan	Leaves/soil	20.5 ± 16.9	Roots/soil	n.a.	Leaves + roots/soil	20.5 ± 16.9
Metoprolol^*^	Leaves/soil	58.7 ± 12.8	Roots/soil	n.a.	Leaves + roots/soil	58.7 ± 12.8
Valsartan acid	Leaves/soil	6.1 ± 1.0	Roots/soil	0.1	Leaves + roots/soil	6.1 ± 1.0

^*^Compounds with soil concentrations <LOQ. Half of the *MDLs* were taken into consideration for the calculations

^**^For the calculations in lettuce leaves, the values reported in Montemurro et al. (2020) were considered

The ratio of contaminants in plant tissues to the concentration in soil samples was calculated to estimate the bioconcentration factor. For simplicity, only the concentrations obtained in MRM^HR^ mode were considered. The higher the ratio, the greater the risks of contamination and potential adverse effects on soil invertebrates, animals, and humans. A *BCF* greater than 1 indicates greater contaminant uptake by roots and accumulation in leaves than in soil, while a *BCF* less than 1 indicates a greater concentration of contaminants in soil than those taken up by plants. Except for a few cases, most of the compounds do not show appreciable *BCF*_roots_ values due to lack of concentration data or being below detection limits. The obtained values clearly revealed the efficient tendency of carbamazepine and its metabolite carbamazepine-10,11-epoxide to translocate and bioaccumulate in the edible parts of lettuce. Indeed, the *BCF* value obtained for carbamazepine is totally in agreement with previous studies in which it was found that the highest accumulation was found in the leaves of the plants rather than in the roots (Kodešová et al. 2019; Labad et al. 2021; Manasfi et al. 2021a; Menacherry et al. 2023; Montemurro et al. 2017; Mordechay et al. 2022). Furthermore, its metabolite, carbamazepine-10,11-epoxide, presented <LOQ values in soil and roots which together with a very high *BCF* indicate the ability of this metabolite to translocate completely into the aerial parts of the plant with potential toxic effects at the leaf level (Carter et al. 2014; Malchi et al. 2014; Riemenschneider et al. 2017). Furthermore, similar accumulation trends are shown in Table 1, with a high *BCF* value; it was also observed in the case of citalopram, metoprolol, and hydrochlorothiazide. In the latter case, 50% of the *BCF* can be attributed to the roots.

## Conclusions

This work presents a sensitive, robust, simple, and low-cost method for the simultaneous extraction of a wide range of wastewater borne from complex environmental matrices such as soil and lettuce root, based on QuEChERS method. According to our knowledge, it is the first time that a work was dedicated to optimize and validate a method for lettuce root matrix. The use of EDTA-Mcllvaine buffer in combination with QuEChERS can therefore be considered a reliable alternative for the extraction of a wide range of organic substances in solid matrices in one step. The developed method was characterized in terms of recovery, linearity, intra-day precision, *MDLs*, *MQLs*, and matrix effect in two different acquisition modes, MRM^HR^ and SWATH, in order to compare their method performance. HRMS as MRM^HR^ or SWATH acquisition offers the advantage of accurate and reliable results in a short analysis time while resulting in a reduction of steps (cleanup) with a low susceptibility to errors. Though similar results were obtained with both acquisition modes, MRM^HR^ provided more consistent results for most of the compounds. Additionally, this method was successfully applied to real samples cultivated under greenhouse conditions and 5 and 17 pollutants were detected in lettuce root and soil, respectively. Furthermore, irrigation of the soil-crop system with TWW contaminated with trace organic chemicals can lead to their uptake and accumulation in the different parts of the lettuce (leaves and roots), as well as their accumulation in the soil, which entails potential risks for the human health and the environment.

### Supplementary information

Supplementary file 1 (DOCX 2.34 MB)

## Data Availability

All data generated or analyzed during this study are included in this published article.\
