# Supplementary material for: Evaluation of different QuEChERS-based methods for the extraction of 48 wastewater-derived organic contaminants from soil and lettuce root using high-resolution LC-QTOF with MRMHR and SWATH acquisition modes
Source: Environ Sci Pollut Res Int. 2024 Feb 19;31(13):20258–76. doi: 10.1007/s11356-024-32423-w (PMC10927905; doi:10.1007/s11356-024-32423-w)
Supplement: Supplementary file 1 — (DOCX 2.34 MB) [file 11356_2024_32423_MOESM1_ESM.docx]

**Supplementary Information**

**Evaluation of different modified QuEChERS-based methods for the extraction of 48 wastewater-derived organic contaminants from soil and lettuce root using high resolution LC-QTOF with MRM^HR^ and SWATH acquisition modes**

Nicola Montemurro^1*^, Rayana Manasfi^1,2^, Serge Chiron^2^, Sandra Perez^1^

^1^Environmental and Water Chemistry for Human Health (ONHEALTH), Institute of Environmental Assessment and Water Research (IDAEA-CSIC), c/Jordi Girona 18-26, 08034 Barcelona (Spain)

^2^HydroSciences Montpellier (HSM), University of Montpellier Building 39 - CC57 300 Avenue du Professeur Emile Jeanbrau, 34090 Montpellier (France)

***Corresponding author:**

Nicola Montemurro

IDAEA-CSIC

ONHEALTH Research Group

Jordi Girona 18-26

Barcelona 08034, Spain

E-mail: nmoqam@cid.csic.es

Phone: +34-93 255 7683

Fax: +34-93 204 5904

*Table of Contents*

[Experimental section: 3](#_Toc157450323)

[EDTA Mcllvaine buffer (pH =4.0) preparation: 3](#_Toc157450324)

[Table S1. physical and chemical properties of the target compounds. 3](#_Toc157450325)

[Table S2. Details of the tested protocols for lettuce root and soil matrices (protocols 17 to 22 are for soil matrix only). 5](#_Toc157450326)

[Table S3. MRM^HR^ optimized detection parameters in positive and negative ionization modes 6](#_Toc157450327)

[Figure S1. Comparison of Control roots (A) and Exposed roots (B) after the harvest. 8](#_Toc157450328)

[LC-MS/MS analysis: 9](#_Toc157450329)

[Figure S2. Variable Q1 Window Widths for SWATH Acquisition in positive (A) and negative (B) ionization for lettuce root 10](#_Toc157450330)

[Figure S3. Variable Q1 Window Widths for SWATH Acquisition in positive (A) and negative (B) ionization for soil 11](#_Toc157450331)

[Results and discussion: 12](#_Toc157450332)

[Figure S4. Statistical comparison between the different protocols and matrices, using a loss function to optimize the protocol selection. 12](#_Toc157450333)

[Figure S5. Logit-penalized average recovery rates. 12](#_Toc157450334)

[Table S4. List of compounds with recoveries > 60%, 40-60%, 20-40%, 0-20% and ND (not detected) for lettuce root matrix 13](#_Toc157450335)

[Table S5. List of compounds with recoveries > 60%, 40-60%, 20-40%, 0-20% and ND (not detected) for soil matrix 16](#_Toc157450336)

[Table S6. Comparison of Matrix effect (%) for target analytes in MRMHR and SWATH acquisition modes in lettuce root matrix 24](#_Toc157450337)

[Table S7. Comparison of Matrix effect (%) for target analytes in MRM^HR^ and SWATH acquisition modes in soil matrix 26](#_Toc157450338)

[Table S8. Relative recoveries and intraday precision (RSD%) at 5 concentrations (2, 5, 10, 50, 200 µg L^-1^) with MRM^HR^ and SWATH acquisition modes for lettuce root matrix 28](#_Toc157450339)

[Table S9. Relative recoveries and intraday precision (RSD%) at 5 concentrations (2, 5, 10, 50, 200 µg L^-1^) with MRM^HR^ and SWATH acquisition modes for soil matrix 30](#_Toc157450340)

[Table S10. Linearity of the instrumental response (Linearity), MDL and MQL with MRM^HR^ and SWATH acquisition modes for lettuce root matrix 32](#_Toc157450341)

[Table S11. Linearity of the instrumental response (Linearity), MDL and MQL with MRM^HR^ and SWATH acquisition modes for soil matrix 34](#_Toc157450342)

[Table S12. Concentration of organic contaminants in the wastewater used for irrigation calculated in SWATH acquisition mode 36](#_Toc157450343)

# Experimental section:

## EDTA Mcllvaine buffer (pH =4.0) preparation:

1.5 g of disodium hydrogen phosphate dehydrate, 1.3 g of citric acid monohydrate, and 0.372 g EDTA were dissolved in 100 mL HPLC water.

## Table S1. physical and chemical properties of the target compounds.

| Compound | CAS No. | Molecular formula | Molecular weight | Water solubility | Log Kow | pka | Chemspider ID | Family group |
| --- | --- | --- | --- | --- | --- | --- | --- | --- |
| Acesulfame | 33665-90-6 | C_4_H_5_NO_4_S | 163.15 | 9.10E+05 | -1.33 | 3.0 | 33607 | Artificial  sweetener |
| Acetaminophen | 103-90-2 | C_8_H_9_NO_2_ | 151.16 | 3.03E+04 | 0.46 | 7.0 | 1906 | analgesic |
| Acridone | 578-95-0 | C_13_H_9_NO | 195.22 | 1.31E+02 | 1.69 | -0.3 | 10188539 | TP of carbamezapine |
| Benzotriazole | 95-14-7 | C_6_H_5_N_3_ | 119.12 | 1.10E+04 | 1.44 | 8.4 | 6950 | Drug precursor |
| 5-Methyl-2H-Benzotriazole | 136-85-6 | C_7_H_7_N_3_ | 133.15 | 3.07E+03 | 1.71 | 8.7 | 8381 | Industrial product |
| Bezafibrate | 41859-67-0 | C_19_H_20_ClNO_4_ | 361.82 | 1.22E+00 | 4.25 | 3.8 | 35728 | lipid regulators |
| Bisphenol A | 80-05-7 | C_15_H_16_O_2_ | 228.29 | 1.73E+02 | 3.32 | 9.6 | 6371 | plasticizer |
| Caffeine | 58-08-2 | C_8_H_10_N_4_O_2_ | 194.19 | 2.16E+04 | -0.07 | 14.0 | 2424 | stimulants |
| Carbamazepine | 298-46-4 | C_15_H_12_N_2_O | 236.27 | 1.77E+01 | 2.45 | 7.0 | 2457 | psychiatric drug |
| Carbamazepine-10,11-epoxide | 36507-30-9 | C_15_H_12_N_2_O_2_ | 252.27 | 2.77E+02 | 0.95 | -3.7; 15.96 | 2458 | Metabolite carbamezapine |
| Chloramphenicol | 56-75-7 | C_11_H_12_Cl_2_N_2_O_5_ | 323.13 | 2.50E+03 | 1.14 | 5.5 | 5744 | antibiotic |
| Ciprofloxacin | 85721-33-1 | C_17_H_18_FN_3_O_3_ | 331.34 | 1.15E+04 | 0.28 | 6.1 | 2662 | fluoroquinoles |
| Citalopram | 59729-33-8 | C_20_H_21_FN_2_O | 324.39 | 3.11E+01 | 3.74 | 9.8 | 2669 | psychiatric drug and stimulant |
| Clarithromycin | 81103-11-9 | C_38_H_69_NO_13_ | 747.95 | 1.69E+00 | 3.16 | 9.0 | 10342604 | macrolides |
| Climbazole | 38083-17-9 | C_15_H_17_ClN_2_O_2_ | 292.76 | 8.28E+00 | 3.76 | 5.6 | 34752 | antifungal agent |
| Clofibric acid | 882-09-7 | C_10_H_11_ClO_3_ | 214.65 | 5.83E+02 | 2.57 | 3.2 | 2695 | lipid regulator |
| Diclofenac | 15307-86-5 | C_14_H_11_Cl_2_NO_2_ | 296.15 | 4.52E+00 | 4.51 | 4.2 | 2925 | Analgesic |
| 4-Hydroxydiclofenac | 64118-84-9 | C_14_H_11_Cl_2_NO_3_ | 312.15 | 1.74E+04 | 3.70 | 3.8 | 104192 | Analgesic |
| Diltiazem | 42399-41-7 | C_22_H_26_N_2_O_4_S | 414.52 | 1.23E+01 | 2.70 | 8.1 | 35850 | anti-hypertensive |
| Fenofibrate | 49562-28-9 | C_20_H_21_ClO_4_ | 360.83 | 1.96E-01 | 5.19 | -4.9 | 3222 | lipid regulator |
| Fipronil | 120068-37-3 | C_12_H_4_Cl_2_F_6_N_4_OS | 437.15 | 4.00E+00 | 1.90 | 7.0 | 3235 | insecticide |
| Fipronil desulfinyl | 205650-65-3 | C_12_H_4_Cl_2_F_6_N_4_ | 389.08 | 4.86E-01 | 4.22 | 7.0 | 11542895 | insecticide |
| Fipronil sulfone | 120068-36-2 | C_12_H_4_Cl_2_F_6_N_4_O_2_S | 453.15 | 1.30E-01 | 4.42 | 7.0 | 2336427 | insecticide |
| Fluconazole | 86386-73-4 | C_13_H_12_F_2_N_6_O | 306.27 | 3.36E+02 | 0.25 | 1.8 | 3248 | antifungal agent |
| Furosemide | 54-31-9 | C_12_H_11_ClN_2_O_5_S | 330.74 | 7.31E+01 | 2.03 | 3.9 | 3322 | diuretic |
| Gemfibrozil | 25812-30-0 | C_15_H_22_O_3_ | 250.33 | 4.96E+00 | 4.77 | 4.5 | 3345 | lipid regulator |
| Hydrochlorothiazide | 58-93-5 | C_7_H_8_ClN_3_O_4_S_2_ | 297.74 | 7.22E+02 | -0.07 | 7.9 | 3513 | diuretics |
| Ibuprofen | 15687-27-1 | C_13_H_18_O_2_ | 206.28 | 2.10E+01 | 3.97 | 5.3 | 3544 | analgesics |
| Indomethacine | 53-86-1 | C_19_H_16_ClNO_4_ | 357.79 | 9.37E-01 | 4.27 | 4.5 | 3584 | antiinflammatory |
| Irbesartan | 138402-11-6 | C_25_H_28_N_6_O | 428.53 | 5.99E-02 | 5.31 | 4.1; 4.3 | 3618 | anti-hypertensive |
| Lamotrigine | 84057-84-1 | C_9_H_7_Cl_2_N_5_ | 256.09 | 3.13E+03 | 0.99 | 8.5 | 3741 | psychiatric drug and stimulant |
| Lamotrigine N2-oxide | 136565-76-9 | C_9_H_7_Cl_2_N_5_O | 272.09 | N/A | 1.93 | 5.7 | 29790380 | Metabolite of lamotrigien |
| 5-Desamino 5-Oxo-2,5-dihydro-Lamotrigine | 252186-78-0 | C_9_H_6_Cl_2_N_4_O | 257.08 | 1.01E+02 | 2.73 | 8.6 | 13519082 | Metabolite of lamotrigine |
| N2-Methyl-Lamotrigine | 1152091-68-3 | C_10_H_10_Cl_2_N_5_ | 271.13 | N/A | N/A | N/A | 52083157 | Metabolite lamotrigine |
| Metoprolol | 51384-51-1 | C_15_H_25_NO_3_ | 267.36 | 4.78E+03 | 2.88 | 9.7 | 138781 | beta-blocker |
| Metrodinazole | 443-48-1 | C_6_H_9_N_3_O_3_ | 171.15 | 7.89E+04 | -0.6 | 7.0 | 62916 | Antifungal agent |
| Oxcarbazepine | 28721-07-5 | C_15_H_12_N_2_O_2_ | 252.27 | 2.03E+02 | 1.11 | -4.3; 13.18 | 31608 | psychiatric drugs and stimulants |
| Propranolol | 525-66-6 | C_16_H_21_NO_2_ | 259.34 | 2.28E+02 | 3.48 | 9.4 | 4777 | beta-blockers |
| Sucralose | 56038-13-2 | C_12_H_19_Cl_3_O_8_ | 397.63 | 2.28E+04 | -1.00 | 7.0 | 64561 | Artificial  sweetener |
| Sulfamethazine | 57-68-1 | C_12_H_14_N_4_O_2_S | 278.33 | 1.50E+03 | 0.89 | 2.7; 7.7 | 5136 | sulfonamide antibiotics |
| Sulfamethoxazole | 723-46-6 | C_10_H_11_N_3_O_3_S | 253.28 | 6.10E+02 | 0.89 | 1.6; 5.7 | 5138 | sulfonamide antibiotics |
| 4-Nitro-Sulfamethoxazole | 29699-89-6 | C_10_H_9_N_3_O_5_S | 283.26 | 5.69E+02 | 1.22 | 5.65 | 626159 | TP of sulfamethoxazole |
| N4-Acetylsulfamethoxazole | 21312-10-7 | C_12_H_13_N_3_O_4_S | 295.31 | 1.22E+03 | 1.21 | 5.88 | 58771 | Metabolite sulfamethoxazole |
| Sulfanilamide | 63-74-1 | C_6_H_8_N_2_O_2_S | 172.21 | 7.50E+03 | -0.62 | 6.1 | 5142 | antibiotic |
| Sulfanilic acid | 121-57-3 | C_6_H_7_NO_3_S | 173.19 | 1.08E+04 | -2.16 | 5.3 | 8166 | antibiotic |
| Valsartan | 137862-53-4 | C_24_H_29_N_5_O_3_ | 435.52 | 1.41E+00 | 3.65 | 3.9; 48 | 54833 | anti-hypertensive |
| Valsartan acid | 164265-78-5 | C_14_H_10_N_4_O_2_ | 266.25 | 1.406 | 4.0 | 5.0 | N/A | TP of Valsarta |
| Verapamil | 52-53-9 | C_27_H_38_N_2_O_4_ | 454.60 | 4.47E+00 | 3.79 | 8.9 | 2425 | anti-hypertensive |

Physicochemical properties, water solubility (in mg/L), polarity (log Kow), and pka of selected compounds of study classified by their uses were estimated and obtained from the platforms Chemspider (www.chemspider.com) and PubChem (https://pubchem.ncbi.nlm.nih.gov) or estimated from log Kow, by EPISuite platform, extracted from Chemspider database.

## Table S2. Details of the tested protocols for lettuce root and soil matrices (protocols 17 to 22 are for soil matrix only).

|  | Hydration solution* | | | | 1 minute vortex and 1h rest – addition of the standard mix and 1h rest – salts and extraction solvent addition | Extraction solvent | | Sonication | Type of salts | | | | 1 minute hand shake – 1 minute vortex – centrifugation at 4 °C, 4000 rpm for 10 minutes | Cleanup | | | Injection |
| --- | --- | --- | --- | --- | --- | --- | --- | --- | --- | --- | --- | --- | --- | --- | --- | --- | --- |
|  | **HPLC water** | **NH_4_HCO_2_**  **0.35 mol L^-1^** | **NH_4_CH_3_CO_2_**  **0.28 mol L^-1^** | **EDTA buffer**  **(pH 4)** |  | **ACN** | **ACN + 0.5% FA** |  | **OR-a** | **CEN** | **OR-b** | **OR-Na**  **(homemade)** |  | **dSPE** | **dSPE + 1% FA** | **no cleanup** |  |
| Protocol 1 | X |  |  |  |  | X |  |  | X |  |  |  |  |  |  | X | Centrifugation at 4 °C, 4000 rpm for 5 min - evaporation of 1 ml of the final extract under gentel nitrogen gaz – reconstitution with water:metanol (90:10) - injection |
| Protocol 2 | X |  |  |  |  | X |  |  | X |  |  |  |  | X |  |  |  |
| Protocol 3 | X |  |  |  |  | X |  |  | X |  |  |  |  |  | X |  |  |
| Protocol 4 | X |  |  |  |  |  | X |  | X |  |  |  |  |  |  | X |  |
| Protocol 5 | X |  |  |  |  |  | X |  | X |  |  |  |  | X |  |  |  |
| Protocol 6 | X |  |  |  |  |  | X |  | X |  |  |  |  |  | X |  |  |
| Protocol 7 | X |  |  |  |  | X |  |  |  | X |  |  |  |  |  | X |  |
| Protocol 8 | X |  |  |  |  | X |  |  |  | X |  |  |  | X |  |  |  |
| Protocol 9 | X |  |  |  |  | X |  |  |  | X |  |  |  |  | X |  |  |
| Protocol 10 | X |  |  |  |  |  | X |  |  | X |  |  |  |  |  | X |  |
| Protocol 11 | X |  |  |  |  |  | X |  |  | X |  |  |  | X |  |  |  |
| Protocol 12 | X |  |  |  |  |  | X |  |  | X |  |  |  |  | X |  |  |
| Protocol 13 |  | X |  |  |  | X |  |  | X |  |  |  |  |  |  | X |  |
| Protocol 14 |  |  | X |  |  | X |  |  | X |  |  |  |  |  |  | X |  |
| Protocol 15 |  |  |  | X |  | X |  |  | X |  |  |  |  |  |  | X |  |
| Protocol 16 |  |  |  | X |  | X |  | X | X |  |  |  |  |  |  | X |  |
| Protocol 17 |  | X  (8 mL) |  | X |  | X |  | X | X |  |  |  |  |  |  | X |  |
| Protocol 18 |  |  | X  (8 mL) | X |  | X |  | X | X |  |  |  |  |  |  | X |  |
| Protocol 19 | X  (5 mL) |  |  |  |  | X |  |  |  |  | X |  |  |  |  | X |  |
| Protocol 20 |  |  | X  (5 mL) |  |  | X |  |  |  |  | X |  |  |  |  | X |  |
| Protocol 21 |  |  |  | X  (5 mL) |  | X |  |  |  |  | X |  |  |  |  | X |  |
| Protocol 22 | X  (5 mL) |  |  |  |  | X |  |  |  |  |  | X |  |  |  | X |  |

*Hydration solution volume is 9 and 8 mL for lettuce root and soil respectively, unless it is indicated elsewhere.

## Table S3. MRM^HR^ optimized detection parameters in positive and negative ionization modes

| **Analyte** | **Chemical formula** | **Adduct/Charge** | **Precursor mass (m/z)** | **Fragment mass (m/z)** | **Declustering potential (V)** | **Collision energy (V)** | **Retention Time (min)** | **IS name** |
| --- | --- | --- | --- | --- | --- | --- | --- | --- |
| **Acesulfame** | C_4_H_5_NO_4_S | [M-H]- | 161.9866 | 82.0297 | -25 | -15 | 1.18 | Acesulfame-d4 |
| **Acetaminophen** | C_8_H_9_NO_2_ | [M-H]- | 150.0560 | 107.0379 | -95 | -20 | 2.01 | Acetaminophen-d4 |
| **Acridone** | C_13_H_9_NO | [M+H]+ | 196.0757 | 167.0644 | 150 | 40 | 5.82 | Carbamazepine-d10 |
| **Benzotriazole** | C_6_H_5_N_3_ | [M-H]- | 118.0411 | 50.0037 | -95 | -35 | 3.23 | Benzotriazole-d4 |
| **5-Methyl-2H-Benzotriazole** | C_7_H_7_N_3_ | [M+H]+ | 134.0713 | 77.0344 | 90 | 35 | 4.41 | Benzotriazole-d4 |
| **Bezafibrate** | C_19_H_20_ClNO_4_ | [M-H]- | 360.1008 | 274.0648 | -100 | -20 | 5.38 | Bezafibrate-d4 |
| **Bisphenol A** | C_15_H_16_O_2_ | [M-H]- | 227.1077 | 212.0845 | -165 | -20 | 7.39 | Bisphenol A-d8 |
| **Caffeine** | C_8_H_10_N_4_O_2_ | [M+H]+ | 195.0877 | 138.0685 | 110 | 25 | 2.82 | Caffeine-^13^C3 |
| **Carbamazepine** | C_15_H_12_N_2_O | [M+H]+ | 237.1022 | 194.0981 | 115 | 25 | 6.42 | Carbamazepine-d10 |
| **Carbamazepine-10,11-epoxide** | C_15_H_12_N_2_O_2_ | [M+H]+ | 253.0972 | 180.0736 | 60 | 55 | 5.32 | Carbamazepine-d10 |
| **Chloramphenicol** | C_11_H_12_Cl_2_N_2_O_5_ | [M-H]- | 321.0050 | 152.0352 | -95 | -20 | 5.18 | Bisphenol A-d8 |
| **Ciprofloxacin** | C_17_H_18_FN_3_O_3_ | [M+H]+ | 332.1405 | 314.1292 | 155 | 35 | 3.54 | Ciprofloxacin-d8 |
| **Citalopram** | C_20_H_21_FN_2_O | [M+H]+ | 325.1711 | 109.0396 | 30 | 35 | 6.22 | Citalopram-d6 |
| **Clarithromycin** | C_38_H_69_NO_13_ | [M+H]+ | 748.4842 | 158.116 | 60 | 40 | 7.22 | Ciprofloxacin-d8 |
| **Climbazole** | C_15_H_17_ClN_2_O_2_ | [M-H]- | 291.0906 | 67.0304 | -110 | -20 | 8.39 | Climbazole-d4 |
| **Clofibric acid** | C_10_H_11_ClO_3_ | [M-H]- | 213.0324 | 126.9961 | -80 | -20 | 4.07 | Benzotriazole-d4 |
| **Diclofenac** | C_14_H_11_Cl_2_NO_2_ | [M-H]- | 294.0094 | 250.0205 | -65 | -15 | 6.95 | Diclofenac-^13^C6 |
| **4-Hydroxydiclofenac** | C_14_H_11_Cl2NO_3_ | [M + H]+ | 312.0189 | 230.0277 | 65 | 45 | 7.54 | Diclofenac-^13^C_6_ |
| **Diltiazem** | C_22_H_26_N_2_O_4_S | [M+H]+ | 415.1686 | 178.0261 | 35 | 35 | 6.37 | Carbamazepine-d10 |
| **Fenofibrate** | C_20_H_21_ClO_4_ | [M+H]+ | 361.1201 | 139.0002 | 100 | 35 | 9.89 | Fenofibrate-d6 |
| **Fipronil** | C_12_H_4_Cl_2_F_6_N_4_OS | [M-H]- | 434.9314 | 329.96 | -25 | -20 | 8.92 | Climbazole-d4 |
| **Fipronil desulfinyl** | C_12_H_4_Cl_2_F_6_N_4_ | [M-H]- | 386.96444 | 350.9889 | -100 | -20 | 9.05 | Climbazole-d4 |
| **Fipronil sulfone** | C_12_H_4_Cl_2_F_6_N_4_O_2_S | [M-H]- | 450.9263 | 414.9508 | -25 | -20 | 9.22 | Climbazole-d4 |
| **Fluconazole** | C_13_H_12_F_2_N_6_O | [M+H]+ | 307.1113 | 238.0834 | 105 | 20 | 4.02 | Fluconazole-^13^C3 |
| **Furosemide** | C_12_H_11_ClN_2_O_5_S | [M-H]- | 329.0004 | 285.0115 | -110 | -20 | 4.42 | Furosemide-d5 |
| **Gemfibrozil** | C_15_H_22_O_3_ | [M-H]- | 249.1496 | 121.0671 | -95 | -20 | 8.78 | Gemfibrozil-d6 |
| **Hydrochlorothiazide** | C_7_H_8_ClN_3_O_4_S_2_ | [M-H]- | 295.9572 | 268.9476 | -140 | -25 | 2.77 | Hydrochlorothiazide-d2 |
| **Ibuprofen** | C_13_H_18_O_2_ | [M-H]- | 205.1234 | 161.1341 | -25 | -10 | 7.68 | Ibuprofen-d3 |
| **Indomethacine** | C1_9_H_16_ClNO_4_ | [M-H]- | 356.0695 | 312.0798 | -100 | -10 | 7.24 | Indomethacin-d4 |
| **Irbesartan** | C_25_H_28_N_6_O | [M-H]- | 427.2247 | 193.1347 | -150 | -35 | 6.49 | Irbesartan-d6 |
| **Lamotrigine** | C_9_H_7_Cl_2_N_5_ | [M+H]+ | 256.0151 | 210.9719 | 145 | 35 | 4.02 | Lamotrigine-^13^C3 |
| **Lamotrigine N2-oxide** | C_9_H_7_Cl_2_N_5_O | [M+H]+ | 272.0106 | 242.0051 | 120 | 25 | 3.91 | Lamotrigine-^13^C3 |
| **5-Desamino 5-Oxo-2,5-dihydro-Lamotrigine** | C_9_H_6_Cl_2_N_4_O | [M+H]+ | 256.9991 | 228.9985 | 160 | 25 | 4.64 | Lamotrigine-^13^C3 |
| **N2-Methyl-Lamotrigine** | C_10_H_9_Cl_2_N_5_ | [M+H]+ | 270.0308 | 57.039 | 160 | 40 | 4.62 | Lamotrigine-^13^C3 |
| **Metoprolol** | C_15_H_25_NO_3_ | [M+H]+ | 268.1907 | 116.1077 | 120 | 25 | 4.17 | Metoprolol-d7 |
| **Metrodinazole** | C_6_H_9_N_3_O_3_ | [M+H]+ | 172.0717 | 128.0429 | 85 | 15 | 2.16 | Metronidazole-d4 |
| **Oxcarbazepine** | C_15_H_12_N_2_O_2_ | [M+H]+ | 253.0972 | 180.0726 | 115 | 40 | 5.6 | Carbamazepine D10 |
| **Propranolol** | C_16_H_21_NO_2_ | [M+H]+ | 260.1645 | 116.109 | 100 | 25 | 5.64 | Metoprolol_D7 |
| **Sucralose** | C_12_H_19_Cl_3_O_8_ | [M-H]- | 395.0073 | 359.0283 | -130 | -15 | 3.35 | Sucralose d6 |
| **Sulfamethazine** | C_12_H_14_N_4_O_2_S | [M+H]+ | 279.091 | 124.0937 | 50 | 30 | 3.62 | Sulfamethazine d4 |
| **Sulfamethoxazole** | C_10_H_11_N_3_O_3_S | [M-H]- | 252.0448 | 156.0131 | -35 | -20 | 4.16 | Sulfamethoxazole-d4 |
| **4-Nitro-Sulfamethoxazole** | C_10_H_9_N_3_O_5_S | [M+H]+ | 284.0336 | 189.0202 | 130 | 30 | 6.86 | Sulfamethoxazole-d4 |
| **N4-Acetylsulfamethoxazole** | C_12_H_13_N_3_O_4_S | [M-H]- | 294.0554 | 198.0233 | -110 | -20 | 3.72 | Sulfamethoxazole-d4 |
| **Sulfanilamide** | C_6_H_8_N_2_O_2_S | [M-H]- | 172.0069 | 79.9591 | -110 | -45 | 1.28 | Sulfamethoxazole-d4 |
| **Sulfanilic acid** | C_6_H_7_NO_3_S | [M-H]- | 171.0229 | 78.9205 | -100 | -40 | 0.47 | Sulfamethoxazole-d4 |
| **Valsartan** | C_24_H_29_N_5_O_3_ | [M-H]- | 434.2197 | 179.0863 | -135 | -30 | 5.37 | Valsartan-d3 |
| **Valsartan acid** | C_14_H_10_N_4_O_2_ | [M+H]+ | 267.0877 | 206.0519 | 110 | 20 | 2.54 | Valsartan acid-d4 |
| **Verapamil** | C_27_H_38_N_2_O_4_ | [M+H]+ | 455.2904 | 165.0883 | 25 | 40 | 7.1 | Carbamazepine-d10 |


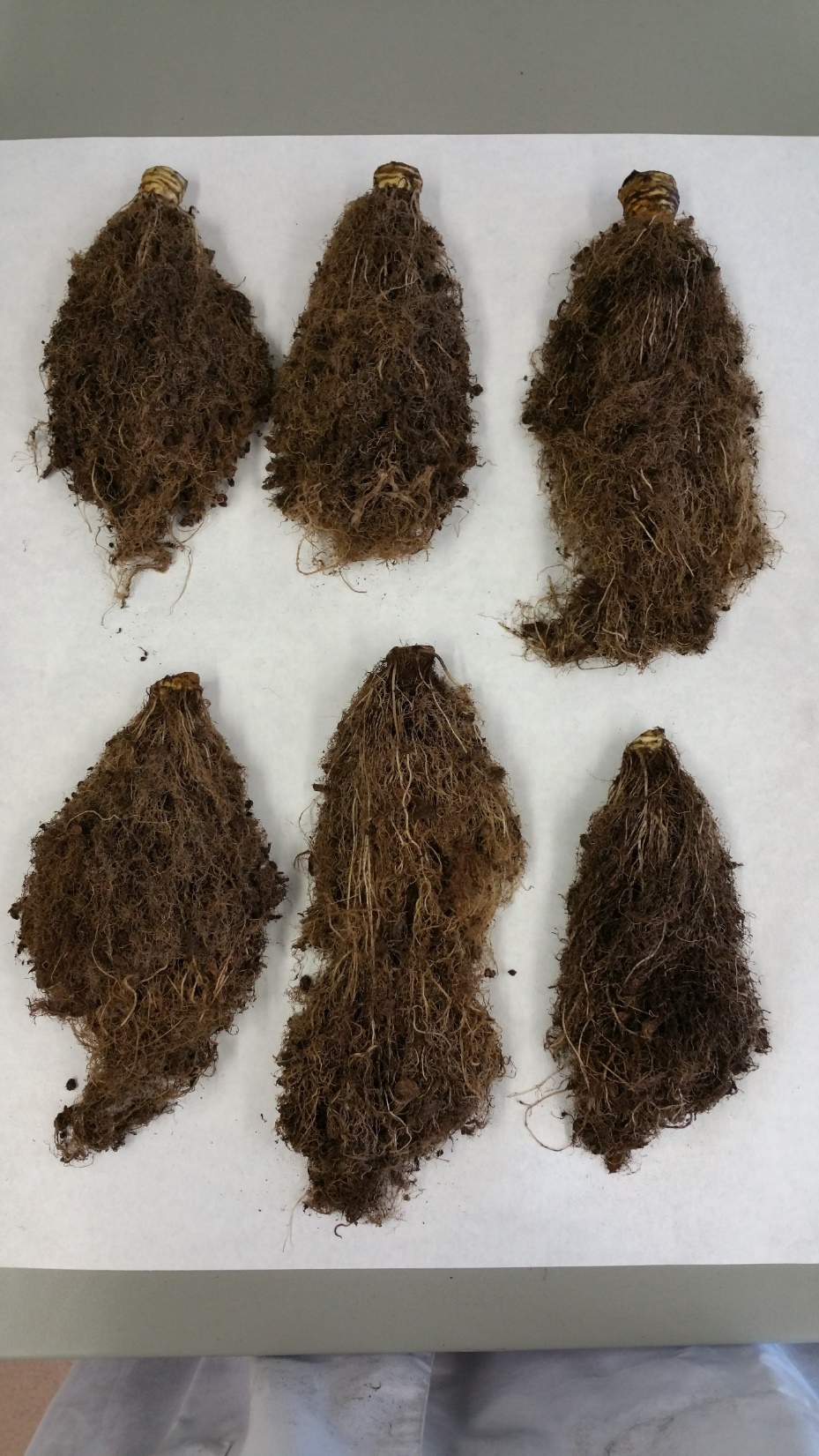


**A**

**B**

## Figure S1. Comparison of Control roots (A) and Exposed roots (B) after the harvest.

LC-MS/MS analysis:

The MRM^HR^ scanning mode was used for accurate quantification of product ion transitions. The Guided MRM^HR^ tool from SCIEX was used for the optimization of high-resolution transitions, and fragment ions. The best signal intensity was selected for quantification while the precursor was used for confirmation. The optimized ionization mode, DP, and CE for each compound have been reported in Table S3. The SWATH acquisition mode which combines in the same run data in full-scan, targeted independent MS/MS data and non-targeted independent MS/MS data acquisition, of a single TOF-MS experiment over a m/z range from 100 to 950 Da with an accumulation time of 120 ms (AT), followed by 10 MS/MS experiments with variable Q1 windows (100 to 950 m/z, 40 ms AT) using a Collision Energy (CE) of 35 V with a collision energy spread of ± 15 V, respectively for positive and negative mode of ionization. For generating SWATH Q1 variable windows, SCIEX provided an active Excel spreadsheet template known as SWATH variable window calculator (Ver. 1.0). The 10 sequential Q1 variable windows were created based on the precursor ion distribution within the retention time of the LC gradient. A sample containing all target compounds and lettuce roots or soil extracts was injected in full-scan to obtain MS survey scan. This scan contained the list of the m/z values of all precursors and the peak intensities obtained from the peaks detected in a spectrum merged by all the spectra within a same retention time window of the chromatographic gradient. Variable windows were generated by computing the number of precursor ions and considering their intensities as a weighting factor. The SWATH isolation window plot generated by this calculator based on the same MS survey scan including the variable windows were reported in Fig. S1 and Fig. S2 for lettuce root and soil, respectively. In order to evaluate the sensitivity of SWATH acquisition mode, the validation of targeted compounds was also performed acquiring data with SWATH acquisition and then both modalities were compared.

The electrospray ionization source (ESI) conditions were optimized as follow. Ion Spray Voltage was set to 5500 V (-4500 V for negative); Source temperature and nitrogen gas flows (Atomizing gas, GS1 and Auxiliary gas, GS2) were set to 550° C, 55, and 55 psi, respectively. Curtain gas was set to 30 psi, while collision gas (CAD) was set to 7. Any drift in the mass accuracy of the SCIEX Q-TOF was automatically corrected and maintained throughout batch acquisition by infusion of Reserpine reference standard (C33H40N2O9, m/z 609.28066) for positive ionization, and a cluster of trifluoroacetic acid ([5(TFA-Na)+TFA]-, m/z 792.85963) for negative mode. Calibration was running every 5 samples during the batch acquisition making use of the Calibrant Delivery System (CDS).


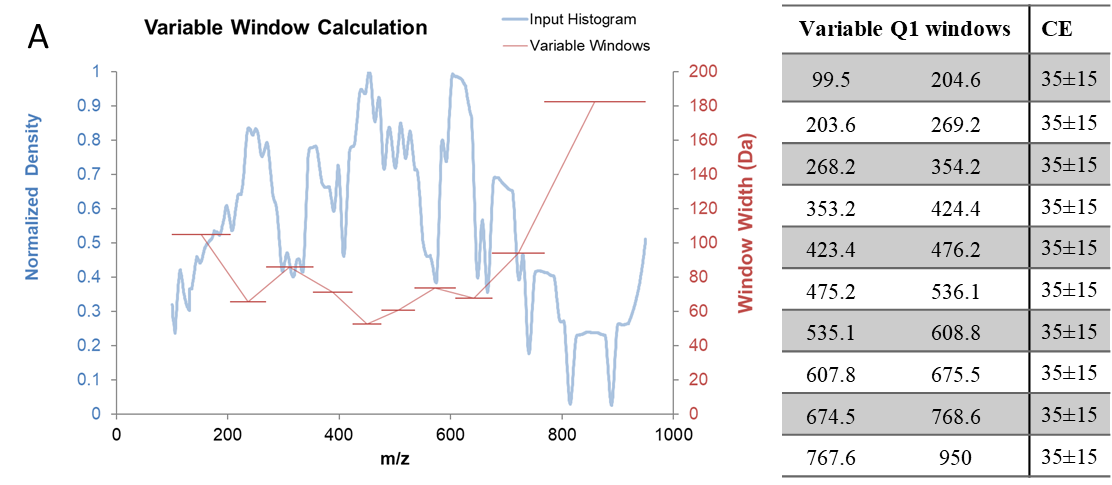


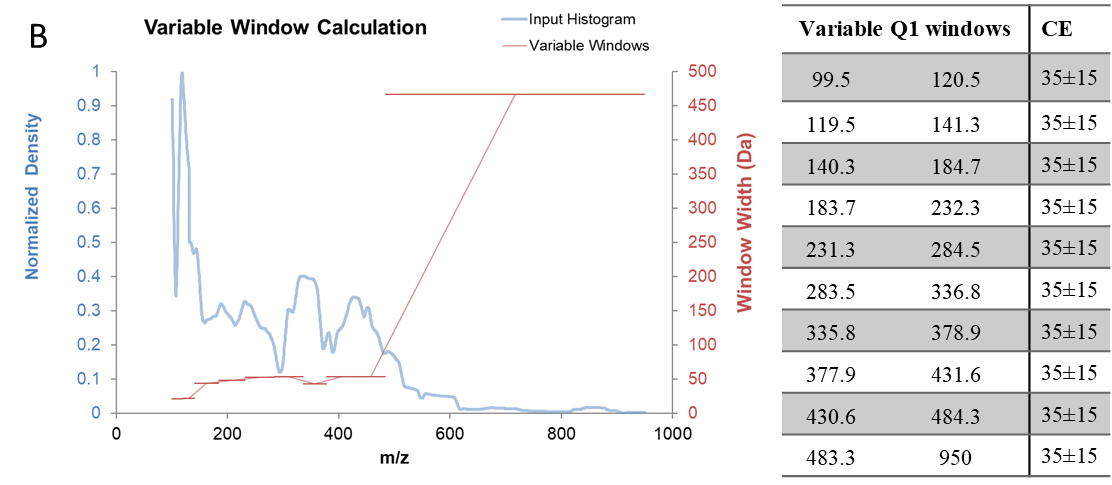


## Figure S2. Variable Q1 Window Widths for SWATH Acquisition in positive (A) and negative (B) ionization for lettuce root


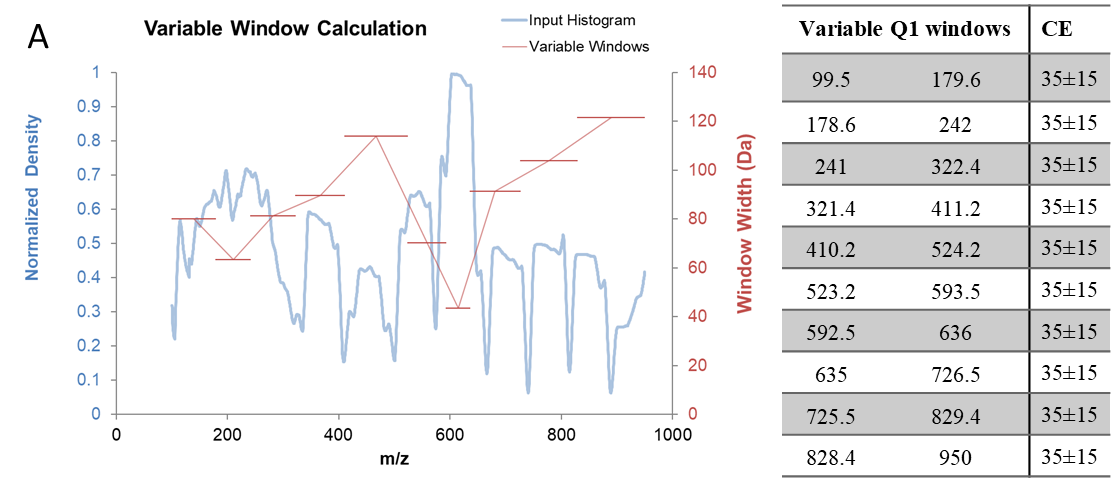


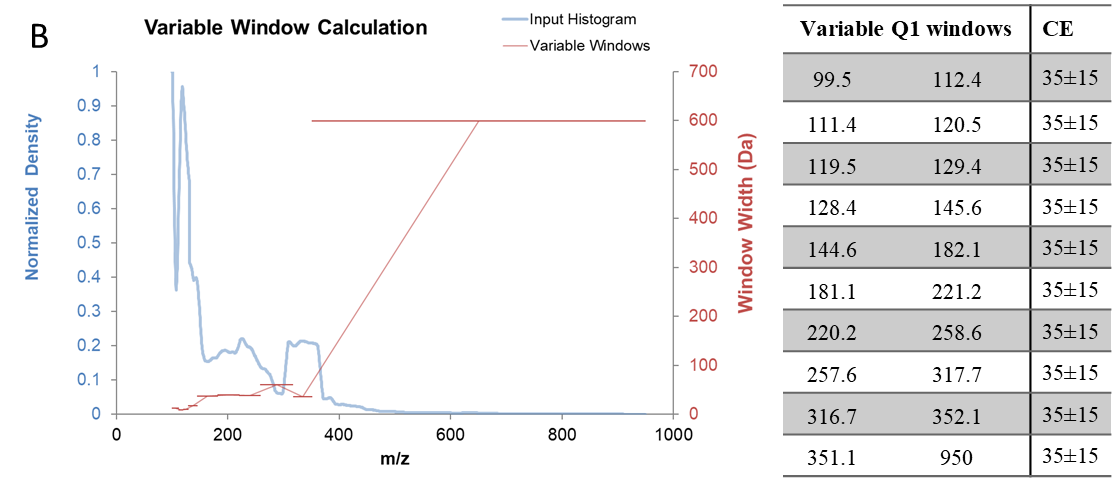


## Figure S3. Variable Q1 Window Widths for SWATH Acquisition in positive (A) and negative (B) ionization for soil

# Results and discussion:


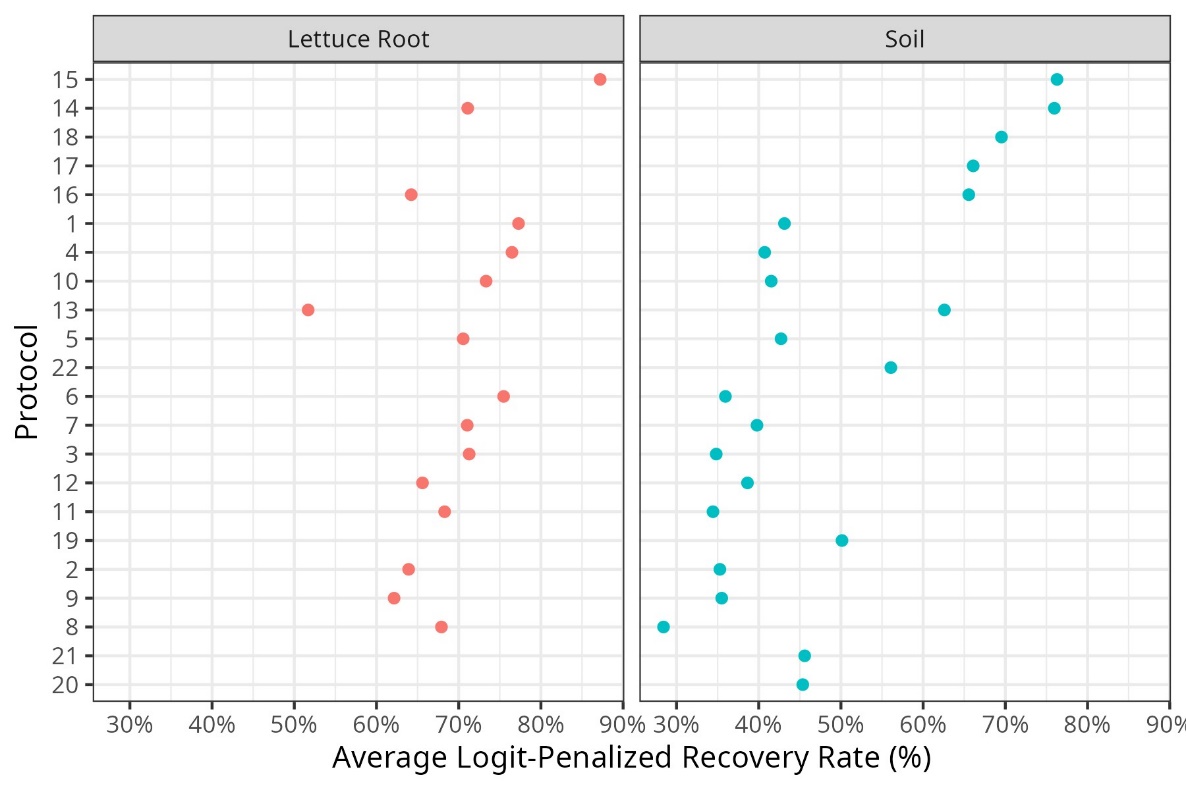


## Figure S4. Statistical comparison between the different protocols and matrices, using a loss function to optimize the protocol selection.

## Figure S5. Logit-penalized average recovery rates.

## Table S4. List of compounds with recoveries > 60%, 40-60%, 20-40%, 0-20% and ND (not detected) for lettuce root matrix

|  | **R > 60%** | **40 < R% < 60** | **20 < R% < 40** | **0 < R% < 20%** | **ND** |
| --- | --- | --- | --- | --- | --- |
| Protocol 1 | 4-hydroxydiclofenac; 4-nitro-sulfamethoxazole; 5-methyl-benzotriazole; acesulfame; acetaminophen; acridone; benzotriazole; bezafibrate; caffeine; carbamazepine-10,11-epoxide; carbamazepine; chloramphenicol; citalopram; clarithromycin; climbazole; clofibric acid; diclofenac; diltiazem; fluconazole; furosemide; gemfibrozil; hydrochlorothiazide; irbesartan; lamotrigine; metoprolol; metronidazole; N4-acetyl-sulfamethoxazole; propranolol; sucralose; sulfanilic acid; valsartan; verapamil; fipronil; fipronil sulfone; fipronil desulfinyl | lamotrigine N2-oxide; oxcarbazepine; bisphenol A; N2-methyl-lamotrigine | sulfamethazine; 5-desamino-5-oxo-2,5-dihydro-lamotrigine; sulfamethoxazole | sulfanilamide | valsartan acid; ibuprofen; ciprofloxacin; fenofibrate; indomethacin |
| Protocol 2 | 4-nitro-sulfamethoxazole; 5-desamino-5-oxo-2,5-dihydro-lamotrigine; 5-methyl-benzotriazole; acesulfame; acetaminophen; benzotriazole; bisphenol A; carbamazepine-10,11-epoxide; carbamazepine; chloramphenicol; citalopram; clarithromycin; climbazole; diltiazem; fluconazole; furosemide; gemfibrozil; hydrochlorothiazide; ibuprofen; lamotrigine N2-oxide; metoprolol; metronidazole; N4-acetyl-sulfamethoxazole; N2-methyl-lamotrigine; propranolol; sucralose; verapamil; fipronil; fipronil sulfone; fipronil desulfinyl | bezafibrate; oxcarbazepine; sulfanilic acid; irbesartan; sulfamethoxazole; acridone; caffeine; lamotrigine; diclofenac | sulfamethazine; 4-hydroxydiclofenac; clofibric acid | sulfanilamide | valsartan acid; valsartan; ciprofloxacin; fenofibrate; indomethacin |
| Protocol 3 | 4-nitro-sulfamethoxazole; 5-desamino-5-oxo-2,5-dihydro-lamotrigine; 5-methyl-benzotriazole; acetaminophen; acridone; benzotriazole; caffeine; carbamazepine-10,11-epoxide; carbamazepine; chloramphenicol; citalopram; clarithromycin; climbazole; diclofenac; diltiazem; fluconazole; gemfibrozil; hydrochlorothiazide; irbesartan; lamotrigine N2-oxide; lamotrigine; metoprolol; metronidazole; N4-acetyl-sulfamethoxazole; N2-methyl-lamotrigine; propranolol; sucralose; sulfanilic acid; verapamil; fipronil; fipronil sulfone; fipronil desulfinyl | 4-hydroxydiclofenac; bisphenol A; oxcarbazepine; furosemide; acesulfame | sulfamethazine; sulfamethoxazole; bezafibrate; clofibric acid | valsartan; sulfanilamide | ciprofloxacin; fenofibrate; ibuprofen; indomethacin; valsartan acid |
| Protocol 4 | 4-hydroxydiclofenac; 4-nitro-sulfamethoxazole; 5-desamino-5-oxo-2,5-dihydro-lamotrigine; 5-methyl-benzotriazole; acesulfame; bezafibrate; caffeine; carbamazepine-10,11-epoxide; carbamazepine; chloramphenicol; citalopram; clarithromycin; climbazole; clofibric acid; diltiazem; fluconazole; gemfibrozil; hydrochlorothiazide; lamotrigine; metoprolol; N4-acetyl-sulfamethoxazole; sucralose; sulfamethazine; sulfanilic acid; valsartan acid; valsartan; verapamil; ; fipronil; fipronil sulfone; fipronil desulfinyl | N2-methyl-lamotrigine; diclofenac; acridone; benzotriazole; irbesartan; metronidazole; sulfamethoxazole; lamotrigine N2-oxide; bisphenol A; acetaminophen | oxcarbazepine | none | ciprofloxacin; fenofibrate; furosemide; indomethacin; sulfanilamide; propranolol; sulfamethazine; ibuprofen |
| Protocol 5 | 4-hydroxydiclofenac; 4-nitro-sulfamethoxazole; 5-desamino-5-oxo-2,5-dihydro-lamotrigine; 5-methyl-benzotriazole; acesulfame; acetaminophen; benzotriazole; bezafibrate; caffeine; carbamazepine; citalopram; clarithromycin; climbazole; clofibric acid; diclofenac; diltiazem; fluconazole; gemfibrozil; hydrochlorothiazide; irbesartan; lamotrigine N2-oxide; lamotrigine; metoprolol; metronidazole; N2-methyl-lamotrigine; propranolol; sucralose; sulfanilic acid; verapamil; fipronil; fipronil sulfone; fipronil desulfinyl | bisphenol A; acridone; valsartan; carbamazepine-10,11-epoxide; N4-acetyl-sulfamethoxazole | valsartan acid; sulfamethazine; oxcarbazepine; sulfamethoxazole; chloramphenicol | sulfanilamide | ciprofloxacin; fenofibrate; ibuprofen; furosemide; indomethacin |
| Protocol 6 | 4-hydroxydiclofenac; 4-nitro-sulfamethoxazole; 5-desamino-5-oxo-2,5-dihydro-lamotrigine; 5-methyl-benzotriazole; acesulfame; acetaminophen; benzotriazole; caffeine; carbamazepine; citalopram; clarithromycin; climbazole; clofibric acid; diclofenac; diltiazem; fluconazole; gemfibrozil; hydrochlorothiazide; irbesartan; lamotrigine N2-oxide; lamotrigine; metoprolol ; metronidazole; N2-methyl-lamotrigine; propranolol; sucralose; sulfanilic acid; verapamil; fipronil; fipronil sulfone; fipronil desulfinyl | bisphenol A; acridone; carbamazepine-10,11-epoxide; N4-acetyl-sulfamethoxazole; bezafibrate | valsartan acid; valsartan; sulfamethazine; oxcarbazepine; sulfamethoxazole; chloramphenicol | sulfanilamide | fenofibrate; furosemide; indomethacin; ciprofloxacin; ibuprofen |
| Protocol 7 | 4-hydroxydiclofenac; 4-nitro-sulfamethoxazole; 5-methyl-benzotriazole; acesulfame; acetaminophen; acridone; benzotriazole; bezafibrate; carbamazepine-10,11-epoxide; carbamazepine; chloramphenicol; citalopram; clarithromycin; climbazole; clofibric acid; diclofenac; diltiazem; fluconazole; gemfibrozil; hydrochlorothiazide; irbesartan; metoprolol; metronidazole; N4-acetyl-sulfamethoxazole; propranolol; sucralose; sulfanilic acid; valsartan acid; valsartan; verapamil; fipronil; fipronil sulfone; fipronil desulfinyl | sulfamethoxazole; 5-desamino-5-oxo-2,5-dihydro-lamotrigine; lamotrigine; N2-methyl-lamotrigine; bisphenol A; caffeine | lamotrigine N2-oxide; sulfamethazine; oxcarbazepine | none | ciprofloxacin; fenofibrate; indomethacin; sulfanilamide; furosemide; ibuprofen |
| Protocol 8 | 4-nitro-sulfamethoxazole; 5-desamino-5-oxo-2,5-dihydro-lamotrigine; 5-methyl-benzotriazole; acesulfame; acetaminophen; acridone; benzotriazole; bisphenol A; caffeine; carbamazepine-10,11-epoxide; carbamazepine; chloramphenicol; citalopram; clarithromycin; climbazole; diltiazem; fluconazole; furosemide; gemfibrozil; hydrochlorothiazide; irbesartan; lamotrigine; metoprolol; metronidazole; N4-acetyl-sulfamethoxazole; propranolol; sucralose; verapamil; fipronil; fipronil sulfone; fipronil desulfinyl | oxcarbazepine; clofibric acid; sulfanilic acid; N2-methyl-lamotrigine; diclofenac | sulfamethazine; bezafibrate; sulfamethoxazole; 4-hydroxydiclofenac; lamotrigine N2-oxide | sulfanilamide | valsartan acid; fenofibrate; ibuprofen; indomethacin; valsartan; ciprofloxacin |
| Protocol 9 | 4-nitro-sulfamethoxazole; 5-methyl-benzotriazole; acesulfame; acetaminophen; acridone; benzotriazole; caffeine; carbamazepine-10,11-epoxide; carbamazepine; chloramphenicol; citalopram; clarithromycin; climbazole; diclofenac; diltiazem; fluconazole; hydrochlorothiazide; irbesartan; lamotrigine; metoprolol; N4-acetyl-sulfamethoxazole; N2-methyl-lamotrigine; propranolol; sucralose; sulfanilic acid; fipronil; fipronil sulfone; fipronil desulfinyl | clofibric acid; oxcarbazepine; bisphenol A; sulfamethoxazole; lamotrigine N2-oxide; gemfibrozil; metronidazole | 4-hydroxydiclofenac; bezafibrate; 5-desamino-5-oxo-2,5-dihydro-lamotrigine | sulfanilamide; sulfamethazine | valsartan acid; valsartan; ciprofloxacin; fenofibrate; ibuprofen; indomethacin; furosemide |
| Protocol 10 | 4-hydroxydiclofenac; 4-nitro-sulfamethoxazole; 5-methyl-benzotriazole; acesulfame; acridone; bezafibrate; bisphenol A; carbamazepine-10,11-epoxide; carbamazepine; chloramphenicol; citalopram; clarithromycin; climbazole; clofibric acid; diclofenac; diltiazem; fluconazole; gemfibrozil; hydrochlorothiazide; irbesartan; metoprolol; metronidazole; N4-acetyl-sulfamethoxazole; N2-methyl-lamotrigine; propranolol; sucralose; valsartan acid; valsartan; verapamil; fipronil; fipronil sulfone; fipronil desulfinyl | sulfanilic acid; benzotriazole; caffeine; 5-desamino-5-oxo-2,5-dihydro-lamotrigine; acetaminophen; lamotrigine | sulfamethazine; sulfamethoxazole; lamotrigine N2-oxide; oxcarbazepine | none | ciprofloxacin; fenofibrate; furosemide; indomethacin; sulfanilamide; ibuprofen |
| Protocol 11 | 4-nitro-sulfamethoxazole; 5-desamino-5-oxo-2,5-dihydro-lamotrigine; 5-methyl-benzotriazole; benzotriazole; caffeine; carbamazepine; citalopram; clarithromycin; climbazole; diclofenac; diltiazem; fluconazole; hydrochlorothiazide; irbesartan; lamotrigine N2-oxide; metoprolol; N4-acetyl-sulfamethoxazole; N2-methyl-lamotrigine; propranolol; sucralose; sulfanilic acid; verapamil; fipronil; fipronil sulfone; fipronil desulfinyl | acetaminophen; bisphenol A; gemfibrozil; clofibric acid; acridone; chloramphenicol; bezafibrate; metronidazole; 4-hydroxydiclofenac; lamotrigine; carbamazepine-10,11-epoxide; acesulfame | sulfamethazine; oxcarbazepine; sulfamethoxazole | sulfanilamide | valsartan acid; valsartan; ciprofloxacin; fenofibrate; ibuprofen; indomethacin; furosemide |
| Protocol 12 | 5-methyl-benzotriazole; acesulfame; acetaminophen; acridone; caffeine; carbamazepine-10,11-epoxide; carbamazepine; chloramphenicol; citalopram; clarithromycin; climbazole; diclofenac; diltiazem; fluconazole; hydrochlorothiazide; irbesartan; metoprolol; metronidazole; N4-acetyl-sulfamethoxazole; N2-methyl-lamotrigine; propranolol; sucralose; verapamil; fipronil; fipronil sulfone; fipronil desulfinyl | clofibric acid; bisphenol A; sulfamethoxazole; sulfanilic acid; 5-desamino-5-oxo-2,5-dihydro-lamotrigine; lamotrigine; 4-hydroxydiclofenac; 4-nitro-sulfamethoxazole; gemfibrozil | benzotriazole; sulfamethazine; bezafibrate; oxcarbazepine; lamotrigine N2-oxide | sulfanilamide | valsartan acid; valsartan; fenofibrate; furosemide; indomethacin; ciprofloxacin; ibuprofen |
| Protocol 13 | N4-acetyl-sulfamethoxazole; 5-desamino-5-oxo-2,5-dihydro-lamotrigine; irbesartan; sulfanilic acid; 4-nitro-sulfamethoxazole; chloramphenicol; sucralose; hydrochlorothiazide; 5-methyl-benzotriazole; carbamazepine; benzotriazole; gemfibrozil; fluconazole; furosemide; fipronil; fipronil sulfone; fipronil desulfinyl | 4-hydroxydiclofenac; acesulfame; acetaminophen; bezafibrate; caffeine; carbamazepine-10,11-epoxide; citalopram; clarithromycin; climbazole; clofibric acid; diclofenac; diltiazem; lamotrigine; metoprolol; metronidazole; propranolol; valsartan; verapamil | sulfamethoxazole; oxcarbazepine; sulfamethazine; acridone; N2-methyl-lamotrigine; bisphenol A; caffeine; lamotrigine N2-oxide | valsartan acid; sulfanilamide | ciprofloxacin; fenofibrate; ibuprofen; indomethacin |
| Protocol 14 | 4-hydroxydiclofenac; 4-nitro-sulfamethoxazole; 5-desamino-5-oxo-2,5-dihydro-lamotrigine; 5-methyl-benzotriazole; acetaminophen; acridone; bezafibrate; carbamazepine-10,11-epoxide; carbamazepine; chloramphenicol; citalopram; clarithromycin; climbazole; clofibric acid; diclofenac; diltiazem; fluconazole; gemfibrozil; hydrochlorothiazide; irbesartan; lamotrigine; metoprolol; metronidazole; N4-acetyl-sulfamethoxazole; N2-methyl-lamotrigine; propranolol; sucralose; valsartan; verapamil; fipronil; fipronil sulfone; fipronil desulfinyl | bisphenol A; caffeine; acesulfame; sulfanilic acid; benzotriazole; lamotrigine N2-oxide | sulfamethazine; sulfamethoxazole; oxcarbazepine | sulfanilamide | ciprofloxacin; valsartan acid; fenofibrate; indomethacin; furosemide; ibuprofen |
| Protocol 15 | 4-hydroxydiclofenac; 4-nitro-sulfamethoxazole; 5-desamino-5-oxo-2,5-dihydro-lamotrigine; 5-methyl-benzotriazole; acesulfame; acetaminophen; acridone; benzotriazole; bezafibrate; bisphenol A; caffeine; carbamazepine-10,11-epoxide; carbamazepine; chloramphenicol; citalopram; clarithromycin; climbazole; clofibric acid; diclofenac; diltiazem; fenofibrate; fluconazole; furosemide; gemfibrozil; hydrochlorothiazide; ibuprofen; indomethacin; irbesartan; lamotrigine N2-oxide; lamotrigine; metoprolol; metronidazole; N4-acetyl-sulfamethoxazole; N2-methyl-lamotrigine; oxcarbazepine; propranolol; sucralose; sulfanilic acid; valsartan acid; valsartan; verapamil; fipronil; fipronil sulfone; fipronil desulfinyl | none | ciprofloxacin; sulfamethoxazole | sulfamethazine | sulfanilamide |
| Protocol 16 | 4-hydroxydiclofenac; 4-nitro-sulfamethoxazole; 5-desamino-5-oxo-2,5-dihydro-lamotrigine; acesulfame; acetaminophen; acridone; bezafibrate; bisphenol A; caffeine; carbamazepine-10,11-epoxide; carbamazepine; chloramphenicol; clofibric acid; fluconazole; hydrochlorothiazide; metoprolol; metronidazole; N4-acetyl-sulfamethoxazole; oxcarbazepine; sucralose; sulfamethoxazole; valsartan acid; valsartan; fipronil; fipronil sulfone; fipronil desulfinyl | clarithromycin; N2-methyl-lamotrigine; 5-methyl-benzotriazole; diclofenac; benzotriazole; lamotrigine N2-oxide; gemfibrozil; climbazole | citalopram; irbesartan; diltiazem; propranolol; lamotrigine | verapamil | ciprofloxacin; sulfamethazine;  ibuprofen; furosemide; indomethacin; sulfanilamide; sulfanilic acid; fenofibrate |

## Table S5. List of compounds with recoveries > 60%, 40-60%, 20-40%, 0-20% and ND (not detected) for soil matrix

|  | **R > 60%** | **40 < R% < 60** | **20 < R% < 40** | **0 < R% < 20%** | **ND** |
| --- | --- | --- | --- | --- | --- |
| Protocol 1 | caffeine; valsartan; chloramphenicol; irbesartan; sucralose; climbazole; fipronil; fipronil sulfone; fipronil desulfinyl | 4-nitro-sulfamethoxazole; acesulfame; acridone; bisphenol A; carbamazepine-10,11-epoxide; citalopram; diclofenac; diltiazem; fluconazole; furosemide; gemfibrozil; hydrochlorothiazide; lamotrigine N2-oxide; lamotrigine; metoprolol; metronidazole; N4-acetyl-sulfamethoxazole; propranolol; sulfamethoxazole; sulfanilamide; verapamil | acetaminophen; 4-hydroxydiclofenac; ibuprofen; sulfamethazine; clarithromycin; N2-methyl-lamotrigine; sulfanilic acid; clofibric acid; carbamazepine; bezafibrate; 5-desamino-5-oxo-2,5-dihydro-lamotrigine | ciprofloxacin; oxcarbazepine | benzotriazole; indomethacin; valsartan acid; 5-methyl-benzotriazole; fenofibrate |
| Protocol 2 | caffeine; fipronil; fipronil sulfone; fipronil desulfinyl | 4-nitro-sulfamethoxazole; 5-methyl-benzotriazole; acesulfame; acridone; benzotriazole; bisphenol A; carbamazepine-10,11-epoxide; carbamazepine; clarithromycin; diclofenac; diltiazem; fluconazole; gemfibrozil; hydrochlorothiazide; ibuprofen; irbesartan; lamotrigine N2-oxide; lamotrigine; metronidazole; oxcarbazepine; sulfamethazine; sulfamethoxazole; sulfanilic acid | sulfanilic acid; acetaminophen; clarithromycin; verapamil; citalopram; propranolol; metoprolol; N4-acetyl-sulfamethoxazole; sucralose; N2-methyl-lamotrigine; chloramphenicol; 5-desamino-5-oxo-2,5-dihydro-lamotrigine; climbazole; sulfanilamide | ciprofloxacin; 4-hydroxydiclofenac; bezafibrate; clofibric acid | Fenofibrate; furosemide; indomethacin; valsartan acid; valsartan |
| Protocol 3 | caffeine; fipronil; fipronil sulfone; fipronil desulfinyl | 4-nitro-sulfamethoxazole; acesulfame; acridone; benzotriazole; bisphenol A; carbamazepine-10,11-epoxide; carbamazepine; diclofenac; diltiazem; fluconazole; gemfibrozil; hydrochlorothiazide; irbesartan; lamotrigine; metoprolol; oxcarbazepine; sulfamethazine; sulfamethoxazole; sulfanilic acid | clofibric acid; 5-methyl-benzotriazole; acetaminophen; clarithromycin;  5-desamino-5-oxo-2,5-dihydro-lamotrigine; verapamil; propranolol; metronidazole; citalopram; climbazole; lamotrigine N2-oxide; N2-methyl-lamotrigine; sulfanilamide; N4-acetyl-sulfamethoxazole; chloramphenicol; | valsartan; ciprofloxacin; 4-hydroxydiclofenac; bezafibrate; furosemide | fenofibrate; ibuprofen; indomethacin; sucralose; valsartan acid |
| Protocol 4 | Verapamil; caffeine; climbazole; fipronil; fipronil sulfone; fipronil desulfinyl | 4-hydroxydiclofenac; 4-nitro-sulfamethoxazole; 5-methyl-benzotriazole; acridone; benzotriazole; bezafibrate; bisphenol A; carbamazepine; citalopram; diltiazem; fluconazole; gemfibrozil; ibuprofen; lamotrigine; metoprolol;metronidazole; N4-acetyl-sulfamethoxazole; N2-methyl-lamotrigine; propranolol; sulfanilic acid; valsartan; | sulfamethoxazole; lamotrigine N2-oxide; valsartan acid; acetaminophen; oxcarbazepine; clarithromycin; chloramphenicol; sucralose; 5-desamino-5-oxo-2,5-dihydro-lamotrigine; carbamazepine-10,11-epoxide; diclofenac; hydrochlorothiazide; acesulfame; irbesartan; clofibric acid | ciprofloxacin; sulfanilamide; sulfamethazine | fenofibrate; indomethacin; furosemide; |
| Protocol 5 | bisphenol A; 5-methyl-benzotriazole; metronidazole; benzotriazole; metoprolol; ibuprofen; caffeine; sucralose; fipronil; fipronil sulfone; fipronil desulfinyl | N4-acetyl-sulfamethoxazole; acesulfame; sulfanilic acid; sulfamethazine; N2-methyl-lamotrigine; diltiazem; fluconazole; gemfibrozil; climbazole; carbamazepine-10,11-epoxide; hydrochlorothiazide; citalopram; propranolol; verapamil | 4-nitro-sulfamethoxazole; 5-desamino-5-oxo-2,5-dihydro-lamotrigine; acesulfame; acetaminophen; acridone; carbamazepine; chloramphenicol; clarithromycin; clofibric acid; diclofenac; irbesartan; lamotrigine N2-oxide; lamotrigine; oxcarbazepine; sulfamethoxazole; sulfanilamide | ciprofloxacin; 4-hydroxydiclofenac; bezafibrate | Fenofibrate; furosemide; indomethacin; valsartan acid; valsartan |
| Protocol 6 | caffeine; sulfanilic acid; fipronil; fipronil sulfone; fipronil desulfinyl | propranolol; 4-nitro-sulfamethoxazole; sulfamethazine; benzotriazole; carbamazepine; acridone; oxcarbazepine; metoprolol;5-desamino-5-oxo-2,5-dihydro-lamotrigine; sulfamethoxazole; fluconazole; metronidazole; verapamil;carbamazepine-10,11-epoxide; 5-methyl-benzotriazole; bisphenol A | acesulfame; acetaminophen; chloramphenicol; citalopram; clarithromycin; climbazole; diclofenac; diltiazem; gemfibrozil; hydrochlorothiazide; irbesartan; lamotrigine N2-oxide; lamotrigine; N4-acetyl-sulfamethoxazole; N2-methyl-lamotrigine; sulfanilamide | ciprofloxacin; bezafibrate; 4-hydroxydiclofenac; clofibric acid | fenofibrate; furosemide; ibuprofen; indomethacin; sucralose; valsartan acid; valsartan |
| Protocol 7 | carbamazepine; sulfanilic acid; 4-hydroxydiclofenac; caffeine; N4-acetyl-sulfamethoxazole; gemfibrozil; chloramphenicol; climbazole; bisphenol A; fipronil; fipronil sulfone; fipronil desulfinyl | propranolol; diltiazem; fluconazole; 5-desamino-5-oxo-2,5-dihydro-lamotrigine; irbesartan; acesulfame; verapamil; 4-nitro-sulfamethoxazole; bezafibrate; citalopram; diclofenac; hydrochlorothiazide; valsartan | 5-methyl-benzotriazole; acridone; benzotriazole; carbamazepine-10,11-epoxide; clarithromycin; clofibric acid; furosemide; lamotrigine; metoprolol; metronidazole; N2-methyl-lamotrigine; oxcarbazepine; sulfamethazine; sulfamethoxazole; sulfanilamide; valsartan acid | ciprofloxacin; sucralose; lamotrigine N2-oxide; acetaminophen | fenofibrate; ibuprofen; indomethacin |
| Protocol 8 | ibuprofen; fenofibrate; metronidazole; bisphenol A; fipronil; fipronil sulfone; fipronil desulfinyl | sulfanilic acid; sulfamethazine; carbamazepine-10,11-epoxide; benzotriazole; acridone; chloramphenicol; gemfibrozil; oxcarbazepine; caffeine; fluconazole | 4-nitro-sulfamethoxazole; 5-desamino-5-oxo-2,5-dihydro-lamotrigine; 5-methyl-benzotriazole; acesulfame; carbamazepine; citalopram; clarithromycin; climbazole; diclofenac; diltiazem; furosemide; hydrochlorothiazide; indomethacin; lamotrigine; metoprolol;37. N4-acetyl-sulfamethoxazole; N2-methyl-lamotrigine; propranolol; sucralose; sulfamethoxazole; sulfanilamide; valsartan acid; valsartan; verapamil | ciprofloxacin;4-hydroxydiclofenac; acetaminophen; bezafibrate; clofibric acid; irbesartan; lamotrigine N2-oxide |  |
| Protocol 9 | sucralose; fenofibrate; ibuprofen; caffeine; sulfanilic acid; bisphenol A; fipronil; fipronil sulfone; fipronil desulfinyl | N4-acetyl-sulfamethoxazole; sulfamethazine; citalopram; verapamil; metoprolol; oxcarbazepine; acridone; chloramphenicol; benzotriazole; sulfamethoxazole; hydrochlorothiazide; fluconazole; carbamazepine | 4-nitro-sulfamethoxazole; 5-desamino-5-oxo-2,5-dihydro-lamotrigine; 5-methyl-benzotriazole; acesulfame; bezafibrate; carbamazepine-10,11-epoxide; clarithromycin; climbazole; diltiazem; gemfibrozil; irbesartan; lamotrigine N2-oxide; lamotrigine; metronidazole; N2-methyl-lamotrigine; propranolol; sulfanilamide | ciprofloxacin; 4-hydroxydiclofenac; diclofenac; acetaminophen; clofibric acid | indomethacin; valsartan acid; valsartan; furosemide |
| Protocol 10 | citalopram; propranolol; climbazole; sulfanilic acid; fipronil; fipronil sulfone; fipronil desulfinyl | 4-nitro-sulfamethoxazole; 5-desamino-5-oxo-2,5-dihydrolamotrigine; 5-methyl-benzotriazole; acesulfame; acridone; bezafibrate; bisphenol A; caffeine; carbamazepine; chloramphenicol; clarithromycin; clofibric acid; diltiazem; fluconazole; gemfibrozil; ibuprofen; lamotrigine; metoprolol; N4-acetyl-sulfamethoxazole; N2-methyl-lamotrigine; sulfamethoxazole; valsartan acid; valsartan; verapamil | carbamazepine-10,11-epoxide; irbesartan; diclofenac; benzotriazole; metronidazole; oxcarbazepine; sulfamethazine; furosemide; sucralose; hydrochlorothiazide | ciprofloxacin; acetaminophen; 4-hydroxydiclofenac; lamotrigine N2-oxide; sulfanilamide | fenofibrate; indomethacin |
| Protocol 11 | metoprolol; caffeine; gemfibrozil; sucralose; fenofibrate; fipronil; fipronil sulfone; fipronil desulfinyl | 5-methyl-benzotriazole;  acridone; benzotriazole;  bisphenol A; carbamazepine-10,11-epoxide; carbamazepine; clarithromycin; diclofenac; fluconazole; hydrochlorothiazide; ibuprofen; metronidazole; N4-acetyl-sulfamethoxazole; oxcarbazepine; sulfanilic acid | clofibric acid; bezafibrate; sulfamethoxazole; chloramphenicol; acesulfame; climbazole; 5-desamino-5-oxo-2,5-dihydro-lamotrigine; propranolol; irbesartan; citalopram; verapamil; lamotrigine; N2-methyl lamotrigine; 4-nitro-sulfamethoxazole; diltiazem | acetaminophen; ciprofloxacin; sulfanilamide; lamotrigine N2-oxide; 4-hydroxydiclofenac; sulfamethazine | indomethacin; valsartan acid; valsartan; furosemide |
| Protocol 12 | fenofibrate; gemfibrozil; verapamil; sulfanilic acid; fipronil; fipronil sulfone; fipronil desulfinyl | 4-nitro-sulfamethoxazole; 5-methyl-benzotriazole; acridone; benzotriazole; bezafibrate; bisphenol A; caffeine; carbamazepine-10,11-epoxide; carbamazepine; chloramphenicol; citalopram; clarithromycin; climbazole; diclofenac; diltiazem; hydrochlorothiazide; lamotrigine; metoprolol; N4-acetyl-sulfamethoxazole; oxcarbazepine; propranolol; | bezafibrate; clofibric acid; acetaminophen; lamotrigine N2-oxide; sulfamethazine; sulfamethoxazole; irbesartan; acesulfame; N2-methyl-lamotrigine; 5-desamino-5-oxo-2,5-dihydro-lamotrigine; fluconazole; metronidazole; sucralose; furosemide | 4-hydroxydiclofenac; ciprofloxacin; sulfanilamide | valsartan acid; valsartan; indomethacin; ibuprofen |
| Protocol 13 | acridone; diclofenac; valsartan; hydrochlorothiazide; bisphenol A; sulfamethoxazole; citalopram; acesulfame; 4-nitro-sulfamethoxazole; sucralose; diltiazem; N4-acetyl-sulfamethoxazole; metronidazole; irbesartan; carbamazepine-10,11-epoxide; propranolol; caffeine; carbamazepine; chloramphenicol; 5-desamino-5-oxo-2,5-dihydro-lamotrigine; gemfibrozil; ibuprofen; fluconazole; fipronil; fipronil sulfone; fipronil desulfinyl | lamotrigine N2-oxide; 4-hydroxydiclofenac; acetaminophen; bezafibrate; clarithromycin; N2-methyl-lamotrigine; sulfanilamide; clofibric acid; sulfamethazine; climbazole; lamotrigine; verapamil; metoprolol | benzotriazole; 5-methyl-benzotriazole | oxcarbazepine | valsartan acid; fenofibrate; furosemide; indomethacin; sulfanilic acid; ciprofloxacin |
| Protocol 14 | lamotrigine; N2-Methyl-Lamotrigine.; clarithromycin; acetaminophen; clofibric acid; sulfanilamide: citalopram; benzotriazole;  metronidazole; gemfibrozil; 4-nitro-sulfamethoxazole; sulfamethazine; irbesartan; acridone; acesulfame; diltiazem; ibuprofen; sucralose; propranolol; valsartan; sulfamethoxazole; hydrochlorothiazide; N4-acetyl-sulfamethoxazole; carbamazepine; carbamazepine-10,11-epoxide; fluconazole; diclofenac; verapamil; metoprolol; chloramphenicol; climbazole; bisphenol A; caffeine; gemfibrozil; ibuprofen; fipronil; fipronil sulfone; fipronil desulfinyl | 4-hydroxydiclofenac; lamotrigine N2-oxide; bezafibrate; 5-methyl-benzotriazole | none | oxcarbazepine | 5-desamino-5-oxo-2,5-dihydro-lamotrigine; valsartan acid; fenofibrate; furosemide; indomethacin; sulfanilic acid; ciprofloxacin |
| Protocol 15 | benzotriazole; diltiazem; 5-desamino-5-oxo-2,5-dihydro-lamotrigine; sulfanilic acid; metronidazole; diclofenac; fluconazole; lamotrigine N2-oxide; metoprolol; irbesartan; ibuprofen; valsartan acid; N4-acetyl-sulfamethoxazole; carbamazepine; clarithromycin; verapamil; carbamazepine-10,11-epoxide; 4-nitro-sulfamethoxazole; acesulfame; valsartan; indomethacin; sucralose; fenofibrate; caffeine; hydrochlorothiazide; climbazole; chloramphenicol; clofibric acid; furosemide; acetaminophen; bezafibrate; gemfibrozil; bisphenol A; fipronil; fipronil sulfone; fipronil desulfinyl | sulfamethoxazole; lamotrigine; 5-methyl-benzotriazole; citalopram; N2-methyl-lamotrigine; acridone; propranolol | 4-hydroxydiclofenac; sulfamethazine | ciprofloxacin; oxcarbazepine; sulfanilamide | none |
| Protocol 16 | 4-hydroxydiclofenac; 4-nitro-sulfamethoxazole; 5-desamino-5-oxo-2,5-dihydro-lamotrigine; 5-methyl-benzotriazole; acesulfame; acetaminophen; acridone; benzotriazole; bezafibrate; bisphenol A; caffeine; carbamazepine-10,11-epoxide; carbamazepine; citalopram; clarithromycin; clofibric acid; diclofenac; diltiazem; fluconazole; gemfibrozil; hydrochlorothiazide; ibuprofen; irbesartan; lamotrigine N2-oxide; lamotrigine; metoprolol; metronidazole; N4-acetyl-sulfamethoxazole; N2-methyl-lamotrigine; propranolol; sucralose; sulfamethazine; valsartan acid; valsartan; verapamil; fipronil; fipronil sulfone; fipronil desulfinyl |  | sulfamethoxazole;  sulfanilamide; climbazole | oxcarbazepine | fenofibrate; chloramphenicol; furosemide; indomethacin; sulfanilic acid; ciprofloxacin; |
| Protocol 17 | bezafibrate; sulfamethoxazole; diclofenac; lamotrigine; acesulfame; chloramphenicol; valsartan; ibuprofen; citalopram; verapamil; acridone; benzotriazole; irbesartan; N2-methyl-lamotrigine; carbamazepine; lamotrigine N2-oxide; 4-nitro-sulfamethoxazole; diltiazem; sucralose; fluconazole; N4-acetyl-sulfamethoxazole; metoprolol; gemfibrozil; bisphenol A; climbazole; caffeine; carbamazepine-10,11-epoxide; hydrochlorothiazide; propranolol; chloramphenicol; fipronil; fipronil sulfone; fipronil desulfinyl | 4-hydroxydiclofenac; acetaminophen; sulfanilamide; 5-desamino-5-oxo-2,5-dihydro-lamotrigine; metronidazole; sulfamethazine; clofibric acid; clarithromycin | 5-methyl-benzotriazole | oxcarbazepine | fenofibrate; furosemide; valsartan acid; indomethacin; sulfanilic acid; ciprofloxacin |
| Protocol 18 | sulfamethazine; 5-methyl-benzotriazole; benzotriazole; lamotrigine; sulfamethoxazole; acesulfame; clarithromycin; metronidazole; lamotrigine N2-oxide; 4-nitro-sulfamethoxazole; verapamil; citalopram; caffeine; propranolol; diltiazem; hydrochlorothiazide; bezafibrate; acridone; sucralose; N2-methyl-lamotrigine; climbazole; carbamazepine-10,11-epoxide; N4-acetyl-sulfamethoxazole; metoprolol; diclofenac; fluconazole; bisphenol A; carbamazepine; valsartan; irbesartan; fipronil; fipronil sulfone; fipronil desulfinyl | sulfanilamide; 4-hydroxydiclofenac; 5-desamino-5-oxo-2,5-dihydro-lamotrigine; acetaminophen; clofibric acid | none | oxcarbazepine | fenofibrate; furosemide; valsartan acid; indomethacin; sulfanilic acid; ciprofloxacin |
| Protocol 19 | clofibric acid; lamotrigine; climbazole; irbesartan; 5-desamino-5-oxo-2,5-dihydro-lamotrigine; metronidazole; fluconazole; 4-nitro-sulfamethoxazole; N2-methyl-lamotrigine; sucralose; acesulfame; diltiazem; metoprolol; acridone; citalopram; caffeine; carbamazepine; carbamazepine-10,11-epoxide; clarithromycin; verapamil; hydrochlorothiazide; diclofenac; N4-acetyl-sulfamethoxazole; bisphenol A; fipronil; fipronil sulfone; fipronil desulfinyl | sulfamethazine; acetaminophen; valsartan; sulfamethoxazole; propranolol | 5-methyl-benzotriazole; lamotrigine N2-oxide; fenofibrate; sulfanilamide; benzotriazole; 4-hydroxydiclofenac | oxcarbazepine; bezafibrate; indomethacin; chloramphenicol; ibuprofen; gemfibrozil | ciprofloxacin; furosemide; sulfanilic acid; valsartan acid; |
| Protocol 20 | fluconazole; diltiazem; metoprolol; 4-nitro-sulfamethoxazole; diclofenac; hydrochlorothiazide; sucralose; propranolol; verapamil; 4-nitro-sulfamethoxazole; bisphenol A; carbamazepine; acridone; acesulfame; carbamazepine-10,11-epoxide; caffeine; benzotriazole; metronidazole; fipronil; fipronil sulfone; fipronil desulfinyl | sulfamethoxazole; sulfanilamide; acetaminophen; sulfamethazine; clofibric acid; citalopram; climbazole; lamotrigine; irbesartan; N2-methyl-lamotrigine; clarithromycin | lamotrigine N2-oxide; fenofibrate; 5-desamino-5-oxo-2,5-dihydro-lamotrigine; 4-hydroxydiclofenac; 5-methyl-benzotriazole; valsartan | ibuprofen; bezafibrate; oxcarbazepine; gemfibrozil; chloramphenicol | ciprofloxacin; valsartan acid; furosemide; indomethacin; sulfanilic acid |
| Protocol 21 | valsartan; benzotriazole; diltiazem; metronidazole; 4-nitro-sulfamethoxazole; irbesartan; carbamazepine-10,11-epoxide; valsartan acid; sucralose; 5-desamino-5-oxo-2,5-dihydro-lamotrigine; citalopram; acridone; clarithromycin; fluconazole; carbamazepine; bisphenol A; clofibric acid; hydrochlorothiazide; N4-acetyl-sulfamethoxazole; diclofenac; caffeine; verapamil; acesulfame; fipronil; fipronil sulfone; fipronil desulfinyl | propranolol; acetaminophen; N2-methyl-lamotrigine; 5-methyl-benzotriazole; metoprolol | sulfamethazine; 4-hydroxydiclofenac; lamotrigine N2-oxide; lamotrigine; climbazole; sulfamethoxazole | bezafibrate; chloramphenicol; oxcarbazepine; gemfibrozil; ibuprofen; sulfanilamide; | ciprofloxacin; furosemide; sulfanilic acid; indomethacin; fenofibrate |
| Protocol 22 | sulfamethazine; lamotrigine; citalopram; benzotriazole; 5-methyl-benzotriazole; propranolol; caffeine; climbazole; 4-nitro-sulfamethoxazole; N4-acetyl-sulfamethoxazole; metronidazole; diltiazem; sucralose; acridone; fluconazole; acesulfame; 5-desamino-5-oxo-2,5-dihydro-lamotrigine; metoprolol; irbesartan; diclofenac; hydrochlorothiazide; carbamazepine-10,11-epoxide; carbamazepine; bisphenol A; verapamil; clarithromycin; fipronil; fipronil sulfone; fipronil desulfinyl | 4-hydroxydiclofenac; valsartan; sulfanilamide; N2-methyl-lamotrigine; acetaminophen; clofibric acid; sulfamethoxazole | lamotrigine N2-oxide | indomethacin; bezafibrate; chloramphenicol; oxcarbazepine; gemfibrozil; ibuprofen | fenofibrate; ciprofloxacin; valsartan acid; furosemide; sulfanilic acid |

## Table S6. Comparison of Matrix effect (%) for target analytes in MRMHR and SWATH acquisition modes in lettuce root matrix

| **Compound** | **ESI** | **MRM^HR^ acquisition mode** | | | | | **SWATH acquisition mode** | | | | |
| --- | --- | --- | --- | --- | --- | --- | --- | --- | --- | --- | --- |
|  |  | **2 µg L^-1^** | **5 µg L^-1^** | **10 µg L^-1^** | **50 µg L^-1^** | **200 µg L^-1^** | **2 µg L^-1^** | **5 µg L^-1^** | **10 µg L^-1^** | **50 µg L^-1^** | **200 µg L^-1^** |
| **Acesulfame** | - | 115.1 | 34.9 | 20.9 | 30.7 | 5.5 | 97.1 | 60.2 | 54.1 | 27.2 | 7.8 |
| **Acetaminophen** | - | 200.9 | 141.8 | 192.8 | 165.7 | 181.2 | 248.4 | 134.9 | 243.1 | 214.0 | 135.4 |
| **Acridone** | + | -68.3 | -72.8 | -71.7 | -71.5 | -57.1 | -40.7 | -64.8 | -70.0 | -53.8 | -45.8 |
| **Benzotriazole** | - | -52.8 | -54.6 | -34.0 | -18.9 | -17.6 | 153.0 | 74.1 | 42.6 | -0.1 | -23.3 |
| **5-Methyl-2H-Benzotriazole** | + | -34.8 | -64.7 | -60.3 | -48.0 | -37.1 | - | - | - | - | - |
| **Bezafibrate** | - | -2.8 | -22.5 | -13.8 | 15.7 | -28.8 | -12.2 | -19.0 | -5.6 | 7.7 | -18.1 |
| **Bisphenol A** | - | -2.7 | -15.9 | 2.5 | -3.0 | -16.0 | 51.0 | 20.6 | 18.9 | 17.6 | -5.6 |
| **Caffeine** | + | 47.5 | 16.6 | -1.4 | 1.4 | -8.7 | 85.7 | 40.3 | 15.6 | 11.3 | -9.4 |
| **Carbamazepine** | + | -5.0 | -13.9 | -17.0 | -9.3 | -11.2 | -2.0 | -13.1 | -7.5 | -5.2 | -4.2 |
| **Carbamazepine-10,11-epoxide** | + | -45.1 | -52.4 | -30.6 | -86.5 | -82.8 | -45.6 | -66.5 | -52.7 | -87.7 | -82.7 |
| **Chloramphenicol** | - | -23.6 | -15.1 | -22.6 | -5.5 | -18.8 | 69.0 | 38.7 | 29.9 | 13.2 | 2.2 |
| **Ciprofloxacin** | + | -96.0 | -95.2 | -96.5 | -93.4 | -92.1 | -45.1 | -85.6 | -86.0 | -80.4 | -81.9 |
| **Citalopram** | + | -89.6 | -93.8 | -89.5 | -92.3 | -86.0 | -84.0 | -90.3 | -88.8 | -88.9 | -82.7 |
| **Clarithromycin** | + | -72.1 | -83.5 | -65.2 | -78.6 | -73.8 | -66.4 | -77.3 | -63.5 | -65.2 | -56.4 |
| **Climbazole** | - | -65.5 | -80.8 | -77.0 | -82.2 | -77.8 | -66.7 | -78.8 | -75.6 | -80.5 | -78.9 |
| **Clofibric acid** | - | 189.2 | 196.9 | 139.7 | 182.6 | 102.9 | 13.0 | -1.7 | 6.1 | 29.5 | 5.9 |
| **Diclofenac** | - | -79.5 | -79.5 | -81.8 | -84.6 | -82.8 | -78.4 | -85.1 | -83.3 | -78.5 | -73.9 |
| **4-Hydroxydiclofenac** | + | -42.5 | -45.0 | -51.9 | -58.4 | -58.2 | 5.7 | 25.0 | 1.4 | -1.9 | -54.0 |
| **Diltiazem** | + | 146.5 | -47.8 | 185.0 | -83.6 | -69.7 | 136.6 | -18.5 | 190.4 | -75.7 | -60.5 |
| **Fenofibrate** | + | -92.3 | -96.0 | -87.8 | -96.5 | -95.4 | -91.1 | -95.1 | -94.9 | -93.8 | -89.4 |
| **Fipronil** | - | -46.4 | -41.3 | -43.7 | -38.5 | -50.5 | -33.3 | -41.7 | -43.5 | -38.8 | -51.2 |
| **Fipronil desulfinyl** | - | -89.9 | -92.5 | -88.9 | -85.0 | -84.1 | -88.2 | -92.2 | -89.4 | -84.9 | -84.3 |
| **Fipronil sulfone** | - | -76.7 | -76.8 | -73.9 | -62.4 | -69.1 | -71.2 | -76.8 | -74.0 | -62.6 | -70.2 |
| **Fluconazole** | + | 30.5 | -21.2 | -14.7 | -6.7 | -8.7 | 5.6 | 2.0 | -4.8 | 2.0 | -0.8 |
| **Furosemide** | - | -26.9 | -54.0 | -44.0 | -24.1 | -46.8 | 172.9 | 53.7 | 10.6 | -17.0 | -38.1 |
| **Gemfibrozil** | - | -75.4 | -82.1 | -81.3 | -81.3 | -75.2 | -91.6 | -84.3 | -88.7 | -80.8 | -79.7 |
| **Hydrochlorothiazide** | - | -27.2 | -46.1 | -31.8 | -20.5 | -38.6 | 35.9 | -8.4 | -9.2 | -11.6 | -15.8 |
| **Ibuprofen** | - | -25.5 | -54.5 | -42.6 | -47.3 | -53.0 | -39.2 | -34.7 | -59.1 | -19.5 | -37.8 |
| **Indomethacine** | - | 194.5 | 42.2 | 129.2 | -82.1 | -87.7 | - | - | - | - | - |
| **Irbesartan** | - | -89.5 | -91.9 | -88.9 | -93.5 | -93.2 | -86.7 | -91.6 | -91.6 | -89.9 | -79.1 |
| **Lamotrigine** | + | -79.8 | -80.4 | -76.4 | -72.8 | -67.5 | -77.4 | -77.5 | -74.8 | -75.1 | -72.5 |
| **Lamotrigine N2-oxide** | + | -63.3 | -64.0 | -57.3 | -19.1 | -3.6 | -45.2 | -46.5 | -53.2 | -10.6 | -18.2 |
| **5-Desamino 5-Oxo-2,5-dihydro-Lamotrigine** | + | 57.8 | -26.3 | -16.1 | -14.2 | -15.8 | -23.5 | -15.9 | -10.2 | -8.2 | -15.9 |
| **N2-Methyl-Lamotrigine** | + | -73.0 | -77.9 | -78.6 | -75.1 | -76.7 | 30.2 | -44.7 | -36.9 | -54.8 | -50.9 |
| **Metoprolol** | + | -16.7 | -1.9 | 16.3 | -2.1 | -18.8 | -9.9 | -9.3 | -4.9 | -2.2 | -20.6 |
| **Metrodinazole** | + | 58.4 | 5.0 | 3.7 | -4.5 | -13.3 | 101.7 | 140.5 | 98.9 | 0.9 | -9.9 |
| **Oxcarbazepine** | + | 84.3 | 150.1 | 66.8 | 142.8 | 11.4 | 133.4 | 190.8 | 113.8 | 131.0 | 22.8 |
| **Propranolol** | + | -89.1 | -89.0 | -78.9 | -80.3 | -81.8 | 126.5 | 103.5 | 179.5 | -17.4 | -64.9 |
| **Sucralose** | - | 216.7 | 132.3 | 211.4 | 71.0 | 30.1 | 258.2 | 135.9 | 213.9 | 69.8 | 25.7 |
| **Sulfamethazine** | + | -66.5 | -80.8 | -76.5 | -78.2 | -74.3 | -24.4 | -40.9 | -54.5 | -71.8 | -60.7 |
| **Sulfamethoxazole** | - | -76.6 | -78.3 | -74.6 | -73.9 | -67.6 | -71.2 | -69.8 | -69.2 | -60.9 | -48.9 |
| **4-Nitro-Sulfamethoxazole** | + | -26.7 | -24.5 | -27.2 | -1.6 | -19.5 | 96.6 | 68.2 | 63.0 | 68.1 | 24.7 |
| **N4-Acetylsulfamethoxazole** | - | -30.8 | -39.3 | -40.1 | -16.1 | -37.4 | 158.3 | 145.0 | 102.6 | 123.4 | 28.0 |
| **Sulfanilamide** | - | -67.6 | -62.6 | -67.8 | -65.6 | -51.7 | -67.3 | -74.7 | -70.3 | -66.1 | -53.4 |
| **Sulfanilic acid** | - | -59.4 | -47.3 | -46.9 | -65.2 | -69.3 | -64.0 | -51.5 | -56.9 | -63.6 | -65.0 |
| **Valsartan** | - | -21.3 | -42.0 | -39.8 | -57.4 | -62.7 | -18.9 | -54.1 | -56.8 | -59.7 | -64.8 |
| **Valsartan acid** | + | 22.4 | 35.7 | 42.1 | 33.6 | 12.7 | -38.3 | -61.4 | -61.7 | -42.0 | -48.7 |
| **Verapamil** | + | -83.0 | -94.2 | -87.9 | -94.9 | -92.0 | -79.0 | -89.5 | -88.1 | -89.5 | -83.1 |

## Table S7. Comparison of Matrix effect (%) for target analytes in MRM^HR^ and SWATH acquisition modes in soil matrix

| **Compound** | **ESI** | **MRM^HR^ acquisition mode** | | | | | **SWATH acquisition mode** | | | | |
| --- | --- | --- | --- | --- | --- | --- | --- | --- | --- | --- | --- |
|  |  | **2 µg L^-1^** | **5 µg L^-1^** | **10 µg L^-1^** | **50 µg L^-1^** | **200 µg L^-1^** | **2 µg L^-1^** | **5 µg L^-1^** | **10 µg L^-1^** | **50 µg L^-1^** | **200 µg L^-1^** |
| **Acesulfame** | - | 40.0 | 26.0 | 32.2 | 47.7 | 12.4 | 97.3 | 62.6 | 44.3 | 26.1 | 7.5 |
| **Acetaminophen** | - | 147.5 | 119.1 | 143.5 | 115.4 | 172.9 | 141.5 | 158.3 | 126.8 | 158.4 | 107.7 |
| **Acridone** | + | 27.3 | -4.3 | 3.3 | -1.7 | -7.7 | -42.1 | -61.2 | -64.3 | -50.0 | -43.3 |
| **Benzotriazole** | - | 85.4 | 43.8 | 18.5 | 6.0 | -7.6 | -25.7 | 15.9 | 55.5 | 4.3 | -30.0 |
| **5-Methyl-2H-Benzotriazole** | + | 16.3 | -3.4 | 1.4 | 3.7 | -3.6 | -30.1 | -61.3 | -51.7 | -41.4 | -29.5 |
| **Bezafibrate** | - | 13.3 | 38.3 | -1.9 | 32.1 | -10.8 | -8.0 | -16.7 | -1.3 | 11.4 | -17.3 |
| **Bisphenol A** | - | 1.4 | 13.0 | -12.6 | -9.3 | -24.1 | 45.1 | 20.5 | 17.1 | 16.0 | -8.4 |
| **Caffeine** | + | 163.8 | 134.6 | 155.3 | 12.3 | -8.0 | 131.1 | 47.1 | 26.1 | 1.4 | -5.2 |
| **Carbamazepine** | + | 45.7 | 18.7 | 33.5 | -0.6 | 4.0 | 3.4 | -11.1 | -2.5 | -5.0 | -4.9 |
| **Carbamazepine-10,11-epoxide** | + | 66.7 | 39.2 | 127.0 | 9.2 | -3.3 | -62.0 | -67.2 | -59.5 | -87.8 | -80.8 |
| **Chloramphenicol** | - | 0.0 | -12.5 | -18.2 | 6.5 | -9.1 | 67.1 | 30.5 | 23.6 | 13.1 | 2.5 |
| **Ciprofloxacin** | + | 69.2 | 9.4 | -6.4 | -34.3 | -33.0 | -34.5 | -88.2 | -89.2 | -87.0 | -84.2 |
| **Citalopram** | + | -47.6 | -65.6 | -50.7 | -61.1 | -32.0 | -81.5 | -89.5 | -88.3 | -88.6 | -81.2 |
| **Clarithromycin** | + | 50.1 | 14.8 | 114.3 | 38.5 | 14.7 | -65.3 | -76.2 | -62.7 | -64.8 | -55.4 |
| **Climbazole** | - | -64.7 | -77.6 | -75.6 | -78.4 | -74.7 | -64.6 | -77.8 | -70.3 | -80.6 | -78.6 |
| **Clofibric acid** | - | 171.7 | 183.5 | 132.2 | 182.6 | 95.4 | 13.7 | 4.6 | 6.3 | 21.0 | 8.9 |
| **Diclofenac** | - | 11.8 | 16.6 | 33.2 | 21.2 | -6.1 | -30.1 | -51.8 | -54.9 | -65.2 | -66.8 |
| **4-Hydroxydiclofenac** | + | -1.1 | 24.3 | 57.5 | 34.3 | 7.3 | 26.1 | 10.6 | -24.1 | -51.5 | -56.5 |
| **Diltiazem** | + | 121.2 | 178.2 | 119.8 | -7.3 | -13.2 | 167.2 | 137 | 151.3 | -74.4 | -57.9 |
| **Fenofibrate** | + | -84.6 | -91.5 | -83.0 | -84.6 | -76.6 | -89.5 | -94.8 | -92.8 | -93.5 | -89.2 |
| **Fipronil** | - | -33.2 | -39.7 | -44.8 | -38.8 | -48.7 | -29.9 | -37.2 | -39.0 | -38.5 | -50.6 |
| **Fipronil desulfinyl** | - | -24.4 | -21.7 | -23.0 | -20.3 | -26.6 | -17.5 | -21.9 | -22.9 | -20.4 | -26.9 |
| **Fipronil sulfone** | - | -47.3 | -48.7 | -46.8 | -44.7 | -44.3 | -46.4 | -48.5 | -47.1 | -44.7 | -44.4 |
| **Fluconazole** | + | 22.3 | -10.9 | 20.5 | 1.4 | -8.5 | 55.5 | 8.4 | 6.0 | 17.4 | 0.1 |
| **Furosemide** | - | 66.2 | -21.1 | -30.5 | -7.1 | -34.4 | 128.5 | 39.9 | -9.5 | -13.7 | -35.0 |
| **Gemfibrozil** | - | -77.4 | -81.5 | -81.9 | -75.6 | -74.3 | -85.5 | -90.7 | -86.2 | -78.6 | -79.1 |
| **Hydrochlorothiazide** | - | -30.4 | -33.4 | -36.9 | -23.2 | -34.5 | 48.9 | 0.3 | -2.7 | -11.8 | -15.5 |
| **Ibuprofen** | - | -16.7 | -52.5 | -34.4 | -17.7 | -49.3 | -16.8 | 50.9 | -15.5 | -25.2 | -32.6 |
| **Indomethacine** | - | 151.6 | 3.2 | 132.3 | -79.1 | -85.3 | - | - | - | - | - |
| **Irbesartan** | - | 5.1 | -6.3 | 4.7 | 0.3 | -2.2 | -71.9 | -87.6 | -90.5 | -87.8 | -75.0 |
| **Lamotrigine** | + | -25.4 | -37.9 | -28.7 | -25.7 | -31.7 | -70.7 | -78.8 | -74.6 | -74.8 | -71.8 |
| **Lamotrigine N2-oxide** | + | 10.8 | -0.7 | 39.5 | 48.2 | 38.3 | -26.9 | -55.3 | -50.6 | -17.1 | -21.7 |
| **5-Desamino 5-Oxo-2,5-dihydro-Lamotrigine** | + | 18.1 | 22.8 | 8.7 | -8.7 | -16.8 | -16.9 | -21.8 | -24.4 | -12.5 | -19.1 |
| **N2-Methyl-Lamotrigine** | + | -13.0 | -30.6 | -4.5 | -24.3 | -29.5 | 271.2 | 108.3 | 18.7 | -34.8 | -37.7 |
| **Metoprolol** | + | 44.9 | 20.0 | 32.1 | 10.2 | -8.3 | 52.4 | 14.4 | 19.7 | 15.0 | -11.7 |
| **Metrodinazole** | + | 181.4 | 57.9 | 93.0 | 3.4 | -5.6 | 174.0 | 42.6 | 37.5 | -13.4 | -15.9 |
| **Oxcarbazepine** | + | 111.2 | 140.0 | 101.3 | 97.0 | 3.0 | -57.5 | -67.3 | -58.9 | -87.9 | -80.7 |
| **Propranolol** | + | -36.9 | -40.3 | -33.2 | -47.1 | -51.6 | 132.0 | 118.0 | 87.1 | -47.1 | -66.4 |
| **Sucralose** | - | 95.2 | 17.6 | 136.7 | 21.3 | -10.8 | 238.5 | 155.0 | 237.2 | 75.2 | 31.2 |
| **Sulfamethazine** | + | 5.7 | -12.1 | 2.2 | 2.0 | -4.2 | -8.0 | -46.3 | -55.3 | -68.1 | -55.6 |
| **Sulfamethoxazole** | - | 0.2 | -11.4 | 11.0 | 9.2 | -1.1 | -79.6 | -78.8 | -77.6 | -72.5 | -61.1 |
| **4-Nitro-Sulfamethoxazole** | + | 48.2 | 14.1 | 28.6 | 7.1 | 4.1 | 79.5 | 61.1 | 55.5 | 68.0 | 27.9 |
| **N4-Acetylsulfamethoxazole** | - | 68.1 | 18.0 | 27.8 | 32.0 | -4.9 | 107.0 | 76.1 | 72.0 | 60.6 | 25.3 |
| **Sulfanilamide** | - | -12.7 | -65.8 | -58.9 | -58.2 | -51.2 | -63.9 | -69.7 | -67.6 | -65.9 | -51.9 |
| **Sulfanilic acid** | - | -56.4 | -47.1 | -50.9 | -64.1 | -69.3 | -63.0 | -48.5 | -55.7 | -65.8 | -67.1 |
| **Valsartan** | - | 39.9 | 34.1 | 59.9 | 55.6 | 15.8 | 115.3 | 26.2 | 10.4 | -39.6 | -46.3 |
| **Valsartan acid** | + | 28.4 | -2.5 | 26.5 | 7.5 | -3.0 | -31.2 | -50.4 | -54.9 | -48.7 | -47.5 |
| **Verapamil** | + | -49.3 | -69.2 | -60.1 | -60.2 | -40.0 | -83.8 | -90.6 | -88.5 | -88.7 | -82.8 |

## Table S8. Relative recoveries and intraday precision (RSD%) at 5 concentrations (2, 5, 10, 50, 200 µg L^-1^) with MRM^HR^ and SWATH acquisition modes for lettuce root matrix

| **compound** | **ESI** | **MRM^HR^ acquisition mode** | | | | | | | | | | | **SWATH acquisition mode** | | | | | | | | | | |
| --- | --- | --- | --- | --- | --- | --- | --- | --- | --- | --- | --- | --- | --- | --- | --- | --- | --- | --- | --- | --- | --- | --- | --- |
|  |  | **2**  **µg L^-1^** | **RSD (%)** | **5**  **µg L^-1^** | **RSD (%)** | **10**  **µg L^-1^** | **RSD (%)** | **50**  **µg L^-1^** | **RSD (%)** | **200**  **µg L^-1^** | **RSD (%)** | **2**  **µg L^-1^** | | **RSD (%)** | **5**  **µg L^-1^** | **RSD (%)** | **10**  **µg L^-1^** | **RSD (%)** | **50**  **µg L^-1^** | **RSD (%)** | **200**  **µg L^-1^** | **RSD (%)** |  |
| **Acesulfame** | - | 73.7 | 10.3 | 88.2 | 5.2 | 72.8 | 4.7 | 71.2 | 4.8 | 75.4 | 4.5 | 72.3 | | 15.6 | 72.2 | 5.8 | 88.8 | 10.5 | 72.1 | 3.0 | 80.9 | 2.6 |  |
| **Acetaminophen** | - | 88.4 | 15.5 | 93.9 | 15.3 | 82.9 | 5.6 | 65.7 | 3.7 | 89.2 | 10.2 | 84.0 | | 6.1 | 89.0 | 1.8 | 92.6 | 3.4 | 78.1 | 3.0 | 82.5 | 3.0 |  |
| **Acridone** | + | 69.0 | 5.1 | 74.3 | 0.7 | 65.5 | 10.8 | 66.8 | 5.6 | 81.6 | 14.8 | 67.2 | | 7.4 | 78.2 | 5.4 | 72.5 | 2.9 | 73.3 | 7.3 | 78.2 | 5.5 |  |
| **Benzotriazole** | - | 73.6 | 9.3 | 99.9 | 20.0 | 72.5 | 9.4 | 58.9 | 3.9 | 67.2 | 8.3 | 114.6 | | 10.9 | 128.7 | 9.6 | 77.1 | 9.1 | 74.6 | 4.2 | 65.9 | 15.3 |  |
| **5-Methyl-2H-Benzotriazole** | + | 54.1 | 11.1 | 111.0 | 17.9 | 80.9 | 12.0 | 63.7 | 11.9 | 69.7 | 8.0 | nd | |  | nd |  | nd |  | nd |  | nd |  |  |
| **Bezafibrate** | - | 86.1 | 11.3 | 92.4 | 17.8 | 102.4 | 13.2 | 83.9 | 8.0 | 104.1 | 2.7 | 74.8 | | 9.3 | 103.1 | 16.3 | 95.7 | 1.1 | 85.0 | 2.8 | 90.7 | 6.2 |  |
| **Bisphenol A** | - | 109.8 | 7.6 | 129.8 | 5.8 | 103.0 | 3.3 | 82.5 | 7.9 | 86.6 | 7.5 | 84.1 | | 7.8 | 98.9 | 1.0 | 91.7 | 1.4 | 81.1 | 2.2 | 88.2 | 1.4 |  |
| **Caffeine** | + | 108.1 | 53.8 | 112.6 | 20.2 | 82.1 | 8.5 | 62.7 | 5.4 | 68.5 | 5.4 | 103.3 | | 6.5 | 95.8 | 10.6 | 81.8 | 2.9 | 65.0 | 3.9 | 75.3 | 5.1 |  |
| **Carbamazepine** | + | 98.0 | 6.4 | 110.9 | 6.9 | 103.4 | 2.8 | 86.5 | 1.5 | 91.1 | 2.2 | 90.7 | | 1.9 | 103.9 | 2.2 | 93.6 | 3.1 | 84.3 | 2.1 | 93.8 | 4.7 |  |
| **Carbamazepine-10,11-epoxide** | + | 69.6 | 10.4 | 62.4 | 9.4 | 81.2 | 17.5 | 69.4 | 23.8 | 77.7 | 25.3 | 69.3 | | 22.8 | 82.2 | 10.3 | 87.3 | 15.4 | 66.6 | 16.9 | 80.2 | 19.9 |  |
| **Chloramphenicol** | - | 85.7 | 41.9 | 92.6 | 17.1 | 89.2 | 24.5 | 76.1 | 9.5 | 95.6 | 3.3 | 96.9 | | 12.8 | 103.4 | 5.7 | 98.4 | 1.1 | 90.7 | 3.1 | 93.9 | 1.3 |  |
| **Ciprofloxacin** | + | 36.6 | 0.9 | 28.3 | 30.9 | 22.3 | 8.4 | 15.3 | 3.3 | 15.7 | 6.4 | 30.3 | | 14.5 | 39.5 | 1.5 | 24.9 | 3.5 | 13.1 | 7.5 | 15.6 | 9.0 |  |
| **Citalopram** | + | 75.4 | 17.1 | 86.7 | 20.8 | 71.8 | 11.0 | 100.7 | 16.3 | 101.1 | 19.6 | 69.6 | | 10.0 | 85.6 | 3.0 | 98.0 | 12.4 | 89.7 | 3.5 | 93.0 | 18.4 |  |
| **Clarithromycin** | + | 89.2 | 3.5 | 125.2 | 5.2 | 66.8 | 15.8 | 82.4 | 9.9 | 109.3 | 24.3 | 59.9 | | 7.0 | 86.6 | 8.4 | 77.7 | 13.6 | 72.4 | 8.2 | 93.0 | 11.2 |  |
| **Climbazole** | - | 91.8 | 4.7 | 104.6 | 9.8 | 104.4 | 14.8 | 86.7 | 6.8 | 98.6 | 13.9 | 80.0 | | 4.5 | 93.0 | 7.9 | 103.7 | 13.4 | 84.7 | 4.1 | 89.8 | 11.4 |  |
| **Clofibric acid** | - | 102.8 | 18.9 | 105.8 | 10.8 | 102.8 | 3.5 | 84.7 | 5.9 | 89.5 | 2.4 | 103.2 | | 6.1 | 113.6 | 4.6 | 106.4 | 4.2 | 82.4 | 4.6 | 92.7 | 3.9 |  |
| **Diclofenac** | - | 73.5 | 35.2 | 81.3 | 15.5 | 102.9 | 3.2 | 92.8 | 20.8 | 88.8 | 9.0 | 75.3 | | 4.0 | 98.7 | 14.5 | 98.7 | 4.0 | 87.4 | 7.1 | 97.7 | 2.9 |  |
| **4-Hydroxydiclofenac** | + | 93.4 | 35.8 | 74.6 | 22.3 | 86.1 | 8.5 | 106.2 | 2.4 | 95.1 | 13.8 | nd | |  | 46.4 | 2.5 | 54.4 | 23.6 | 42.9 | 11.2 | 104.1 | 18.4 |  |
| **Diltiazem** | + | 47.1 | 3.8 | 94.5 | 10.4 | 75.7 | 16.4 | 85.9 | 14.4 | 89.3 | 20.2 | 71.7 | | 3.1 | 90.0 | 7.2 | 93.9 | 10.2 | 84.8 | 3.8 | 91.9 | 15.1 |  |
| **Fenofibrate** | + | 63.6 | 34.4 | 136.3 | 25.5 | 63.1 | 34.7 | 110.9 | 29.6 | 121.4 | 26.6 | 81.5 | | 0.7 | 100.1 | 9.7 | 117.7 | 19.1 | 96.3 | 7.6 | 100.9 | 23.7 |  |
| **Fipronil** | - | 91.7 | 4.2 | 106.4 | 3.1 | 102.9 | 4.7 | 86.5 | 6.4 | 93.7 | 3.7 | 93.1 | | 0.6 | 106.3 | 1.6 | 101.7 | 4.1 | 86.8 | 2.8 | 94.2 | 3.0 |  |
| **Fipronil desulfinyl** | - | 79.9 | 5.3 | 108.9 | 3.4 | 102.1 | 15.2 | 67.8 | 2.0 | 105.5 | 11.9 | 80.0 | | 1.3 | 107.3 | 3.8 | 107.8 | 16.2 | 68.3 | 4.3 | 99.5 | 9.9 |  |
| **Fipronil sulfone** | - | 94.1 | 6.6 | 102.5 | 8.0 | 102.2 | 10.6 | 66.8 | 5.1 | 94.8 | 2.9 | 86.8 | | 3.8 | 105.8 | 2.2 | 98.1 | 7.5 | 67.4 | 3.5 | 94.2 | 3.4 |  |
| **Fluconazole** | + | 76.2 | 7.0 | 112.3 | 7.2 | 88.3 | 4.8 | 78.1 | 6.3 | 77.9 | 2.3 | 94.1 | | 2.6 | 90.2 | 1.8 | 93.5 | 1.1 | 76.4 | 5.7 | 80.9 | 10.5 |  |
| **Furosemide** | - | 107.9 | 16.8 | 98.4 | 17.3 | 100.0 | 20.7 | 82.2 | 6.8 | 103.0 | 9.0 | 75.7 | | 26.6 | 101.3 | 18.2 | 91.0 | 14.6 | 97.0 | 3.6 | 103.5 | 1.8 |  |
| **Gemfibrozil** | - | 86.4 | 16.4 | 107.9 | 9.5 | 93.4 | 9.7 | 99.1 | 8.8 | 102.6 | 6.5 | 74.7 | | 14.2 | 47.6 | 7.1 | 106.1 | 14.4 | 87.8 | 16.5 | 95.4 | 1.7 |  |
| **Hydrochlorothiazide** | - | 100.3 | 6.1 | 109.7 | 26.2 | 96.0 | 4.5 | 88.2 | 3.6 | 96.1 | 7.0 | 94.4 | | 16.1 | 101.7 | 9.6 | 94.5 | 7.2 | 84.6 | 5.9 | 91.8 | 3.8 |  |
| **Ibuprofen** | - | 111.3 | 25.5 | 136.0 | 6.9 | 107.8 | 21.4 | 90.8 | 6.9 | 98.1 | 3.9 | 77.7 | | 5.6 | 172.7 | 60.7 | 177.9 | 22.1 | 87.4 | 11.4 | 91.8 | 6.2 |  |
| **Indomethacin** | - | 103.3 | 15.0 | 119.4 | 23.3 | 121.0 | 11.2 | 98.6 | 9.8 | 112.4 | 21.9 | nd | |  | nd |  | nd |  | nd |  | nd |  |  |
| **Irbesartan** | - | 72.2 | 24.4 | 89.4 | 22.7 | 89.6 | 37.6 | 94.6 | 9.9 | 124.7 | 33.1 | 92.7 | | 39.2 | 86.4 | 12.2 | 97.1 | 6.4 | 87.9 | 4.7 | 91.7 | 20.0 |  |
| **Lamotrigine** | + | 57.5 | 6.2 | 67.7 | 6.6 | 67.6 | 9.1 | 65.8 | 8.1 | 69.9 | 13.0 | 74.5 | | 32.4 | 99.3 | 6.3 | 73.0 | 6.0 | 63.4 | 7.7 | 74.6 | 14.0 |  |
| **Lamotrigine N2-oxide** | + | 103.5 | 25.6 | 74.8 | 15.1 | 87.2 | 29.6 | 77.9 | 6.6 | 85.4 | 10.4 | 68.0 | | 12.6 | 62.7 | 7.1 | 67.6 | 9.8 | 75.3 | 9.3 | 91.3 | 7.1 |  |
| **5-Desamino 5-Oxo-2,5-dihydro-Lamotrigine** | + | 67.8 | 10.8 | 69.7 | 21.2 | 74.9 | 16.3 | 75.0 | 5.0 | 78.1 | 6.3 | 72.7 | | 12.0 | 88.4 | 4.3 | 78.9 | 2.0 | 68.4 | 8.3 | 72.9 | 3.0 |  |
| **N2-Methyl-Lamotrigine** | + | 73.1 | 15.7 | 91.3 | 12.1 | 89.4 | 23.7 | 76.9 | 8.8 | 79.9 | 14.2 | 81.6 | | 12.3 | 92.8 | 10.4 | 127.3 | 11.5 | 80.3 | 4.9 | 86.1 | 6.3 |  |
| **Metoprolol** | + | 122.9 | 21.2 | 94.9 | 7.2 | 75.6 | 6.0 | 84.5 | 2.0 | 76.8 | 5.1 | 111.4 | | 11.0 | 91.2 | 7.5 | 93.7 | 8.8 | 79.3 | 3.8 | 84.6 | 2.6 |  |
| **Metrodinazole** | + | 95.1 | 11.3 | 90.7 | 7.8 | 96.7 | 4.7 | 70.6 | 3.6 | 86.1 | 2.7 | 101.3 | | 15.8 | 99.6 | 11.7 | 86.8 | 4.4 | 81.0 | 7.3 | 80.0 | 1.4 |  |
| **Oxcarbazepine** | + | 84.8 | 4.3 | 94.7 | 13.9 | 96.7 | 7.1 | 79.0 | 2.1 | 81.8 | 6.8 | 95.7 | | 15.8 | 94.3 | 10.8 | 80.0 | 2.7 | 80.7 | 3.4 | 84.1 | 3.3 |  |
| **Propranolol** | + | 92.4 | 28.4 | 160.8 | 8.9 | 73.9 | 25.1 | 75.5 | 9.8 | 84.1 | 25.7 | 101 | | 33.9 | 55.3 | 24.3 | 58.2 | 26.6 | 55.8 | 28.3 | 86 | 9.7 |  |
| **Sucralose** | - | 84.9 | 14.5 | 96.5 | 6.3 | 90.7 | 3.3 | 75.1 | 1.1 | 83.1 | 3.9 | 88.2 | | 7.7 | 92.0 | 5.1 | 90.9 | 1.3 | 73.8 | 2.9 | 82.3 | 2.2 |  |
| **Sulfamethazine** | + | 17.3 | 10.0 | 10.0 | 5.0 | 17.4 | 10.6 | 14.3 | 1.8 | 12.7 | 2.4 | 23.5 | | 13.6 | 43.2 | 25.3 | 34.6 | 20.2 | 20.4 | 5.3 | 17.3 | 2.9 |  |
| **Sulfamethoxazole** | - | 31.1 | 14.3 | 41.0 | 14.9 | 30.6 | 3.2 | 30.2 | 6.3 | 30.8 | 4.9 | 30.8 | | 12.0 | 37.4 | 10.4 | 38.1 | 2.3 | 30.3 | 2.1 | 32.8 | 4.1 |  |
| **4-Nitro-Sulfamethoxazole** | + | 78.5 | 8.0 | 90.4 | 5.6 | 87.2 | 9.4 | 77.4 | 7.7 | 83.8 | 4.3 | 110.5 | | 36.2 | 97.2 | 2.0 | 99.9 | 9.1 | 82.7 | 3.4 | 88.1 | 4.1 |  |
| **N4-Acetylsulfamethoxazole** | - | 97.9 | 12.0 | 96.9 | 2.1 | 98.8 | 5.4 | 82.9 | 0.5 | 105.2 | 4.7 | 119.7 | | 39.6 | 104.8 | 19.0 | 104.9 | 13.9 | 65.8 | 3.4 | 87.5 | 4.0 |  |
| **Sulfanilamide** | - | nd |  | nd |  | nd |  | 11.1 | 3.3 | 10.5 | 2.3 | 75.1 | | 17.4 | 40.3 | 15.4 | 28.9 | 24.3 | 10.1 | 1.9 | 12.4 | 1.1 |  |
| **Sulfanilic acid** | - | 106.3 | 4.3 | 83.3 | 5.6 | 66.8 | 2.4 | 39.8 | 24.1 | 11.4 | 6.6 | 99.4 | | 4.0 | 78.2 | 5.4 | 63.4 | 1.6 | 33.1 | 3.3 | 9.1 | 1.9 |  |
| **Valsartan** | - | 74.3 | 14.1 | 94.0 | 16.6 | 82.1 | 3.8 | 97.7 | 4.1 | 83.7 | 16.4 | 90.3 | | 7.8 | 95.5 | 25.4 | 100.2 | 8.4 | 78.8 | 0.1 | 74.7 | 17.6 |  |
| **Valsartan acid** | + | 105.9 | 23.4 | 90.2 | 13.3 | 91.9 | 27.1 | 68.6 | 4.4 | 73.4 | 23.4 | 90.8 | | 14.0 | 101.9 | 17.8 | 95.1 | 9.2 | 64.5 | 4.8 | 68.6 | 21.1 |  |
| **Verapamil** | + | 40.8 | 11.2 | 88.8 | 3.9 | 63.0 | 19.8 | 111.3 | 24.3 | 152.0 | 22.2 | 65.8 | | 2.9 | 72.2 | 12.2 | 94.4 | 15.0 | 102.8 | 9.3 | 98.8 | 23.9 |  |

## Table S9. Relative recoveries and intraday precision (RSD%) at 5 concentrations (2, 5, 10, 50, 200 µg L^-1^) with MRM^HR^ and SWATH acquisition modes for soil matrix

| **compound** | **ESI** | **MRM^HR^ acquisition mode** | | | | | | | | | | **SWATH acquisition mode** | | | | | | | | | |
| --- | --- | --- | --- | --- | --- | --- | --- | --- | --- | --- | --- | --- | --- | --- | --- | --- | --- | --- | --- | --- | --- |
|  |  | **2**  **µg L^-1^** | **RSD (%)** | **5**  **µg L^-1^** | **RSD (%)** | **10**  **µg L^-1^** | **RSD (%)** | **50**  **µg L^-1^** | **RSD (%)** | **200 µg L^-1^** | **RSD (%)** | **2**  **µg L^-1^** | **RSD (%)** | **5**  **µg L^-1^** | **RSD (%)** | **10**  **µg L^-1^** | **RSD (%)** | **50**  **µg L^-1^** | **RSD (%)** | **200**  **µg L^-1^** | **RSD (%)** |
| **Acesulfame** | - | 77.9 | 11.0 | 76.3 | 3.9 | 77.6 | 3.9 | 57.5 | 4.6 | 71.7 | 4.2 | 82.1 | 18.6 | 78.6 | 10.2 | 73.9 | 6.4 | 73.8 | 5 | 80 | 4.6 |
| **Acetaminophen** | - | 113.2 | 52.6 | 73.5 | 14.9 | 99.9 | 7.1 | 75.4 | 5.0 | 75.4 | 3.5 | 92.1 | 8.1 | 95.9 | 1.7 | 95 | 9.4 | 78.2 | 2.3 | 83 | 3.3 |
| **Acridone** | + | 68.9 | 5.7 | 83.0 | 12.7 | 55.1 | 2.5 | 63.1 | 6.5 | 81.9 | 4.9 | 93.2 | 13.2 | 86 | 8.8 | 75.5 | 5.9 | 73.9 | 3.9 | 82.5 | 3.8 |
| **Benzotriazole** | - | 56.1 | 10.0 | 63.7 | 17.8 | 59.7 | 15.2 | 56.3 | 4.7 | 63.6 | 5.0 | 76.9 | 13.7 | 10.7 | 3.1 | 90.3 | 12.7 | 72.5 | 4.3 | 85 | 21.5 |
| **5-Methyl-2H-Benzotriazole** | + | 68.4 | 7.2 | 66.2 | 3.5 | 52.6 | 5.6 | 54.8 | 4.7 | 70.0 | 1.4 | 50.7 | 7.1 | 95.2 | 7.1 | 77.4 | 16.1 | 64.5 | 6 | 66.5 | 15.4 |
| **Bezafibrate** | - | 87.7 | 6.0 | 97.1 | 6.0 | 101.4 | 5.1 | 83.1 | 2.6 | 96.5 | 2.3 | 85.4 | 15.1 | 112.7 | 5.9 | 96.2 | 5.4 | 82 | 5.3 | 93.1 | 3 |
| **Bisphenol A** | - | 95.0 | 27.2 | 97.7 | 2.1 | 104.0 | 9.1 | 82.5 | 3.6 | 89.0 | 13.1 | 87.8 | 7.1 | 100.5 | 3.5 | 96.6 | 6.2 | 82.2 | 0.8 | 87.3 | 0.7 |
| **Caffeine** | + | 115.5 | 7.6 | 95.0 | 5.7 | 83.1 | 16.1 | 83.8 | 7.2 | 69.8 | 3.8 | 87.9 | 7.5 | 89 | 5.8 | 76.9 | 3.7 | 72.4 | 3.6 | 74 | 5.5 |
| **Carbamazepine** | + | 76.8 | 7.3 | 90.8 | 4.7 | 72.1 | 6.3 | 93.3 | 2.1 | 92.9 | 1.2 | 92.6 | 1.8 | 98 | 4.8 | 94.5 | 5.4 | 85 | 4.1 | 92.8 | 1.2 |
| **Carbamazepine-10,11-epoxide** | + | 76.9 | 11.1 | 93.7 | 5.6 | 75.3 | 1.8 | 84.0 | 6.5 | 91.8 | 9.5 | 85.5 | 1.2 | 65 | 11.5 | 94.7 | 14.2 | 64.8 | 17.9 | 86.9 | 21.1 |
| **Chloramphenicol** | - | 90.8 | 4.6 | 97.3 | 14.9 | 91.2 | 11.3 | 81.6 | 6.3 | 90.6 | 2.4 | 88.4 | 2.5 | 97.8 | 6.8 | 96.8 | 2.8 | 87 | 3.5 | 91.7 | 0.5 |
| **Ciprofloxacin** | + | 3.2 | 2.2 | 3.6 | 0.5 | 4.8 | 0.2 | 4.3 | 0.1 | 4.4 | 0.1 | 34.7 | 20.1 | 40.7 | 2.4 | 10.1 | 7.7 | 16.6 | 2.4 | 11.9 | 7.5 |
| **Citalopram** | + | 65.7 | 15.5 | 75.2 | 3.7 | 52.8 | 7.4 | 74.1 | 5.5 | 68.3 | 2.0 | 73.5 | 7.3 | 84.5 | 3.9 | 103.3 | 17.3 | 88.5 | 4.9 | 92.3 | 14.4 |
| **Clarithromycin** | + | 86.9 | 8.5 | 93.3 | 8.7 | 72.5 | 5.7 | 81.6 | 3.6 | 86.9 | 1.8 | 57.8 | 6.7 | 83.7 | 6.4 | 80.3 | 14.5 | 76.5 | 8.6 | 94.9 | 8.8 |
| **Climbazole** | - | 79.3 | 14.7 | 85.2 | 8.1 | 88.0 | 12.0 | 90.0 | 4.0 | 87.2 | 10.5 | 68.8 | 5.4 | 82 | 7.9 | 89.8 | 8 | 91.1 | 9.6 | 89.2 | 12.2 |
| **Clofibric acid** | - | 90.0 | 21.9 | 106.2 | 8.7 | 93.1 | 4.3 | 78.6 | 7.3 | 86.0 | 2.9 | 82.9 | 1.5 | 102.8 | 2.1 | 99 | 5.3 | 86.9 | 7.4 | 89.2 | 5.1 |
| **Diclofenac** | - | 87.7 | 8.3 | 79.8 | 2.5 | 65.4 | 5.4 | 81.3 | 12.6 | 89.3 | 6.5 | 56.1 | 12.1 | 54.7 | 16.2 | 102 | 16.5 | 93.4 | 11.9 | 91.4 | 8.9 |
| **4-Hydroxydiclofenac** | + | 33.5 | 20.7 | 26.4 | 5.1 | 24.9 | 4.0 | 27.8 | 1.7 | 31.8 | 4.7 | 66.9 | 29.3 | 99.1 | 25.8 | 85.4 | 25 | 91.6 | 10.1 | 92.2 | 5.7 |
| **Diltiazem** | + | 66.3 | 10.0 | 81.9 | 1.1 | 60.3 | 0.9 | 75.5 | 2.0 | 85.6 | 3.8 | 64.7 | 3.9 | 79.6 | 5.3 | 89.1 | 12.6 | 82.2 | 2.1 | 90 | 14.1 |
| **Fenofibrate** | + | 129.1 | 35.6 | 99 | 20.5 | 80.5 | 29.2 | 91.3 | 5.7 | 80.3 | 6.1 | 79.3 | 25.8 | 97.7 | 15.4 | 91.1 | 18.4 | 91.6 | 14.1 | 104.9 | 15 |
| **Fipronil** | - | 92.2 | 6.0 | 105.7 | 0.7 | 103.3 | 5.7 | 88.4 | 3.0 | 92.9 | 0.9 | 90.1 | 2.2 | 106.3 | 3.2 | 98 | 6.5 | 87.6 | 1.7 | 95.4 | 1.2 |
| **Fipronil desulfinyl** | - | 88.7 | 5.9 | 120.9 | 3.8 | 113.3 | 16.9 | 75.3 | 2.2 | 117.1 | 13.2 | 88.8 | 1.4 | 119.1 | 4.2 | 119.7 | 18.0 | 75.8 | 4.8 | 110.4 | 11.0 |
| **Fipronil sulfone** | - | 104.5 | 7.3 | 113.8 | 8.9 | 113.4 | 11.8 | 74.1 | 5.7 | 105.2 | 3.2 | 96.3 | 4.2 | 117.4 | 2.4 | 108.9 | 8.3 | 74.8 | 3.9 | 104.6 | 3.8 |
| **Fluconazole** | + | 90.7 | 7.8 | 92.7 | 5.6 | 66.8 | 6.5 | 78.3 | 6.8 | 88.1 | 3.3 | 84 | 9.3 | 97 | 1.7 | 86.4 | 1.9 | 75.2 | 6.9 | 78.4 | 9.1 |
| **Furosemide** | - | 94.2 | 18.0 | 105.5 | 12.0 | 99.5 | 12.3 | 79.2 | 4.8 | 96.8 | 4.6 | 112.6 | 31.6 | 97.4 | 14.7 | 98.4 | 18.2 | 77.8 | 7.2 | 99.7 | 2 |
| **Gemfibrozil** | - | 104.8 | 8.5 | 100.5 | 9.7 | 102.6 | 11.9 | 85.1 | 0.3 | 96.2 | 1.5 | 79.1 | 6.3 | 114.4 | 23.2 | 84 | 16 | 88.2 | 4 | 90.2 | 3.8 |
| **Hydrochlorothiazide** | - | 92.9 | 6.7 | 118.2 | 13.4 | 86.5 | 11.1 | 84.2 | 6.4 | 87.9 | 4.9 | 91 | 7.4 | 94.9 | 1 | 94.6 | 11.5 | 85.3 | 7.1 | 92.7 | 3.5 |
| **Ibuprofen** | - | 68.4 | 29.7 | 102.5 | 32.7 | 68.7 | 4.7 | 65.5 | 2.2 | 89.6 | 3.7 | 93.5 | 33 | 53.4 | 18 | 62.9 | 26.7 | 84.8 | 14.4 | 88.7 | 3.9 |
| **Indomethacin** | - | 140.7 | 18.1 | 110.6 | 21.8 | 79.6 | 28 | 92.7 | 6.5 | 94.3 | 16.1 | nd |  | nd |  | nd |  | nd |  | nd |  |
| **Irbesartan** | - | 72.2 | 3.3 | 79 | 5.5 | 68.7 | 7.3 | 83.2 | 2.6 | 88.8 | 3.9 | 99.7 | 28.2 | 101 | 24.8 | 111 | 18.4 | 90.6 | 4.9 | 93 | 16 |
| **Lamotrigine** | + | 53.4 | 3.3 | 57.7 | 1.7 | 50.3 | 1.7 | 59.9 | 1 | 63.4 | 2.3 | 64.3 | 20.5 | 74.8 | 13.1 | 64.5 | 2.3 | 64.1 | 5.5 | 71 | 10.9 |
| **Lamotrigine N2-oxide** | + | 68.1 | 21.2 | 86 | 49.7 | 67.8 | 4.6 | 81.3 | 2.1 | 86.3 | 2.5 | 38.5 | 23.5 | 76.1 | 9 | 66.9 | 4 | 83.7 | 9 | 92 | 9.7 |
| **5-Desamino 5-Oxo-2,5-dihydro-Lamotrigine** | + | 61.4 | 30.7 | 85.2 | 17 | 61.9 | 6.1 | 78.1 | 16.4 | 78.4 | 5.7 | 77.4 | 18.9 | 85.5 | 4.8 | 75.5 | 5.8 | 68.7 | 0.3 | 74.4 | 1.7 |
| **N2-Methyl-Lamotrigine** | + | 58.7 | 3.8 | 82.4 | 6.6 | 53.2 | 4.1 | 73.2 | 2.2 | 71.4 | 3.8 | 116.1 | 37.2 | 80.8 | 11.3 | 98.5 | 22.2 | 77 | 5 | 91.3 | 8.3 |
| **Metoprolol** | + | 63 | 13.8 | 85.5 | 5.9 | 67.9 | 11.6 | 74.4 | 5.4 | 87.9 | 9.4 | 104 | 10.7 | 96.9 | 9.6 | 86.4 | 5.4 | 77.9 | 2.5 | 84 | 5.3 |
| **Metrodinazole** | + | 53.8 | 12.6 | 89.4 | 13 | 65.2 | 12.7 | 76.9 | 3.9 | 84.6 | 6.2 | 92.3 | 12.5 | 101.7 | 9.1 | 84.4 | 3.3 | 69.9 | 3.9 | 82.8 | 1 |
| **Oxcarbazepine** | + | 4.5 | 2.6 | 4.1 | 1.7 | 6.6 | 2.1 | 4 | 0.4 | 5.2 | 0.8 | 80.2 | 12.9 | 66.9 | 10.7 | 94.8 | 14.9 | 65.6 | 18.1 | 84.9 | 24.6 |
| **Propranolol** | + | 73.5 | 14.3 | 68.4 | 11 | 56.2 | 7.6 | 62.1 | 3.9 | 66.3 | 2.5 | 135 | 29.7 | 133.1 | 25.9 | 79.7 | 19.5 | 85.5 | 13.6 | 88.7 | 18.9 |
| **Sucralose** | - | 77.8 | 25 | 69.7 | 32.4 | 79.9 | 19.7 | 70.3 | 2 | 84.8 | 5.1 | 95.6 | 3.9 | 97.2 | 4.5 | 90.6 | 1.7 | 75.1 | 3.1 | 80.6 | 1.4 |
| **Sulfamethazine** | + | 23.1 | 4.6 | 42.5 | 4.1 | 36.1 | 1.6 | 29.5 | 3.5 | 37.7 | 3.6 | 58.9 | 5 | 32.8 | 21.3 | 32.1 | 12.7 | 22.7 | 2.4 | 18.4 | 3.4 |
| **Sulfamethoxazole** | - | 54.6 | 7.9 | 63.4 | 6.8 | 45.8 | 6.7 | 49 | 2 | 56.2 | 3.5 | 34.6 | 24.8 | 38.7 | 10.2 | 35.8 | 0.5 | 34.6 | 3 | 32.8 | 4.2 |
| **4-Nitro-Sulfamethoxazole** | + | 79.2 | 3.9 | 84.5 | 7.5 | 77.4 | 5.3 | 87.3 | 2.1 | 86.6 | 1.1 | 87.7 | 14.3 | 108.1 | 4.3 | 95.8 | 2.7 | 81.6 | 4.9 | 88 | 2.9 |
| **N4-Acetylsulfamethoxazole** | - | 56.8 | 14.8 | 66.9 | 39.5 | 71.4 | 6.8 | 71.9 | 2.7 | 91.7 | 1.8 | 134.7 | 42.2 | 111.7 | 14.4 | 91.6 | 0.9 | 84.2 | 4.4 | 89.3 | 2.8 |
| **Sulfanilamide** | - | 48.7 | 21.9 | 27.8 | 16.3 | 16.7 | 8.8 | 11.6 | 6.2 | 13.8 | 3.6 | 60.3 | 37.3 | 32.5 | 10.4 | 18.9 | 7.4 | 8.7 | 4.4 | 10.8 | 1.4 |
| **Sulfanilic acid** | - | 85 | 6 | 79.9 | 8.3 | 64 | 5.5 | 35 | 7.4 | 9.8 | 1.5 | 95.5 | 12.3 | 73.7 | 2.2 | 58.9 | 4.5 | 33.9 | 5.8 | 9.8 | 2.6 |
| **Valsartan** | - | 98.4 | 8.2 | 88.9 | 6.7 | 79.2 | 4.2 | 79.9 | 2.5 | 86.2 | 9.4 | 131 | 30.8 | 77.3 | 27.8 | 64 | 7 | 84.3 | 8.6 | 74.8 | 16.9 |
| **Valsartan acid** | + | 43.3 | 23.2 | 74.6 | 12.9 | 70.4 | 7.9 | 71.6 | 8.1 | 82.4 | 5.5 | 77.5 | 18.9 | 79.7 | 4.3 | 86.8 | 8.3 | 75.6 | 4.7 | 67.8 | 20.3 |
| **Verapamil** | + | 78.3 | 25.3 | 84.1 | 2 | 72.6 | 12.9 | 76.5 | 2.6 | 73.8 | 6.5 | 44.6 | 30.8 | 79.7 | 7.1 | 99.6 | 21.3 | 95.4 | 12 | 99.5 | 26.8 |

## Table S10. Linearity of the instrumental response (Linearity), MDL and MQL with MRM^HR^ and SWATH acquisition modes for lettuce root matrix

| **Compound** | **ESI** | **MRM^HR^ acquisition mode** | | | | **SWATH acquisition mode** | | | |
| --- | --- | --- | --- | --- | --- | --- | --- | --- | --- |
|  |  | **Linearity**  **(ng g^-1^)** | **r^2^** | **MDL**  **(ng g^-1^)** | **MQL**  **(ng g^-1^)** | **Linearity**  **(ng g^-1^)** | **r^2^** | **MDL**  **(ng g^-1^)** | **MQL**  **(ng g^-1^)** |
| **Acesulfame** | - | 2.5 - 3000 | 0.99653 | 0.04 | 0.12 | 1 - 2000 | 0.99747 | 0.07 | 0.22 |
| **Acetaminophen** | - | 5 - 3000 | 0.99512 | 0.03 | 0.09 | 10 - 2000 | 0.99457 | 0.09 | 0.29 |
| **Acridone** | + | 2.5 - 3000 | 0.99604 | 0.05 | 0.16 | 1 - 2000 | 0.99405 | 0.05 | 0.15 |
| **Benzotriazole** | - | 50 - 2000 | 0.99035 | 0.12 | 0.35 | 10 - 3000 | 0.99581 | 0.16 | 0.49 |
| **5-Methyl-2H-Benzotriazole** | + | 10 - 2000 | 0.99848 | 0.11 | 0.32 | - | - | - | - |
| **Bezafibrate** | - | 2.5 - 3000 | 0.99624 | 0.09 | 0.28 | 5 - 1000 | 0.99640 | 0.10 | 0.30 |
| **Bisphenol A** | - | 2.5 - 3000 | 0.99320 | 0.17 | 0.52 | 0.5 - 2000 | 0.99457 | 0.05 | 0.15 |
| **Caffeine** | + | 5 - 3000 | 0.99812 | 0.10 | 0.29 | 5 - 2000 | 0.99743 | 0.09 | 0.28 |
| **Carbamazepine** | + | 2.5 - 1000 | 0.99763 | 0.04 | 0.11 | 0.5 - 500 | 0.99183 | 0.06 | 0.19 |
| **Carbamazepine-10,11-epoxide** | + | 2.5 - 2000 | 0.99968 | 0.15 | 0.45 | 0.5 - 2000 | 0.99735 | 0.06 | 0.18 |
| **Chloramphenicol** | - | 1 - 2000 | 0.99211 | 0.09 | 0.27 | 2.5 - 2000 | 0.99534 | 0.07 | 0.20 |
| **Ciprofloxacin** | + | 50 - 2000 | 0.99347 | 0.14 | 0.41 | 2.5 -3000 | 0.99296 | 0.02 | 0.06 |
| **Citalopram** | + | 5 - 3000 | 0.99691 | 0.04 | 0.11 | 1 - 3000 | 0.99011 | 0.05 | 0.15 |
| **Clarithromycin** | + | 2.5 - 3000 | 0.99605 | 0.09 | 0.27 | 0.5 - 500 | 0.99602 | 0.02 | 0.05 |
| **Climbazole** | - | 1 - 3000 | 0.99509 | 0.12 | 0.36 | 0.5 - 1000 | 0.99403 | 0.07 | 0.21 |
| **Clofibric acid** | - | 5 - 3000 | 0.99290 | 0.08 | 0.25 | 50 - 3000 | 0.99381 | 0.19 | 0.58 |
| **Diclofenac** | - | 50 - 2000 | 0.99405 | 0.14 | 0.42 | 1 - 3000 | 0.99011 | 0.09 | 0.26 |
| **4-Hydroxydiclofenac** | + | 50 - 2000 | 0.99720 | 0.30 | 0.92 | 2.5 - 2000 | 0.99223 | 0.14 | 0.43 |
| **Diltiazem** | + | 2.5 - 2000 | 0.99767 | 0.13 | 0.38 | 0.5 - 100 | 0.99752 | 0.01 | 0.04 |
| **Fenofibrate** | + | 10 - 2000 | 0.99964 | 0.05 | 0.14 | 0.5 - 3000 | 0.99538 | 0.09 | 0.28 |
| **Fipronil** | - | 0.5 - 100 | 0.98953 | 0.02 | 0.06 | 0.5 - 100 | 0.99618 | 0.02 | 0.06 |
| **Fipronil desulfinyl** | - | 0.5 - 100 | 0.98276 | 0.01 | 0.03 | 0.5 - 100 | 0.99128 | 0.01 | 0.03 |
| **Fipronil sulfone** | - | 1 - 100 | 0.98235 | 0.01 | 0.04 | 0.5 - 100 | 0.98777 | 0.02 | 0.05 |
| **Fluconazole** | + | 2.5 - 3000 | 0.99368 | 0.03 | 0.10 | 0.5 - 2000 | 0.99368 | 0.1 | 0.31 |
| **Furosemide** | - | 5 - 3000 | 0.98989 | 0.14 | 0.41 | 2.5 - 2000 | 0.99982 | 0.06 | 0.17 |
| **Gemfibrozil** | - | 5 - 1000 | 0.95746 | 0.16 | 0.48 | 1 - 3000 | 0.98928 | 0.05 | 0.15 |
| **Hydrochlorothiazide** | - | 2.5 - 100 | 0.99254 | 0.03 | 0.10 | 10 - 3000 | 0.99008 | 0.23 | 0.70 |
| **Ibuprofen** | - | 10 - 3000 | 0.99734 | 0.1 | 0.30 | - | - | - | - |
| **Indomethacin** | - | 2.5 - 3000 | 0.99330 | 0.10 | 0.30 | - | - | - | - |
| **Irbesartan** | - | 10 - 1000 | 0.96436 | 0.21 | 0.64 | 1 - 3000 | 0.99610 | 0.08 | 0.26 |
| **Lamotrigine** | + | 1 - 2000 | 0.99874 | 0.01 | 0.04 | 1 - 1000 | 0.99411 | 0.03 | 0.10 |
| **Lamotrigine N2-oxide** | + | 10 - 2000 | 0.99448 | 0.09 | 0.26 | 2.5 - 100 | 0.99440 | 0.03 | 0.09 |
| **5-Desamino 5-Oxo-2,5-dihydro-Lamotrigine** | + | 10 - 1000 | 0.99803 | 0.12 | 0.38 | 5 - 2000 | 0.99138 | 0.26 | 0.78 |
| **N2-Methyl-Lamotrigine** | + | 5 - 3000 | 0.99813 | 0.05 | 0.16 | 10 - 500 | 0.97921 | 0.01 | 0.04 |
| **Metoprolol** | + | 5 - 3000 | 0.99622 | 0.08 | 0.23 | 2.5 - 300 | 0.99800 | 0.05 | 0.15 |
| **Metrodinazole** | + | 5 - 3000 | 0.99621 | 0.03 | 0.09 | 2.5 - 3000 | 0.99891 | 0.26 | 0.80 |
| **Oxcarbazepine** | + | 2.5 - 2000 | 0.99947 | 0.05 | 0.14 | 0.5 - 2000 | 0.99452 | 0.13 | 0.39 |
| **Propranolol** | + | 2.5 - 2000 | 0.99784 | 0.06 | 0.19 | 5 - 3000 | 0.99623 | 0.03 | 0.08 |
| **Sucralose** | - | 50 - 3000 | 0.96886 | 0.23 | 0.68 | 50 - 3000 | 0.99981 | 0.24 | 0.72 |
| **Sulfamethazine** | + | 50 - 2000 | 0.99910 | 0.12 | 0.37 | 2.5 - 2000 | 0.99920 | 0.19 | 0.57 |
| **Sulfamethoxazole** | - | 50 - 3000 | 0.99376 | 0.08 | 0.23 | 50 - 3000 | 0.99859 | 0.27 | 0.82 |
| **4-Nitro-Sulfamethoxazole** | + | 10 -2000 | 0.99874 | 0.14 | 0.43 | 10 - 3000 | 0.99777 | 0.09 | 0.28 |
| **N4-Acetylsulfamethoxazole** | - | 1 - 3000 | 0.99754 | 0.07 | 0.20 | 1 - 3000 | 0.99570 | 0.07 | 0.21 |
| **Sulfanilamide** | - | 50 - 2000 | 0.99908 | 0.09 | 0.27 | 10 - 2000 | 0.99136 | 0.07 | 0.20 |
| **Sulfanilic acid** | - | 2.5 - 3000 | 0.99680 | 0.16 | 0.48 | 500 - 3000 | 0.99985 | 0.77 | 2.33 |
| **Valsartan** | - | 50 - 3000 | 0.99364 | 0.27 | 0.83 | 50 - 3000 | 0.99353 | 0.19 | 0.58 |
| **Valsartan acid** | + | 2.5 - 3000 | 0.99860 | 0.12 | 0.37 | 2.5 - 3000 | 0.99643 | 0.04 | 0.11 |
| **Verapamil** | + | 50 -3000 | 0.99509 | 0.08 | 0.25 | 50 - 3000 | 0.99128 | 0.12 | 0.35 |

## Table S11. Linearity of the instrumental response (Linearity), MDL and MQL with MRM^HR^ and SWATH acquisition modes for soil matrix

| **Compound** | **ESI** | **MRM^HR^ acquisition mode** | | | | **SWATH acquisition mode** | | | |
| --- | --- | --- | --- | --- | --- | --- | --- | --- | --- |
|  |  | **Linearity**  **(ng g^-1^)** | **r^2^** | **MDL**  **(ng g^-1^)** | **MQL**  **(ng g^-1^)** | **Linearity**  **(ng g^-1^)** | **r^2^** | **MDL**  **(ng g^-1^)** | **MQL**  **(ng g^-1^)** |
| **Acesulfame** | - | 0.1 - 300 | 0.99615 | 0.01 | 0.03 | 0.1 - 300 | 0.99804 | 0.01 | 0.03 |
| **Acetaminophen** | - | 0.25 - 100 | 0.99497 | 0.01 | 0.03 | 0.25 - 300 | 0.99732 | 0.01 | 0.02 |
| **Acridone** | + | 0.25 - 300 | 0.99849 | 0.11 | 0.33 | 0.25 - 100 | 0.99656 | 0.01 | 0.02 |
| **Benzotriazole** | - | 0.25 - 300 | 0.99406 | 0.14 | 0.44 | 0.25 - 300 | 0.99741 | 0.01 | 0.02 |
| **5-Methyl-2H-Benzotriazole** | + | 0.1 - 100 | 0.99546 | 0.03 | 0.10 | 0.25 - 300 | 0.99744 | 0.01 | 0.02 |
| **Bezafibrate** | - | 0.1 - 100 | 0.99619 | 0.01 | 0.02 | 0.05 - 200 | 0.99437 | 0.02 | 0.05 |
| **Bisphenol A** | - | 1 - 300 | 0.99057 | 0.02 | 0.06 | 0.05 - 100 | 0.99816 | 0.01 | 0.04 |
| **Caffeine** | + | 10 - 300 | 0.98766 | 0.11 | 0.34 | 0.05 - 300 | 0.98996 | 0.01 | 0.02 |
| **Carbamazepine** | + | 0.1 - 100 | 0.99614 | 0.01 | 0.03 | 0.05 - 100 | 0.99283 | 0.01 | 0.03 |
| **Carbamazepine-10,11-epoxide** | + | 0.1 - 300 | 0.99850 | 0.01 | 0.03 | 0.05 - 100 | 0.99135 | 0.02 | 0.05 |
| **Chloramphenicol** | - | 0.5 - 300 | 0.99112 | 0.02 | 0.05 | 0.05 - 50 | 0.99723 | 0.02 | 0.05 |
| **Ciprofloxacin** | + | 0.25 - 100 | 0.98827 | 0.01 | 0.03 | 0.05 - 300 | 0.99772 | 0.01 | 0.02 |
| **Citalopram** | + | 0.1 - 200 | 0.99406 | 0.07 | 0.21 | 0.5 - 100 | 0.99355 | 0.01 | 0.02 |
| **Clarithromycin** | + | 0.05 - 200 | 0.99262 | 0.17 | 0.53 | 0.05 - 100 | 0.99044 | 0.01 | 0.02 |
| **Climbazole** | - | 0.5 - 300 | 0.99718 | 0.01 | 0.03 | 0.5 - 100 | 0.99554 | 0.02 | 0.05 |
| **Clofibric acid** | - | 0.5 - 300 | 0.99741 | 0.01 | 0.03 | 0.1 - 300 | 0.99829 | 0.01 | 0.03 |
| **Diclofenac** | - | 0.1 - 300 | 0.99840 | 0.05 | 0.16 | 5 - 300 | 0.99937 | 0.05 | 0.14 |
| **4-Hydroxydiclofenac** | + | 0.5 - 200 | 0.99595 | 0.02 | 0.06 | 0.1 - 300 | 0.99134 | 0.01 | 0.03 |
| **Diltiazem** | + | 0.05 - 100 | 0.99907 | 0.01 | 0.03 | 0.05 - 10 | 0.99486 | 0.02 | 0.05 |
| **Fenofibrate** | + | 0.25 - 200 | 0.99746 | 0.03 | 0.10 | 0.05 - 200 | 0.99084 | 0.02 | 0.05 |
| **Fipronil** | - | 0.05 - 10 | 0.99671 | 0.02 | 0.05 | 0.05 - 10 | 0.99034 | 0.02 | 0.05 |
| **Fipronil desulfinyl** | - | 0.05 - 10 | 0.99702 | 0.02 | 0.05 | 0.05 - 10 | 0.99487 | 0.01 | 0.03 |
| **Fipronil sulfone** | - | 0.05 - 10 | 0.99427 | 0.01 | 0.04 | 0.05 - 10 | 0.99198 | 0.02 | 0.05 |
| **Fluconazole** | + | 0.25 -200 | 0.99659 | 0.01 | 0.03 | 0.1 - 100 | 0.99623 | 0.01 | 0.02 |
| **Furosemide** | - | 0.5 - 300 | 0.99531 | 0.01 | 0.04 | 1 - 300 | 0.99496 | 0.02 | 0.06 |
| **Gemfibrozil** | - | 0.05 - 100 | 0.99812 | 0.01 | 0.02 | 0.5 - 100 | 0.99461 | 0.01 | 0.02 |
| **Hydrochlorothiazide** | - | 0.1 - 100 | 0.99713 | 0.02 | 0.05 | 0.1 - 100 | 0.99339 | 0.01 | 0.02 |
| **Ibuprofen** | - | 0.25 - 300 | 0.99751 | 0.01 | 0.03 | - | - | - | - |
| **Indomethacine** | - | 0.5 - 300 | 0.99753 | 0.01 | 0.02 | - | - | - | - |
| **Irbesartan** | - | 0.25 - 100 | 0.99787 | 0.01 | 0.03 | 0.1 - 10 | 0.99866 | 0.02 | 0.05 |
| **Lamotrigine** | + | 0.05 - 100 | 0.99435 | 0.02 | 0.05 | 0.05 - 100 | 0.99585 | 0.03 | 0.10 |
| **Lamotrigine N2-oxide** | + | 0.25 - 200 | 0.99591 | 0.01 | 0.02 | 0.1 - 10 | 0.99704 | 0.01 | 0.03 |
| **5-Desamino 5-Oxo-2,5-dihydro-Lamotrigine** | + | 0.25 - 300 | 0.99878 | 0.13 | 0.40 | 1 - 100 | 0.99712 | 0.02 | 0.06 |
| **N2-Methyl-Lamotrigine** | + | 0.05 - 200 | 0.99693 | 0.13 | 0.38 | 1 - 200 | 0.99072 | 0.01 | 0.04 |
| **Metoprolol** | + | 0.25 - 300 | 0.99875 | 0.03 | 0.10 | 0.25 - 300 | 0.99411 | 0.01 | 0.02 |
| **Metrodinazole** | + | 0.5 - 100 | 0.99397 | 0.11 | 0.33 | 0.5 - 300 | 0.99597 | 0.02 | 0.06 |
| **Oxcarbazepine** | + | 0.25 - 100 | 0.99346 | 0.01 | 0.02 | 0.05 - 10 | 0.99763 | 0.01 | 0.03 |
| **Propranolol** | + | 0.5 - 100 | 0.99745 | 0.02 | 0.06 | 0.5 - 300 | 0.99861 | 0.04 | 0.13 |
| **Sucralose** | - | 5 - 300 | 0.99976 | 0.01 | 0.05 | 5 - 300 | 0.99737 | 0.01 | 0.02 |
| **Sulfamethazine** | + | 0.25 - 200 | 0.99837 | 0.01 | 0.03 | 0.1 - 300 | 0.99622 | 0.01 | 0.02 |
| **Sulfamethoxazole** | - | 1 - 200 | 0.99351 | 0.01 | 0.03 | 1 - 300 | 0.99552 | 0.01 | 0.04 |
| **4-Nitro-Sulfamethoxazole** | + | 1 - 200 | 0.99715 | 0.02 | 0.07 | 0.5 - 300 | 0.99746 | 0.01 | 0.03 |
| **N4-Acetylsulfamethoxazole** | - | 5 - 300 | 0.99167 | 0.07 | 0.22 | 0.25 - 300 | 0.99802 | 0.01 | 0.03 |
| **Sulfanilamide** | - | 5 – 300 | 0.99135 | 0.01 | 0.03 | 1 - 300 | 0.99756 | 0.01 | 0.03 |
| **Sulfanilic acid** | - | 1 - 300 | 0.98147 | 0.05 | 0.12 | 0.25 - 300 | 0.97912 | 0.01 | 0.03 |
| **Valsartan** | - | 0.5 - 300 | 0.99717 | 0.01 | 0.03 | 0.05 - 200 | 0.99037 | 0.01 | 0.04 |
| **Valsartan acid** | + | 0.1 - 100 | 0.99690 | 0.04 | 0.12 | 0.25 - 300 | 0.99109 | 0.04 | 0.12 |
| **Verapamil** | + | 0.1 - 300 | 0.99814 | 0.01 | 0.03 | 0.05 - 10 | 0.99535 | 0.02 | 0.06 |

## Table S12. Concentration of organic contaminants in the wastewater used for irrigation calculated in SWATH acquisition mode

| **Compound** | **Sampling 1** | **Sampling 2** |
| --- | --- | --- |
|  | **ng L^-1^** | **ng L^-1^** |
| **Acesulfame** | **2864** | **2624** |
| **Acetaminophen** | **<LOQ** | **<LOQ** |
| **Acridone** | **1.708** | **<LOQ** |
| **Benzotriazole** | **251.8** | **65.66** |
| **5-Methyl-2H-Benzotriazole** | **<LOQ** | **<LOQ** |
| **Bezafibrate** | **<LOQ** | **<LOQ** |
| **Bisphenol A** | **<LOQ** | **<LOQ** |
| **Caffeine** | **647.6** | **731.8** |
| **Carbamazepine** | **67.04** | **66.06** |
| **Carbamazepine-10,11-epoxide** | **3.08** | **2.534** |
| **Chloramphenicol** | **<LOQ** | **<LOQ** |
| **Ciprofloxacin** | **<LOQ** | **<LOQ** |
| **Citalopram** | **7.686** | **12.312** |
| **Clarithromycin** | **12.64** | **2.55** |
| **Climbazole** | **<LOQ** | **<LOQ** |
| **Clofibric acid** | **<LOQ** | **<LOQ** |
| **Diclofenac** | **469.8** | **564.8** |
| **4-Hydroxydiclofenac** | **<LOQ** | **<LOQ** |
| **Diltiazem** | **7.286** | **6.67** |
| **Fenofibrate** | **<LOQ** | **<LOQ** |
| **Fipronil** | **<LOQ** | **<LOQ** |
| **Fipronil desulfinyl** | **<LOQ** | **<LOQ** |
| **Fipronil sulfone** | **<LOQ** | **<LOQ** |
| **Fluconazole** | **<LOQ** | **<LOQ** |
| **Furosemide** | **<LOQ** | **<LOQ** |
| **Gemfibrozil** | **<LOQ** | **<LOQ** |
| **Hydrochlorothiazide** | **<LOQ** | **<LOQ** |
| **Ibuprofen** | **<LOQ** | **<LOQ** |
| **Indomethacine** | **8.794** | **7.912** |
| **Irbesartan** | **7.938** | **6.624** |
| **Lamotrigine** | **40.98** | **40.74** |
| **Lamotrigine N2-oxide** | **<LOQ** | **<LOQ** |
| **5-Desamino 5-Oxo-2,5-dihydro-Lamotrigine** | **<LOQ** | **<LOQ** |
| **N2-Methyl-Lamotrigine** | **<LOQ** | **<LOQ** |
| **Metoprolol** | **<LOQ** | **<LOQ** |
| **Metrodinazole** | **<LOQ** | **<LOQ** |
| **Oxcarbazepine** | **<LOQ** | **<LOQ** |
| **Propranolol** | **<LOQ** | **<LOQ** |
| **Sucralose** | **89.52** | **104.06** |
| **Sulfamethazine** | **<LOQ** | **<LOQ** |
| **Sulfamethoxazole** | **74.94** | **112.64** |
| **4-Nitro-Sulfamethoxazole** | **<LOQ** | **<LOQ** |
| **N4-Acetylsulfamethoxazole** | **<LOQ** | **<LOQ** |
| **Sulfanilamide** | **<LOQ** | **<LOQ** |
| **Sulfanilic acid** | **<LOQ** | **<LOQ** |
| **Valsartan** | **96.24** | **0.8302** |
| **Valsartan acid** | **94.58** | **44.08** |
| **Verapamil** | **<LOQ** | **<LOQ** |

Approximately 20 L of wastewater were sampled from the wastewater treatment plant EDAR of El Prat de Llobregat (Barcelona, Spain) on two events, at the beginning of the experiment and after one month. In both cases the water was were filtrated under vacuum conditions through a glass microfiber filter GF/F 0.7 μm from Whatman (UK) and separated into 1-L PET bottles and frozen at -20°C. The day before each irrigation, 2 bottles were thawed at 4°C overnight and used the day following. The presence of organic contaminants was assessed at each sampling adapting a previously validated method according to (Sabater-Liesa et al. 2019, Sabater-Liesa et al. 2021). Briefly, 500 mL-samples were spiked with a mix of different labeled compounds at a concentration of 100 ng L^-1^ and were concentrated by using solid-phase extraction on 500-mg Oasis HLB cartridges (Waters, Milford, MA, US). The cartridges were eluted with 3x3 mL methanol/ethyl acetate (1:1) and reconstituted in 20 % acetonitrile.LC separation was performed using a SCIEX ExionLC™ AD system (Sciex, Redwood City, CA, U.S.) with an Acquity® UPLC BEH C18 column (100 mm x 2.1 mm i.d., 1.7 µm particle size (Waters), maintained at 40 °C. The mobile phases were (A) 5 mM ammonium acetate, 0.1% formic acid and (B) 0.1% formic acid in acetonitrile (B). Compounds were separated with a linear gradient started with 3% of B for 0.1 min and increased to 98 % in 7 min, kept constant at 98 % for 1.4 min and finally brought back to initial conditions in the following 90 s. The flow rate was 0.6 mL/min, the injection volume was 5 µL, and the auto-sampler temperature was maintained at 8 °C. A SCIEX X500R QTOF system (Sciex, Redwood City, CA) was used for data acquisition employing SWATH acquisition workflow which consisted of an MS scan over an m/z range from 100 to 950 with an accumulation time (AT) of 100 ms followed by ten MS/MS experiments with variable Q1 windows (m/z 30 to 900, 30 ms AT) and recorded using a collision energy of 35 V with an energy spread of ±15 V. The instrument provided a resolving power (FWHM) of 31,000 at m/z 132.9049 and 44,000 at m/z 829.5395 with a mass error less than or equal to 0.4 ppm.

References

Sabater-Liesa L, Montemurro N, Font C, Ginebreda A, González-Trujillo JD, Mingorance N, Pérez S, Barceló D (2019): The response patterns of stream biofilms to urban sewage change with exposure time and dilution. Science of The Total Environment 674, 401-411

Sabater-Liesa L, Montemurro N, Ginebreda A, Barceló D, Eichhorn P, Pérez S (2021): Retrospective mass spectrometric analysis of wastewater-fed mesocosms to assess the degradation of drugs and their human metabolites. Journal of Hazardous Materials 408, 124984
